# IncRNA‐*ZFAS1*, an Emerging Gate‐Keeper in DNA Damage‐Dependent Transcriptional Regulation

**DOI:** 10.1002/advs.202412385

**Published:** 2025-05-24

**Authors:** Jiena Liu, Qing Lu, Zixuan Fan, Jiahui Lin, Nan He, Xin Zhang, Zhaoya Han, Tingting Zhu, Zhenzhen Wu, Yingying Xu, Yuming Wang

**Affiliations:** ^1^ Department of Neurology Institute of Neuroscience Key Laboratory of Neurogenetics and Channelopathies of Guangdong Province and the Ministry of Education of China The Second Affiliated Hospital Guangzhou Medical University Guangzhou Guangdong 511436 P. R. China; ^2^ School of Basic Medical Sciences Southern Medical University Guangzhou Guangdong 510515 P. R. China

**Keywords:** IncRNA‐ZFAS1, nucleotide excision repair, transcription regulation, UV‐C irradiation

## Abstract

Numerous long noncoding RNAs (lncRNAs) are generated in response to external stimuli, but the scope and functions of such activity are not known. Here, this study provides insight into how the transcription of lncRNAs is connected to DNA damage response by identifying the lncRNA *ZFAS1*, which is required for cell cycle arrest, transcription regulation, and DNA repair. Mechanistically, *ZFAS1* facilitates dynamic changes in hyperphosphorylated forms of the large subunit of RNA polymerase II (RNAPII) around transcription initiation sites by directly targeting the regulated genes. It is shown that extensive transcription shutdown and concomitant stimulated engagement of RNAPII‐Ser2P are crucial for repair and cell survival upon genotoxic stress. Finally, *ZFAS1* knockout in mice dampened nucleotide excision repair (NER) and led to kidney dysplasia. Overall, the findings extend the understanding of lncRNAs in DNA damage repair (DDR) and imply a protective role of lncRNA against DDR‐deficient developmental disorders.

## Graphical abstract

LncZFAS1 plays a crucial role during DNA damage response in mammalian cells. Loss of lncZFAS1 results in deficient DNA lesion removal and reduced cell viability. Mechanistically, lncZFAS1 modulates RNAPII phosphorylation and transcription and thereby promotes both GG‐NER and TC‐NER upon UV damage.

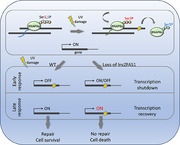

## 1 Introduction

The hereditary information encoded in the DNA sequence is intrinsically susceptible to alterations and is continually threatened by a variety of genotoxic perturbations.^[^ ^1^ ^]^ To safeguard the stability of the genome, eukaryotic cells have evolved sophisticated surveillance systems that control multiple aspects of the cellular response, including detecting DNA lesions, implementing transient cell cycle arrest, modulating transcription, and executing DNA repair.^[^ ^1^, ^2^ ^]^ To date, extensive characterization of DNA repair mechanisms has revealed their operation across different cell cycle stages and in response to distinct types of DNA lesions.^[^ ^3^ ^]^ When cells encounter stressful conditions, the DNA damage response (DDR) precisely coordinates cellular events through two parallel pathways: 1) activation of DNA damage checkpoints that impose cell cycle blockage to prevent chromosome segregation and erroneous genetic transmission^[^ ^4^ ^]^; and 2) stalling and degradation of transcribing RNAPII, which acts as DNA damage sensors to initiate repair.^[^ ^5^ ^]^ On the other hand, after DNA damage repair, timely checkpoint recovery and coordinated restoration of transcription become essential for continued cell proliferation.^[^ ^5b^ ^]^ Nevertheless, it is still poorly understood how the DNA damage checkpoints and stalled RNAPII molecules convert a very limited amount of molecular‐level information (including single DNA lesions) in the context of an otherwise genome into regulation that halts and resumes the cell‐cycle engine in a coordinated way.

Eukaryotic cells preferentially remove bulky DNA lesions such as those generated by UV irradiation from the transcribed strand of active genes by transcription‐coupled nucleotide excision repair (TC‐NER).^[^ ^6^ ^]^ In contrast, global genome repair (GG‐NER) eliminates remaining DNA lesions through a slower, non‐strand‐specific mechanism.^[^ ^7^ ^]^ As NER plays a crucial role during G1 phase, if left unrepaired, bulky DNA lesions can block DNA polymerases. Studies in rodents reveal that G2 arrest occurs specifically under conditions of functional TC‐NER combined with defective GG‐NER, indicating that TC‐NER enables replication of damaged DNA and S‐phase progression, ultimately leading to G2 arrest due to persistent DNA damage load in the genome requiring intact GG‐NER.^[^ ^8^ ^]^ As the central component of TC‐NER, the bulk of RNAPII physically shield DNA lesions from recognition by DNA repair factors^[^ ^9^ ^]^ and undergo degradation in a last‐resort pathway when TC‐NER fails.^[^ ^10^ ^]^ Beyond these well‐studied functions, recent advances have revealed unanticipated complexities in RNAPII dynamics during DNA damage. While the genome‐wide transcription shutdown paradigm (attributed to RNAPII stalling at lesions) has been widely accepted, persistent detection of stress‐induced 5′ nascent RNA activity challenges this model.^[^ ^5^, ^11^ ^]^ Mapping of chromatin state upon DNA damage illustrates a model of constant recruitment of new RNAPII molecules to maintain a reserve pool enabling genome‐wide lesion surveillance.^[^ ^12^ ^]^ This rationale is not mutually exclusive with the functions of RNAPII ubiquitination and degradation. Although multiple proteins mediate RNAPII ubiquitination in yeast and human cells,^[^ ^13^ ^]^ human‐specific functions beyond lesion clearance were only recently elucidated. Notably, site‐specific ubiquitination of RNAPII can promote the recruitment of TFIIH, mediate transcription shutdown, and initiate TC‐NER.^[^ ^14^ ^]^ Together, these findings highlight the impact of transcription on the spatiotemporal orchestration of DNA repair activities, making it imperative to explore the mechanisms in detail to unveil the causative connections between transcription, DNA damage checkpoint, and DNA repair.

The human genome transcribes numerous lncRNAs, yet few have been functionally characterized in certain cellular processes or human diseases.^[^ ^15^ ^]^ It has been suggested that many lncRNAs may represent byproducts of promiscuous transcription rather than functional molecules.^[^ ^16^ ^]^ For instance, DNA damage‐induced transcription of noncoding regulatory loci has been proposed to maintain transcription factor‐binding site in check, thereby ensuring accurate regulation of topologically associated mRNA genes.^[^ ^12^, ^17^ ^]^ To resolve this issue, systematic functional studies are required to establish the molecular mechanisms and biological relevance of specific lncRNAs. Although evidence for the function of ncRNAs as a group remains limited, several individual lncRNAs have been demonstrated to modulate DNA damage responses. The prime examples are in the genomic loci of cell cycle genes. Two lncRNAs (named *PANDA* and *DINO*) transcribed antisense to *CDKN1A* are induced by DNA damage and regulate distal genes *in trans*. *PANDA* suppresses apoptosis by blocking the transcription factor NF‐YA,^[^ ^18^ ^]^ whereas *DINO* amplifies DNA damage signaling by stabilizing p53.^[^ ^19^ ^]^ Another example is the *ASCC3* gene which produces both protein‐coding and noncoding transcripts. These isoforms antagonistically regulate transcription recovery following UV‐induced DNA damage.^[^ ^20^ ^]^ Inspired by these examples, we hypothesized that additional stress‐responsive lncRNAs function as essential components of the DNA damage response, orchestrating repair processes through mechanisms awaiting discovery.

In this study, we reveal a map of extensive lncRNA transcription dynamics during DDR using synchronized cells, leading to the unexpected identification of the poorly characterized mammalian lncRNA‐*ZFAS1* as a critical regulator. We demonstrate that *ZFAS1* functions as a key player of cellular response to DNA damage in both human and rodent cells through fine‐tuning of RNAPII kinetics, suggesting a conserved lncRNA‐dependent transcriptional regulatory axis that maintains genomic stability upon DNA damage in mammalian cells.

## 2 Results

**Figure 1:**
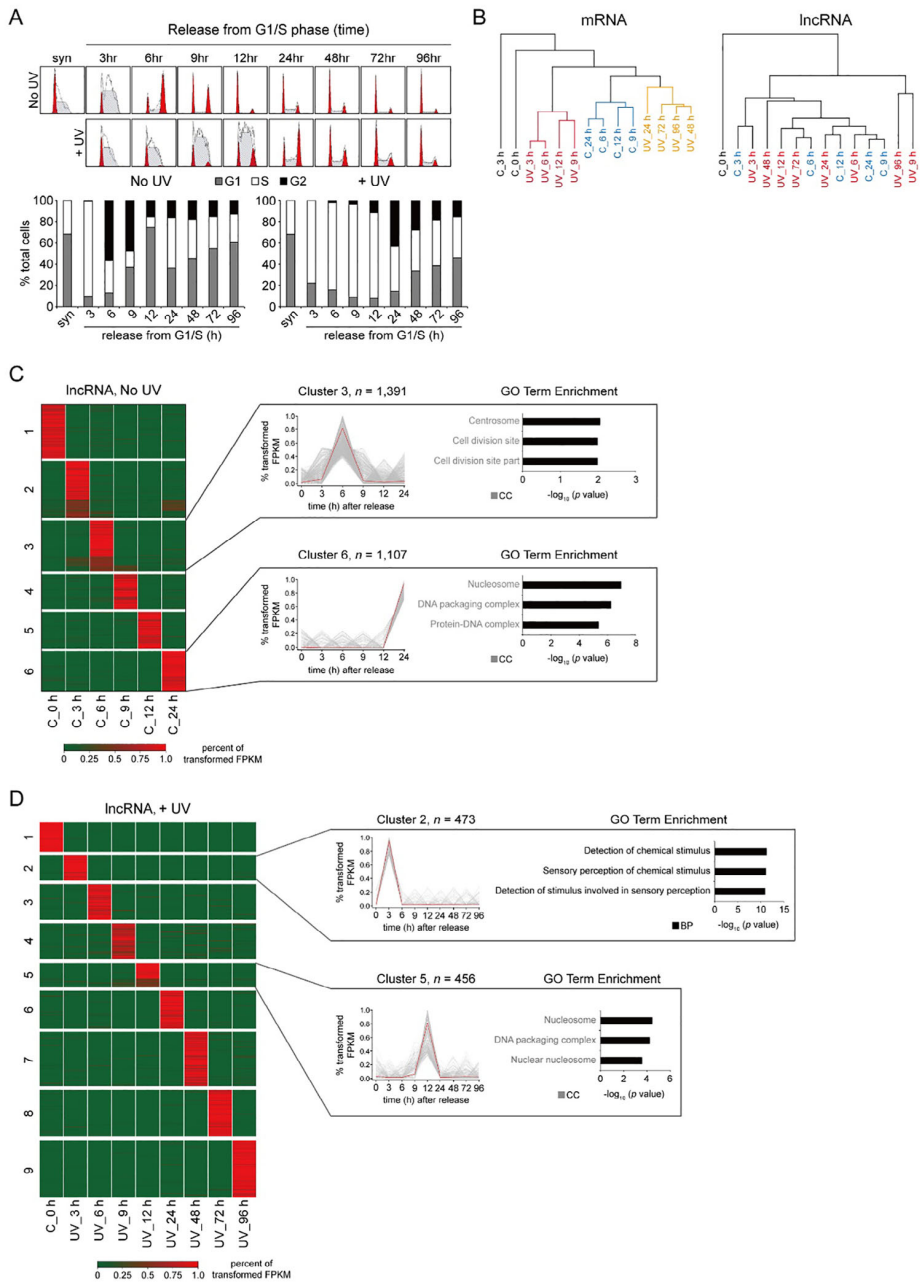
Gene expression is coordinated with cell cycle progression following UV‐C damage. A) *top*: cell cycle profiles of synchronized MRC5_VA cells treated with or without UV‐C irradiation (10 J m^−2^) prior to release from the G1/S block. *bottom*: percent of cells in each phase of the cell cycle as determined by FACS analysis at the indicated time intervals after release from the G1/S block with or without UV‐C exposure. B) Unsupervised hierarchical clustering of temporal transcriptomic data for mRNAs and lncRNAs respectively. C, control, without UV‐C treatment. C) Heatmap of transformed FPKM of temporal lncRNAs showing data from RNA‐seq of non‐irradiated MRC5_VA cells released from the G1/S block. Transcript‐wise hierarchical clustering heat map of differentially expressed lncRNAs showing segregation into six groups. Cluster 3 (*n* = 1391) includes lncRNAs upregulated at 6 h after release. Cluster 6 (*n* = 1107) includes lncRNAs upregulated at 24 h after release. Plots show a representative expression profile for the indicated clusters. Enriched GO terms that are associated with lncRNAs through lncRNA‐mRNA co‐localization and co‐expression analysis are shown to the right. C, control, without UV‐C treatment. cc, cellular component. D) The same as in (C) except that showing data from RNA‐seq of UV‐irradiated G1/S‐phase‐synchronized MRC5_VA cells. bp, biological processes.

DNA damage checkpoints activated by severe stress can trigger permanent cellular outcomes such as apoptosis or senescence.^[^ ^21^ ^]^ Conversely, failure to balance cell cycle arrest with recovery mechanisms can cause genome instability and impair cell proliferation post‐DDR.^[^ ^22^ ^]^ It has been underscored that transcriptional regulation is one important mechanism that ensures the timely activation of the DNA damage checkpoints. To investigate G1/S‐phase transcriptional networks governing cell cycle arrest and checkpoint responses, we employed synchronized human fibroblasts irradiated with 10 J m^−2^ UV‐C at the G1/S boundary. Cells were analyzed by RNA sequencing at defined post‐recovery timepoints (**Figure** **1**A). UV‐treated cells exhibited prolonged S‐phase duration and extended late S‐phase/G2 arrest (Figure 1A). This delayed cell cycle progression was precisely coordinated with the dynamic expression of the rescheduled Cyclin A and Cyclin B (Figure S1A, Supporting Information). Notably, the substantial removal of CPD (cyclobutane pyrimidine dimers) lesions from the UV‐exposed synchronized cells was accomplished within 24 hr post‐irradiation (Figure S1B, Supporting Information). Nascent RNA profiling revealed maximal transcription suppression at 3 h and full recovery by 24 h (Figure S1C, Supporting Information). This optimized cellular system enabled systematic identification of DDR‐regulatory lncRNAs through comparative transcriptomics.

### 2.1 Extensive and Regulated lncRNA Transcription of Human Fibroblasts During DNA Damage‐Induced Cell Cycle Progression

To systematically map transcription programs orchestrating DNA damage‐induced cell cycle checkpoints, we conducted RNA‐seq in UV‐irradiated G1/S‐synchronized fibroblasts (Figure S1D and Table S1, Supporting Information). Comparative analysis revealed a fundamental divergence between lncRNA and mRNA expression dynamics (Figure 1B; Figure S1E,F, Supporting Information). In particular, the discernable boundaries (or phases) that may demarcate the key stages with distinct gene expression changes during DDR were observed at the mRNA expression levels in that the irradiated cells can be partitioned into two distinguished phases: phase 1 (UV_3 h to UV_12 h) and phase 2 (UV_24 h to UV_96 h) (see the left panel in Figure 1B). In contrast, lncRNAs exhibited less pronounced phase‐segregated expression dynamics (see the right panel in Figure 1B).

First, we systematically analyzed the transcriptional dynamics of protein‐coding genes during DNA damage‐induced cell cycle progression. Without UV‐C irradiation, through hierarchical clustering of temporally regulated genes, six distinct expression clusters were identified (Figure S2A, Supporting Information). Genes that were immediately activated after release from the G1/S block (3 h; FDR < 0.05) are enriched in nucleosome assembly‐related processes, indicating an association with DNA replication during S phase. Genes induced at the early time point (6 h; FDR < 0.05) are enriched in cell‐cell adhesion and microtubule organization, indicative of the G2/M transition of the cell cycle progression (Figure S2A and Table S2, Supporting Information). Inspection of the well‐defined stage‐specific markers, such as HIST1H1D and TBP (required for S phase‐specific chromatin organization), and AURKA and CLIP2 (mediating microtubule formation during G2/M transition), confirmed their predicted cell cycle phase‐specific expression (Figure S2B, Supporting Information). Upon UV exposure, we identified 8421 stage‐specific transcripts which partitioned into seven groups based on their expression patterns across the DDR process (Figure S2C and Table S3, Supporting Information). The genes that were immediately upregulated upon UV irradiation are primarily associated with cellular response to stress (3 h after UV; FDR < 0.05). The late phase (72 h after UV; FDR < 0.05) comprised genes involved in cell morphogenesis and cell growth (Figure S2C and Table S4, Supporting Information). We subsequently surveyed the expression changes of three important genes during the DNA damage‐induced cell cycle progression by qPCR and Western blotting (Figure S3A–D, Supporting Information). These results further confirmed that expression changes of key genes related to the specific cell cycle stages during the DDR can be faithfully recapitulated in our system.

In our data, we detected several previously studied cell cycle phase‐specific lncRNAs, including *LUCAT1* and *MANCR*,^[^ ^23^ ^]^ which displayed peak expression levels during S phase (3‐6 h post‐release in non‐irradiated cells). Notably, their induction was significantly delayed upon UV exposure (24–48 h post‐release in irradiated cells) (Figure S4A, Supporting Information). The well‐defined DNA damage response lncRNAs *TP53TG1* ^[^ ^24^ ^]^ and *NEAT1* ^[^ ^25^ ^]^ were immediately activated following UV‐C irradiation, while maintaining undisturbed expression patterns during unperturbed cell cycle progression without genotoxic stress (Figure S4B, Supporting Information). These results attest to the reliability of our dataset in identifying lncRNAs involved in both cell cycle progression and DDR. Given that lncRNAs exhibit shorter half‐lives and lower stability compared to protein‐coding mRNAs,^[^ ^26^ ^]^ we hypothesized that lncRNAs functioning within narrow temporal windows would show stage‐specific activation. Our analysis identified 7464 temporally regulated lncRNAs, which could be partitioned into six clusters based on their expression patterns in the absence of genotoxic stress (Figure 1C; Table S5, Supporting Information). We further investigate the functional relevance of stage‐specific lncRNAs by analyzing biological roles of co‐expressed or co‐localized mRNAs. This analysis demonstrated that lncRNAs grouped in cluster 3 and 6 exhibited expression patterns strongly correlated with concurrent stage‐specific biological processes, showing significant enrichment of GO biological terms related to cell cycle regulation (lncRNAs in cluster 3 are associated with cytokinesis, lncRNAs in cluster 6 are associated with DNA replication; Figure 1C; Table S5, Supporting Information). Upon UV‐C irradiation, 6500 lncRNAs were identified as being upregulated at specific time points. Notably, those activated immediately (3 h post‐exposure) showed predominant involvement in DNA damage response pathways, while genes co‐localized with lncRNAs highly expressed at 12 h post‐treatment were enriched for cell cycle regulatory functions (Figure 1D; Table S6, Supporting Information). The high correlation between the dynamic expression patterns of these lncRNAs and distinct cellular conditions/phases suggests their potential functional roles in DNA damage‐associated cell cycle regulation.

To gain further insight into the nature of these temporal lncRNAs, we assessed their molecular features, including exon number and gene length. In comparison to the background exon numbers of all lncRNAs, we observed no consistent over‐ or under‐represented differences across samples (Figure S4C, Supporting Information). In contrast, stage‐specific lncRNAs exhibited distinct gene lengths: early‐stage DNA damage‐induced lncRNAs (clusters 2–4, 3–9 h upon UV‐C irradiation) and late‐stage counterparts (clusters 7–9, 48–96 h after UV‐C exposure) were generally shorter than intermediate‐phase lncRNAs activated during 12–24 h after UV‐C treatment (clusters 5–6) (Figure S4D,E, Supporting Information).

**Figure 2:**
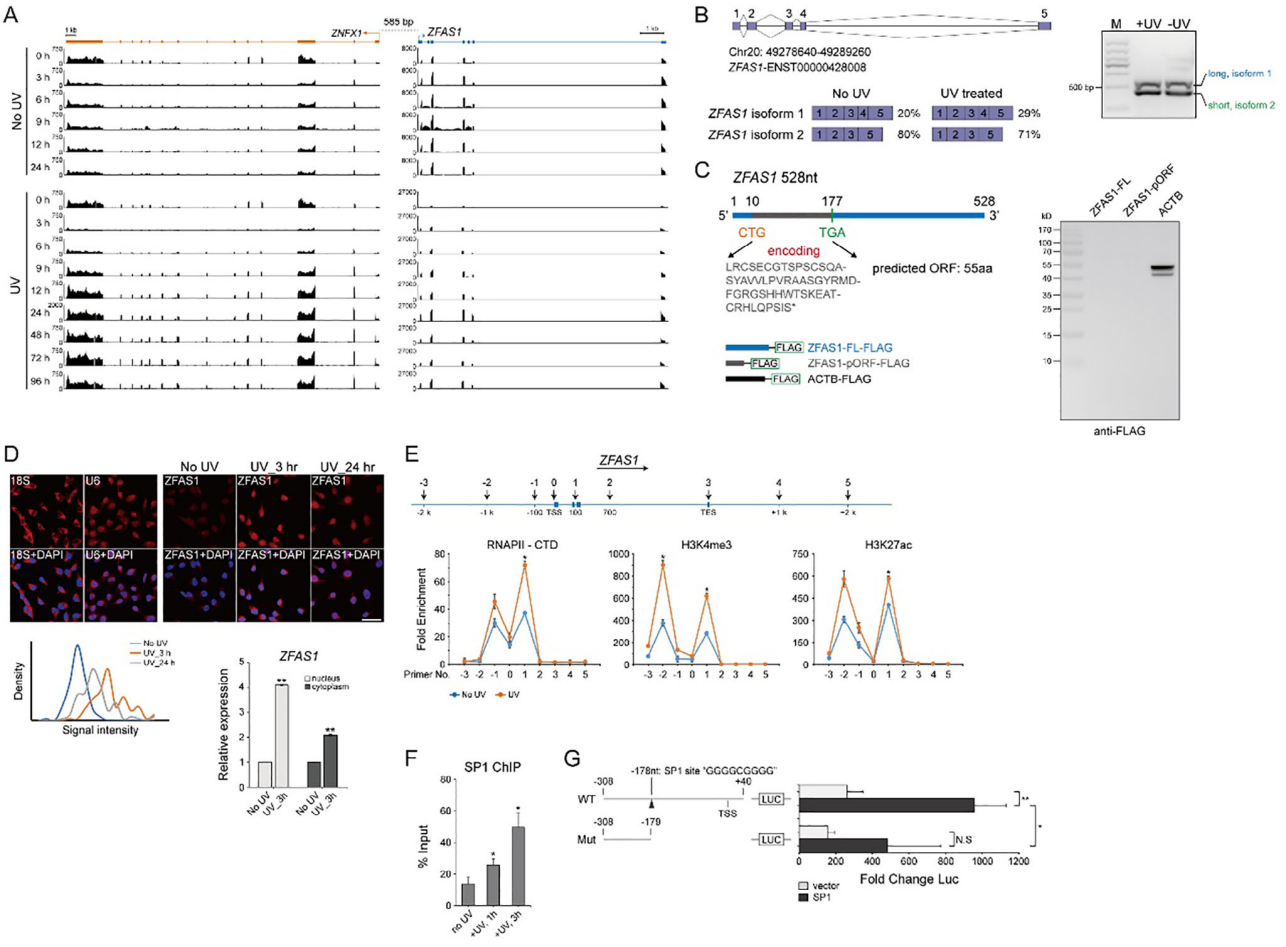
Bulky DNA lesions induce active transcription of *ZFAS1* lncRNA. A) IGV snapshot showing *ZFAS1* and its neighboring protein coding gene *ZNFX1* tracks from RNA‐seq of the synchronized MRC5_VA cells released from the G1/S block treated with or without 10 J m^−2^ UV‐C irradiation. *ZFAS1* and *ZNFX1* loci diagramed at the top. B) *left*: illustration of the full‐length *ZFAS1* transcripts (two variants). *right*: RT‐PCR validation of the two variants of *ZFAS1* in MRC5_VA cells with or without UV‐C irradiation. C) Western blot against FLAG in MRC5_VA cells for identifying the protein‐coding capacity of the full length and the putative ORF in *ZFAS1* lncRNA. The grey box represents the putative ORF. The ACTB‐FLAG was used as a positive control. pORF, predicted ORF. D) *top*: representative confocal FISH images showing localization of *ZFAS1* in unsynchronized MRC5_VA cells treated with or without UV‐C irradiation. U6 indicates the probes for U6 snRNA, 18S indicates the probes for 18S rRNA. Scale bar = 50 µm. Histogram plots of average *ZFAS1* expression signals following UV‐C irradiation. No UV stands for without UV treatment. *bottom*: RT‐PCR detection of *ZFAS1* in the cytoplasmic and nuclear fractions of the WT cells with or without UV‐C irradiation. Each data bar is presented as the means ± SD from three independent experiments. **p < 0.01 compared to the non‐irradiated cells (Student's *t*‐Test). E) *top*: a schematic representation of *ZFAS1* locus, which was adapted from UCSC Genome Browser. Numbered arrows indicate the genomic regions analyzed for RNAPII binding and histone modifications using ChIP assays. *bottom*: ChIP‐qPCR analysis showing the binding of RNAPII‐CTD and the indicated histone marks, at the *ZFAS1* locus in the untreated and UV‐irradiated MRC5_VA cells (at 3 h post‐irradiation), plotted relative to IgG controls. Error bars represent ± SD from three independent experiments. Student's *t*‐Test, *p < 0.05. F) SP1 occupancy on the regulatory regions of *ZFAS1* was analyzed by ChIP‐qPCR. Normalized data are shown as percentage of input control. Error bars represent ± SD (*n* = 3 replicates). *p < 0.05 (Student's *t*‐Test). G) Luciferase reporter assay: the *ZFAS1* promoter‐proximal region (−308 to +40 bp relative to TSS) or deletion variant without the SP1 binding motif (−308 to −179 bp relative to TSS) was cloned upstream of the firefly luciferase coding region. The luciferase activities were tested in HEK293T cells co‐transfected with SP1 expression vector or empty vector as control. Data were normalized to renilla luciferase. *p < 0.05, and **p < 0.01 (Student's *t*‐Test). Mut, mutant; N.S, non‐specific.

To investigate the functional relevance of these DNA damage‐induced lncRNAs, we manually surveyed those stage‐specific candidates and selected three (*SNHG15*, *SLC25A34‐AS1*, and *ZFAS1*) based on their abundance and dynamic expression profiles. All three showed RT‐PCR‐validated periodic cell cycle expression patterns, corroborating our dataset analysis (Figure S5A, Supporting Information). Further examination of their nearby protein‐coding genes yielded *ZFAS1* as a particularly interesting DNA damage‐responsive lncRNA (**Figure** **2**A; Figure S5B, Supporting Information). *ZFAS1* displayed immediate activation upon genotoxic stress, peaking at 24 h post UV‐C irradiation (corresponding to cell cycle recovery following DNA repair). Strikingly, its antisense protein‐coding counterpart *ZNFX1* showed a sharp expression decline after UV‐C exposure (Figure 2A; Figure S5C,D, Supporting Information), contrasting with *ZFAS1*’s induction pattern.

### 2.2 ZFAS1 is a Conserved, DNA Damage‐Induced lncRNA

*ZFAS1* is located ≈583 base pairs upstream of the *ZNFX1* TSS and exhibits modest conservation across multiple mammalian species, underscoring its potential functional importance (Figure S6A, Supporting Information). Rapid amplification of cDNA ends (RACE) revealed two transcript variants of *ZFAS1* [528 and 466 nucleotides (nt)] (Figure S6B,C, Supporting Information), of which the 466‐nt short variant was predominantly expressed, both in the presence and absence of genotoxic stress (Figure 2B; Figure S6D, Supporting Information). RNA‐seq data from the NONCODE project^[^ ^27^ ^]^ also demonstrated high expression of the *ZFAS1* short variant in various human tissues (Figure S6E, Supporting Information). Like most intergenic lncRNAs, *ZFAS1* exhibited very low protein‐coding potential (Figure S7A, Supporting Information). To further validate its non‐coding nature, a putative ORF in *ZFAS1* was predicted using the ORF finder tool (http://www.ncbi.nlm.nih.gov/orffinder/). Western blot analysis confirmed that neither the full‐length CDS nor the putative ORF of *ZFAS1* had coding ability (Figure 2C; Figure S7B–D, Supporting Information). The cellular distribution of *ZFAS1* was then evaluated by FISH and quantitative RT‐PCR at the indicated recovery time after UV‐C exposure. In unsynchronized MRC5_VA cells, *ZFAS1* was found to be localized in both the nucleus and cytoplasm, independently of DNA damage (Figure 2D). This was further confirmed quantitatively by RNA‐seq analysis of chromatin‐ and cytoplasm‐fractionated cells (Figure S7E, Supporting Information).^[^ ^28^ ^]^ *ZFAS1* expression was also induced upon cisplatin damage (forming DNA adducts as UV damage does and also triggering NER), albeit at lower levels. In contrast, treatment with antitumor agents (5‐FU or Doxorubicin) did not affect *ZFAS1* expression (Figure S7F,G, Supporting Information). Additionally, UV damage‐induced activation of *ZFAS1* expression was observed in both IMR‐90 primary human fibroblasts and A549 lung cancer cells (Figure S7H, Supporting Information).

Given that *ZFAS1* activation was specific and fairly stable upon UV‐C exposure, we further investigated whether this resulted from transcriptional changes during DNA damage response. We performed ChIP‐qPCR to examine major histone modifications and RNAPII occupancy in both undisturbed and UV‐irradiated MRC5_VA cells. Following UV‐C exposure, we observed increased RNAPII occupancy around the *ZFAS1* TSS site (Figure 2E). Additionally, two histone modifications associated with active transcription‐H3K4me3 and H3K27Ac‐were significantly enriched in the promoter‐proximal region of *ZFAS1* during the DNA damage response (Figure 2E). These findings align with our previous ATAC‐seq data,^[^ ^29^ ^]^ which showed enhanced chromatin accessibility near the *ZFAS1* TSS after UV‐C treatment (Figure S7I, Supporting Information). Next, we attempted to specify the transcription factor responsible for *ZFAS1* activation after DNA damage. In the *ZFAS1* promoter‐proximal region, we identified a canonical SP1 binding motif (‐178 to ‐170 bp relative to TSS). SP1 is a known regulator of DNA repair.^[^ ^30^ ^]^ ChIP‐qPCR confirmed UV‐induced SP1 binding to this region in MRC5_VA cells (Figure 2F), suggesting SP1's involvement in DNA damage‐dependent *ZFAS1* transcription. We subsequently cloned *ZFAS1* core promoter regions containing either wild‐type or mutated SP1 binding motifs upstream of a firefly luciferase reporter (Figure 2G). The result clearly showed that luciferase activity was significantly higher in constructs with intact SP1 motifs and could be further augmented by SP1 overexpression (Figure 2G). These results indicate that SP1 drives *ZFAS1* expression in response to DNA damage. While no detectable changes in *SP1* expression were observed during UV‐induced DNA repair (Figure S7J, Supporting Information), suggesting that *ZFAS1* upregulation likely results from enhanced SP1 binding activity rather than increased protein abundance.

### 2.3 Knockdown of *ZFAS1* Impairs NER

**Figure 3:**
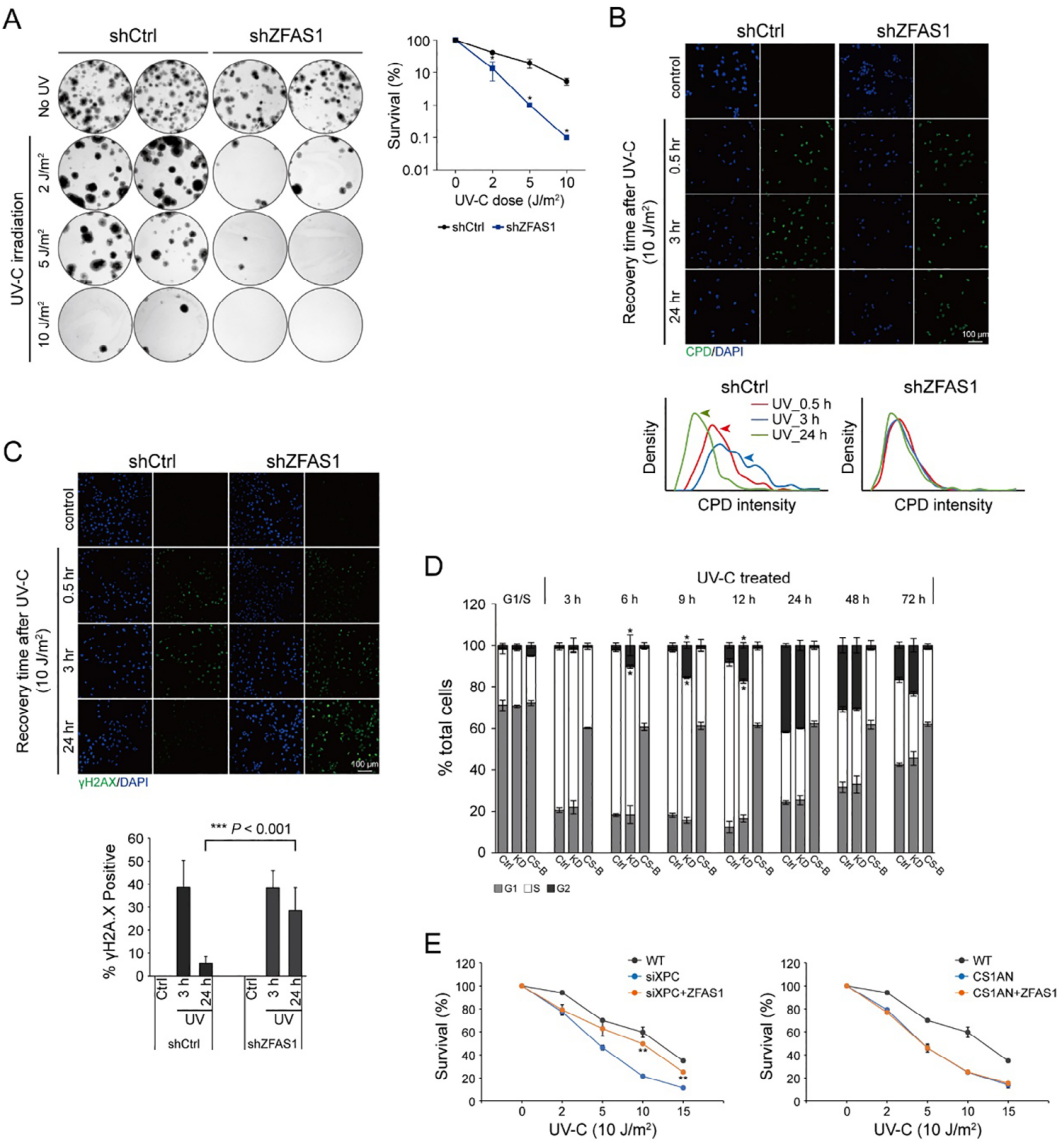
*ZFAS1* loss of function results in deficiency in NER. A) *left*: representative images of clonogenic survival assays of *ZFAS1*‐depleted MRC5_VA cells under the indicated UV‐C doses. *right*: percentage of surviving cells (logarithmic scale) plotted against UV‐C dose. Error bars indicate the standard error of the mean from three independent experiments. *p < 0.05 compared to the WT cells (Student's *t*‐Test). B) *top*: representative images of cells showing repair of CPDs at the indicated recovery time post UV irradiation using a specific antibody to CPDs (green signal) for the wild‐type and *ZFAS1*‐depleted MRC5_VA cells. DAPI‐stained nuclei in blue. Scale bar = 100 µm. *bottom*: histogram plots of average CPD signals at the indicated recovery time points following UV‐C irradiation. C) *top*: representative images of cells showing nuclear γH2A.X foci using a specific antibody to H2A.X phosphorylation (green signal) for the asynchronized wild‐type and *ZFAS1*‐depleted MRC5_VA cells at the indicated recovery time post‐irradiation. Nuclear DNA was counterstained with DAPI in blue. Scale bar = 100 µm. *bottom*: quantification of percent (%) γH2A.X‐positive (≥ 5 foci/nucleus) nuclei for each cell type, treatment and time point. A minimum of 500 cells per cell line per condition were analyzed. Each data point is presented as the means ± SD, *n* = 3. ***p < 0.001 (Student's *t*‐Test). D) Bar graph representation of the flow cytometry profiles at the indicated time intervals after release from the G1/S block of control (wild‐type) cells and *ZFAS1*‐depleted cells following UV‐C irradiation. Each data point is presented as the means ± SD, *n* = 3. *p < 0.05 compared to the wild‐type cells (Student's *t*‐Test). CS‐B cells (CS1AN) were treated as positive control for TC‐NER deficient cells which can not recover cell cycle upon UV damage. E) Cell viability of WT, CS‐B, CS‐B compensated with *ZFAS1*, XPC‐depleted cells and XPC‐depleted cells compensated with *ZFAS1* at day 3 after treatment with the indicated doses of UV‐C irradiation. Error bars indicate standard error of the mean from three independent experiments. **p < 0.01 by comparing XPC‐depleted cells and its *ZFAS1*‐compensated cells (Student's *t*‐Test).

To investigate the role of *ZFAS1* in the cellular response to DNA damage, lentivirus‐mediated RNA interference (RNAi) was used to knock down *ZFAS1* in human fibroblast cells (Figure S8A, Supporting Information). *ZFAS1*‐depleted cells exhibited hypersensitivity to UV‐C irradiation (**Figure** **3**A). Accordingly, *ZFAS1* overexpression significantly enhanced cell viability under DNA damage conditions compared to wild‐type controls (Figure S8B,C, Supporting Information). Intriguingly, however, *ZFAS1*‐depleted cells displayed tolerance to oxidative DNA damage induced by potassium bromate (Figure S8D, Supporting Information), suggesting that *ZFAS1* is dispensable for BER (base excision repair). Subcellular localization analysis revealed that overexpressed *ZFAS1* was predominantly localized to the nucleus and perinuclear region (Figure S8E, Supporting Information). Genome‐wide repair kinetic studies indicate that UV‐induced (6‐4) photoproducts (6‐4PPs) are rapidly repaired, whereas cyclobutane pyrimidine dimers (CPDs) typically require 12–48 h for complete removal by NER.^[^ ^31^ ^]^ Given this temporal resolution, CPDs serve as more reliable markers for assessing NER capacity. Our results showed that *ZFAS1*‐depleted MRC5_VA cells failed to accomplish repair of either 6‐4PPs or CPDs within 24 h post‐UV‐C irradiation (Figure 3B; Figure S8F, Supporting Information). Conversely, *ZFAS1*‐overexpressing cells were more proficient in the removal of CPD lesions compared to wild‐type cells (Figure S8G, Supporting Information).

The γH2A.X foci (phosphorylation of histone variant H2A.X at serine 139) are formed presumably at sites of DSBs (DNA double strand breaks).^[^ ^32^ ^]^ Although UV‐C exposure does not directly induce DSBs, it has been reported that γH2A.X is triggered by UV‐generated DNA repair intermediates across all cel cycle phases,^[^ ^33^ ^]^ with GG‐NER pathway rather than TC‐NER‐primarily mediating H2A.X phosphorylation.^[^ ^34^ ^]^ Consequently, γH2A.X kinetics have become established indicators for monitoring replication and transcription stress. In line with previous findings, wild‐type cells exhibited rapid γH2A.X induction (detectable within 30 min), progressive accumulation through 3 h, and resolution by 24 h post‐irradiation (Figure 3C). While *ZFAS1*‐depleted MRC5_VA cells showed comparable early‐phase H2A.X phosphorylation levels to wild‐type controls, they maintained persistently elevated γH2A.X signals at 24 h post‐UV treatment (Figure 3C). Consistent with this observation, this sustained DNA damage phenotype in *ZFAS1*‐depleted cells was further corroborated by alkaline comet assays revealing prolonged DNA strand breaks (Figure S8H, Supporting Information). These results collectively suggest that defective DNA repair in *ZFAS1*‐depleted cells leads to unresolved replication/transcription stress.

DNA‐damage checkpoints play a crucial role in sensing DNA damage and orchestrating transient cell cycle arrest to facilitate lesion repair. To test the requirement for *ZFAS1* in DNA damage‐induced cell cycle arrest, asynchronous wild‐type or *ZFAS1*‐depleted MRC5_VA cells were blocked at the G1/S boundary, and then exposed by UV‐C irradiation prior to S‐phase release. Flow cytometry analysis showed that wild type cells exhibited prolonged S‐phase arrest (Figure 3D, evident as a large cell population retained in S‐phase from 3–9 h post‐release under U‐C treatment). Strikingly, knockdown of *ZFAS1* resulted in diminished S‐phase retention with accelerated progression into G2 phase (Figure 3D, notably G2‐phase entry within 3–6 h post‐release). These findings strongly suggest that *ZFAS1* is essential for proper DNA damage checkpoint activation. Notably, *ZFAS1* depletion also caused baseline cell cycle dysregulation independent of DNA damage, as evidenced by delayed G2/M transition in unirradiated cells (Figure S8I, Supporting Information). Mechanistically, *ZFAS1* may promote lesion correction through checkpoint potentiation rather than direct repair activity. Importantly, *ZFAS1* is also required in NER in both IMR‐90 primary human fibroblasts and A549 lung cancer cells (Figure S8J,K, Supporting Information).

To investigate GG‐NER capacity in *ZFAS1*‐depleted cells, we performed unscheduled DNA synthesis (UDS) assays using a validated non‐radioactive method incorporating 5‐Ethynyl‐2′‐deoxyuridine (EdU).^[^ ^35^ ^]^ This approach effectively distinguishes repair synthesis from replicative DNA synthesis through differential nuclear fluorescence intensities (Figure S8L, Supporting Information, arrows indicate weak fluorescence nuclei representing UDS). This result showed comparable UDS activity between normal fibroblasts and Cockayne syndrome B (CS‐B, TC‐NER‐deficient) cells post‐UV exposure. However, *ZFAS1*‐depleted cells showed moderate UDS reduction compared to severely impaired xeroderma pigmentosum C (XP‐C, GG‐NER‐deficient) cells (Figure S8L, Supporting Information), indicating dual NER pathway impairment. In order to correct for non‐specific EdU incorporation and accurate exclusion of replicating cells with scheduled EdU incorporation, cells were exposed to local UV‐C irradiation through a micropore filter.^[^ ^36^ ^]^ ZFAS1 depletion significantly reduced TCR‐UDS levels (Figure S8M, Supporting Information). Notably, *ZFAS1* overexpression partially rescued UV sensitivity in XP‐C cells (particularly at higher doses), but not in CS‐B cells (Figure 3E), providing functional evidence for *ZFAS1*’s primary role in GG‐NER.

### 2.4 *ZFAS1* Functions by Modulating Global Transcription Shutdown Upon DNA Damage

**Figure 4:**
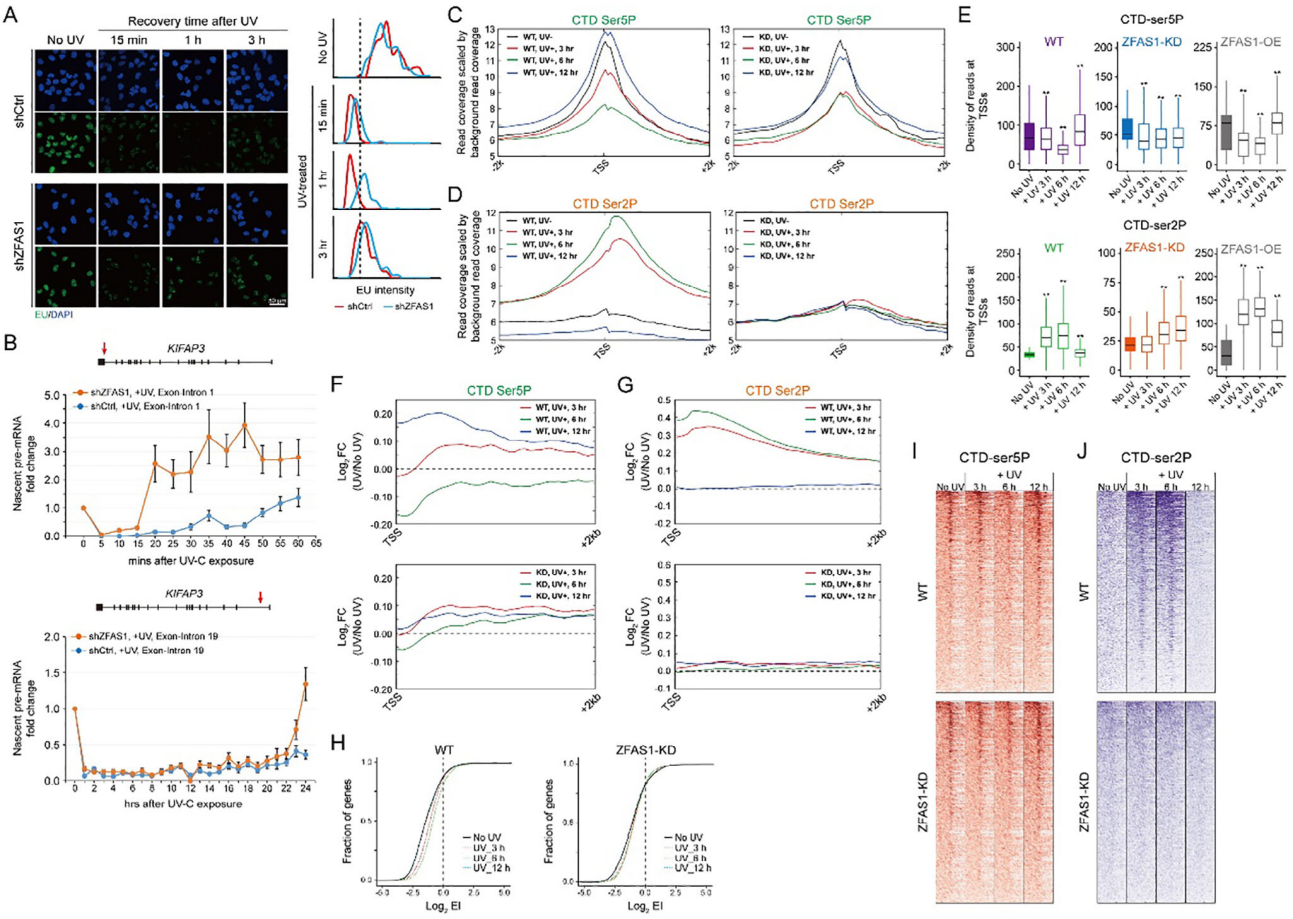
Functions of *ZFAS1* in modulating cellular transcriptional response to UV‐C irradiation. A) *left*: representative images of the wild‐type and *ZFAS1*‐depleted MRC5_VA cells by 15 mins, 1 h and 3 h after UV‐C irradiation (10 J m^−2^). Nascent EU‐labeled RNA transcripts shown in green and DAPI‐stained nuclei in blue. Scale bar = 50 µm. *right*: histogram plots of average EU incorporation in the wild‐type and *ZFAS1*‐depleted cells after UV‐C irradiation. Black stippled line demarcates lowly and normally transcribing cells, respectively. B) Nascent mRNA production in different regions of the human *KIFAP3* gene after release from DRB‐inhibition. Cells were irradiated with 10 J m^−2^ UV‐C prior to DRB removal. Error bars represent means ± SD from three independent experiments. C) Average plots of read densities for RNAPII‐Ser5P on all genes from 2 kb upstream of TSS to TSS + 2 kb, before (UV‐) and after (UV+) UV‐C irradiation for the WT cells and *ZFAS1*‐depleted cells. D) Same as in (C), but showing the read densities for RNAPII‐Ser2P. E) Boxplots summarizing quantifications of RNAPII‐Ser5P and RNAPII‐Ser2P ChIP‐seq reads shown for all TSSs genome‐wide in the WT, *ZFAS1*‐depleted MRC5_VA cells and cells overexpressing *ZFAS1* at the indicated recovery time intervals upon UV‐C exposure. Boxplots show the 25th‐75th percentiles, and error bars represent data range to the larger/smaller value. *p* values were calculated with Mann‐Whitney *U* test and were correlated by the Benjamini‐Hochberg method. **p < 2.22e‐16. F) Average plots of read densities for RNAPII‐Ser5P shown by the difference after UV irradiation at the indicated time intervals compared to the untreated samples for the WT cells and *ZFAS1*‐depleted cells (Log_2_ FC = read density UV+/read density UV‐). G) Same as in (F), but showing the difference in binding profiles of RNAPII‐Ser2P. H) RNAPII‐Ser5P escape indexes (EI) from the promoter‐proximal regions before and after UV‐C irradiation plotted as empirical cumulative distribution of all genes. I) Heatmaps from ChIP‐seq data showing RNAPII‐Ser5P binding events before (No UV) and at 3 h, 6 h, and 12 h post UV (+ UV) for genomic regions 5 kb around peaks in the WT and *ZFAS1*‐depleted MRC5_VA cell lines. Data aligned to untreated RNAPII‐Ser5P peak position. J) Same as in (I), but showing RNAPII‐Ser2P binding events.

Cells carrying mutations in certain DNA repair genes are unable to recover RNA synthesis from damaged genes and unleash attenuated RNAPII transcription elongation after UV treatment, whereas wild‐type cells regain transcriptional activity within hours post‐UV irradiation.^[^ ^5^, ^37^ ^]^ Since the signature of *ZFAS1*‐depleted cells was reminiscent of DNA repair factor deficiency under genotoxic stress, we hypothesized that *ZFAS1*‐like canonical repair factors such as XPF and CSB^[^ ^38^ ^]^ may participate in the global transcriptional response to UV‐C irradiation. To assess global transcription dynamics, cells were pulse‐labeled with 5‐ethynyl‐uridine (EU) at 3 h and 24 h after UV‐C irradiation for 30 min. EU staining of nascent transcripts revealed near‐complete transcriptional suppression across the genome at 3 h post‐UV irradiation in both wild‐type and *ZFAS1*‐depleted cells. Unexpectedly, transcription levels were tellingly fully restored in both cell types by 24 h after UV‐C exposure (Figure S9A, Supporting Information). To dissect *ZFAS1*’s role in early transcriptional regulation after DNA damage, we monitored nascent RNA synthesis at shorter intervals (15 min to 3 h post‐UV). Wild‐type cells exhibited immediate transcriptional repression within 15 min of UV‐C exposure, with further suppression evident by 1 h (**Figure** **4**A). Strikingly, *ZFAS1* knockdown cells displayed aberrantly elevated global transcription at early timepoints, evidenced by reduced proportions of low‐transcribing cells and increased EU incorporation (manifested as a rightward histogram shift) within 1 h post‐irradiation compared to wild‐type controls (Figure 4A, blue curve). This unanticipated and inspiring role of *ZFAS1* in transcription suppression post‐damage was validated via qRT‐PCR coupled with DRB synchronization. Cells pretreated with the transcription elongation inhibitor DRB were UV‐irradiated, followed by DRB washout to synchronize RNAPII release. Consistent with prior reports,^[^ ^39^ ^]^ in unirradiated wild‐type cells, RNAPII progressed to the first exon‐intron junction of *KIFAP3* gene within 20 min post‐DRB removal and reached a junction 153 kb downstream of the TSS by 2 h (Figure S9B, Supporting Information). UV‐C irradiation severely impeded RNAPII progression, with minimal advancement to the first exon‐intron junction by 50 min and delayed arrival at distal junctions (>24 h) (Figure 4B). Tellingly, *ZFAS1*‐depleted cells exhibited accelerated RNAPII recovery, reaching the first and last exon‐intron junctions by 20 min and 22 h post‐damage, respectively (Figure 4B). Intriguingly, the effect of *ZFAS1* on global transcription in the absence of exogenous genotoxic stress was the opposite of that observed upon DNA damage (Figure S9B, Supporting Information, comparing purple to red graphs, green to blue graphs), aligning with its differential effects on cell cycle progression (Figure S8I, Supporting Information). Collectively, these results establish *ZFAS1* as a bifunctional regulator of transcription, exerting opposing effects on gene expression contingent upon DNA damage status.

To further investigate whether *ZFAS1* influences the RNAPII elongation rate in response to DNA damage, we implemented DRB/GRO‐seq to map RNAPII positions across gene bodies at sequential timepoints following DRB removal, enabling synchronized transcriptional restart and genome‐wide tracking. Representative data from two long genes (*PPP1R12A* and *CTNNBL1*) are shown in Figure S9C (Supporting Information). In untreated wild‐type cells, RNAPII progressed further into genes at 10 min and 40 min after DRB removal. Upon UV‐C irradiation, the progress of RNAPII into genes was prohibited and very slow (Figure S9C, Supporting Information), consistent with prior observations.^[^ ^20^ ^]^ Strikingly, this pattern contrasts with *ZFAS1*‐depleted cells. While, RNAPII “wave‐front” positions in *ZFAS1*‐knockdown cells remained comparable to wild‐type at 10 min post‐DRB removal under both untreated and UV‐C‐treated conditions (Figure S9D, Supporting Information), a dramatic acceleration of elongation became evident by 40 min post‐UV exposure. Specifically, the wave‐fronts in *ZFAS1*‐deficient cells advanced substantially further along gene bodies at this later timepoint (Figure S9D, Supporting Information).

### 2.5 *ZFAS1* Regulates the Distribution of Ser2P‐ and Ser5P‐Modified RNAPII Upon UV‐C

Our initial observation that *ZFAS1*‐deficient cells exhibited accelerated transcriptional recovery post‐UV irradiation despite defective DNA damage repair presented an apparent paradox. ChIP‐Western analysis confirmed normal recruitment of NER proteins (XPA, CSB, and PCNA) to RNAPII‐stalled damage sites in *ZFAS1*‐knockdown cells (Figure S9E, Supporting Information), excluding impaired repair complex assembly as the primary defect. To delineate genome‐wide transcriptional reprogramming under genotoxic stress, RNAPII CTD phosphorylation state‐specific ChIP‐seq was performed in the wild‐type and *ZFAS1*‐depleted MRC5_VA cells. Without UV‐C irradiation, the distribution of RNAPII‐Ser5P was comparable between the wild‐type and *ZFAS1*‐depleted cells (Figure S9F,G, Supporting Information), indicating that the effect that *ZFAS1* deficiency slows down general transcription as signified in DRB treatment assay (Figure S9B, Supporting Information) does not lie under a decrease of pre‐initiating RNAPIIo levels. At 3 h after UV‐C exposure, in line with previously reported UV‐induced RNAPII promoter clearance dynamics,^[^ ^12^ ^]^ wild‐type cells exhibited progressive RNAPII‐Ser5P depletion from promoter‐proximal regions, reaching minimal occupancy at 6 h, followed by partial recovery by 12 h (Figure 4C, WT curves). Strikingly, *ZFAS1*‐depleted cells failed to sustain transcriptional repression at 6 hr post‐UV, showing attenuated RNAPII‐Ser5P loss compared to wild‐type (Figure 4C, *ZFAS1*‐KD curves), implying a potential inefficient transcription arrest. Concurrently, an increased concomitant enrichment of RNAPII‐Ser2P downstream of TSS sites was observed in the WT cells at 3 h and 6 h after UV stress (Figure 4D, WT curves). These results mirror previous analyses,^[^ ^14b^ ^]^ but were in striking contrast to those obtained in cells when *ZFAS1* was knocked down. Here, the RNAPII‐Ser2P occupancy was unchanged at all time points in cells lacking *ZFAS1* upon UV‐C exposure (Figure 4D, *ZFAS1*‐KD curves). Taking into consideration recent evidence that supports the model of DNA damage‐induced continuity of transcription initiation of short RNA transcripts,^[^ ^5^, ^11^, ^12^ ^]^ it is tempting to assume that the immediate loss of RNAPII‐Ser5P and concomitant accumulation of RNAPII‐Ser2P at TSSs (or rapid exchange of RNAPIIo forms) observed in wild‐type cells would facilitate continuous recruitment of lesion‐scanning factors to transcriptionally active DNA. Thus, the impaired transcriptional reprogramming in *ZFAS1*‐depleted cells during early damage response likely underlies their DNA repair deficiency (Figure 4E). To quantify RNAPII CTD phosphorylation dynamics, we calculated log2 fold changes in chromatin‐bound RNAPII‐Ser5P/Ser2P between UV‐treated and untreated cells (Figure 4F,G; Figure S9H,I, Supporting Information). Wild‐type cells displayed substantial RNAPII‐Ser5P depletion from promoters and progressive RNAPII‐Ser2P accumulation downstream of TSSs after UV irradiation (Figure 4F,G, upper panels). This reorganization of RNAPIIo forms was absent in *ZFAS1*‐depleted cells (Figure 4F,G, lower panels). We further analyzed promoter escape efficiency using the traveling ratio (gene body/promoter RNAPII‐Ser5P).^[^ ^5^, ^39^ ^]^ In accordance with the previous observations, a transient yet significant increase of promoter escape indexes at early time points during transcription recovery after UV stress (3 to 6 h post‐irradiation) was detected only in wild‐type cells (Figure 4H). Accordingly, the results of our genome‐wide analysis were consistent with the evidence obtained by peak calling of RNAPII phosphorylation forms, showing that in the wild‐type cells, RNAPII‐Ser5P levels were reduced at 3 h to 6 h and restored at 12 h (Figure 4I, upper panels; Table S7, Supporting Information). Meanwhile, RNAPII‐Ser2P levels were elevated drastically at early time points and returned to baseline at 12 h in response to UV‐C exposure (Figure S4J, upper panels, and Table S7, Supporting Information). Reciprocally, there was no obvious loss of RNAPII‐Ser5P or gain of RNAPII‐Ser2P peaks in cells lacking *ZFAS1* during the post‐UV period (Figure 4I‐J, lower panels). Complementary *ZFAS1* overexpression experiments revealed inverse effects. Gratifyingly, *ZFAS1* overexpressing cells enhanced RNAPII‐Ser5P clearance from promoters and amplified RNAPII‐Ser2P deposition near TSSs post‐UV compared to wild‐type controls (Figure 4E; Figure S9J,K, Supporting Information). Interestingly, these patterns contrast with TC‐NER‐deficient models showing persistent RNAPII‐Ser5P loss and delayed Ser2P accumulation,^[^ ^14b^ ^]^ suggesting distinct TC‐NER impairment mechanisms. More importantly, the results of our genome‐wide analysis were consistent with biochemical validation obtained by chromatin extraction followed by Western Blot analysis, showing that *ZFAS1* depletion stabilized total CTD‐Ser5P levels while impairing CTD‐Ser2P acquisition during early recovery (Figure S9L, Supporting Information). Collectively, our multi‐omics approach establishes *ZFAS1* as a critical modulator of RNAPII CTD phosphorylation cycling during DDR.

Taken together, these results further consolidate previously reported evidence^[^ ^12^, ^14^ ^]^ and reveal an unanticipated functional mechanism: *ZFAS1* modulates a transcription‐dependent defense system by differentially regulating RNAPII phosphorylation states during DNA damage. Specifically, maintaining low RNAPII‐Ser5P levels at promoter‐proximal regions proves crucial to mitigate threats to genome instability, while simultaneously activating elongating RNAPII‐Ser2P to enhance damage‐sensing efficiency. Through this dual regulatory mechanism, *ZFAS1* dynamically coordinates distinct RNAPII operational forms (RNAPIIo) to preserve genomic integrity and facilitate DNA lesion repair.

### 2.6 Increased Genomic Occupancy of *ZFAS1* is Associated with Dynamic Distribution of RNAPIIo Upon DNA Damage

Notably, although *ZFAS1* depletion disrupted the genome‐wide reorganization of RNAPIIo phosphorylation states induced by DNA damage, it exerted minimal effects on global mRNA levels following UV stress. Initial analysis demonstrated that *ZFAS1* knockdown did not alter the expression of its neighboring protein‐coding gene *ZNFX1* (Figure S10A,B, Supporting Information). Principal component analysis (PCA) from RNA‐seq segregated all samples into three distinct clusters independent of cell origin, with the primary separation axis corresponding to treatment conditions (Figure S10C, Supporting Information). Crucially, *ZFAS1*‐depleted cells displayed transcriptional responses to DNA damage comparable to wild‐type cells, as evidenced by similar numbers of differentially expressed genes (Figure S10D, Supporting Information). Furthermore, the most enriched GO‐terms for UV‐induced upregulated genes in WT cells were conserved in *ZFAS1*‐depleted lines (Figure S10E, Supporting Information). Importantly, *ZFAS1* knockdown did not impair the activation of canonical DNA damage response genes (Figure S10F, Supporting Information).

We surmised that the recurring arrested RNAPII Ser5P at promoter‐proximal regions post‐irradiation could be key to understanding *ZFAS1*’s functional mechanism, so we initially focused on damage‐repressed genes. We investigated ATF3 – a known regulator of UV‐induced late‐response genes.^[^ ^40^ ^]^ RNA‐seq analysis revealed that >30% of genes downregulated 3 h post‐UV in both WT and *ZFAS1*‐depleted cells were ATF3 targets (Figure S10G, left, Supporting Information). Concurrently, ChIP‐seq data showed that >20% of genes displaying reduced RNAPII‐Ser5P occupancy at 3 h post‐UV across both cell lines were ATF3‐regulated (Figure S10G, right, Supporting Information). However, *ZFAS1* depletion neither affected ATF3 activation nor its degradation during DDR (Figure S10H, Supporting Information). These results suggest that *ZFAS1* is not involved in ATF3‐dependent transcriptional repression after DNA damage.

Previous studies have placed pTEFb complex (CDK9 and its cyclins) at the core of the transcription‐related DNA damage response.^[^ ^41^ ^]^ As a kinase targeting serine 2 residues in RNAPII CTD repeats,^[^ ^42^ ^]^ we have reasons to speculate that the damage‐induced concurrent gain of RNAPII‐Ser2P at TSSs in WT cells may result from the enhanced recruitment of CDK9 upon UV‐C exposure. Interestingly, CDK9 ChIP‐seq demonstrated UV‐induced TSS‐proximal CDK9 enrichment in WT cells, which was substantially diminished in *ZFAS1*‐depleted cells (Figure S10I,J, Supporting Information). This observation aligns with the compromised RNAPII‐Ser2P acquisition at TSSs observed in *ZFAS1*‐deficient cells upon DNA damage.

**Figure 5:**
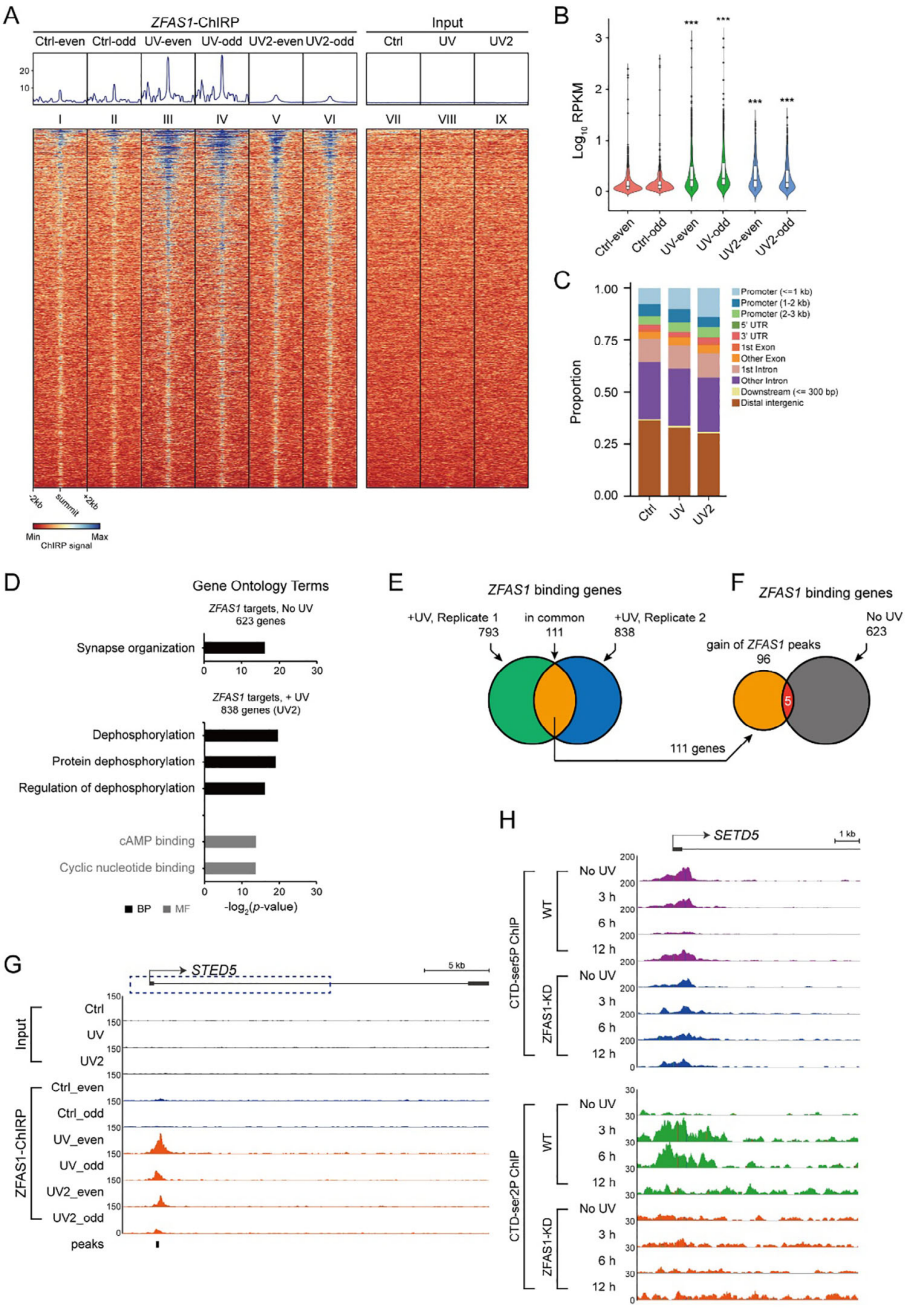
DNA damage induced chromatin binding of *ZFAS1*. A) Heatmap of *ZFAS1* ChIRP‐seq signal in filtered peak regions (true peaks). Each row is a 4 kb genomic window centered on a *ZFAS1* ChIRP peak in control and UV‐irradiated cells; the peaks are aligned for all the *ZFAS1* bound sites identified. Color bar indicates the intensity of ChIRP‐seq reads. B) Violin plots showing genome‐wide distribution (normalized by RPKM) of *ZFAS1* ChIRP‐seq “true peak” signal in the control and UV‐irradiated MRC5_VA cells. Two‐sided Wilcoxon rank test (*p* < 2.2e‐16) shows significant increase in chromatin occupancy for *ZFAS1* in response to UV‐C treatment for the two biological replicates. C) Distribution of *ZFAS1* binding sites based on the filtered peaks (true peaks) at the defined genomic regions. D) The significant Gene Ontology (GO) enrichments of the *ZFAS1* target genes in the wild type and UV‐irradiated cells (UV2 replicate was presented). BP, biological process; MF, molecular function. E) Venn diagram of *ZFAS1* ChIRP‐seq showing the number of genes with *ZFAS1* genomic occupancy upon UV‐C irradiation in MRC5_VA cells. *ZFAS1* target gene numbers from the two independent ChIRP‐seq replicates and their overlapping targets are presented. F) Venn diagram showing the number of *ZFAS1* target genes upon UV‐C irradiation in common between the two replicates that were not bound by *ZFAS1* in normal cells (termed as *ZFAS1*‐gain). G) *ZFAS1* occupancy at the *SETD5* loci from *ZFAS1* ChIRP‐seq analysis. H) RNAPIIo (*top*: CTD‐ser5P; *bottom*: CTD‐ser2P) distribution changes around the *SETD5* promoter region after UV‐C irradiation in the wild‐type and the *ZFAS1*‐depleted cell lines.

RT‐PCR analysis of nuclear and cytoplasmic fractions suggested that *ZFAS1* knockdown in MRC5_VA cells reduced its expression in both cellular compartments, implying that both cytoplasmic and nuclear *ZFAS1* might function during DDR (Figure S11A, Supporting Information). To further determine the molecular mechanisms by which *ZFAS1* manipulates RNAPIIo dynamics in response to DNA damage, chromatin isolation by RNA purification (ChIRP) was applied to map *ZFAS1* chromatin occupancy before and 3 h after UV‐C irradiation. *ZFAS1* RNA was specifically retrieved with “even” and “odd” pools of biotinylated capture probes, along with associated DNA (Figure S11B,C, Supporting Information). Notably, UV‐C irradiation induced robust changes in *ZFAS1* genomic distribution, with increased occupancy over gene bodies (Figure S11D,E, Supporting Information), suggesting a potential role for *ZFAS1* in modulating RNAPII traveling upon UV‐C irradiation. Using the previously described pipeline,^[^ ^43^ ^]^ we identified 5108 *ZFAS1* raw peaks in undisturbed cells, compared to 7319 (+UV) and 5938 (+UV2) raw peaks in irradiated cells across two biological replicates (Table S8, Supporting Information). After stringent filtering, 665 high‐confidence *ZFAS1* binding sites (“true peaks”) were detected in control cells, versus 823 and 874 true peaks in UV‐irradiated replicates (Table S9, Supporting Information). To compare *ZFAS1* binding across samples, read intensities of control‐even true peaks were ranked and found to correlate strongly control‐odd peaks (**Figure** **5**A, panels I‐II). When comparing UV‐treated samples to controls, *ZFAS1* occupancy patterns were similar but globally elevated (Figure 5, panels III‐VI; quantified in 5B). Consistent with this, UV irradiation increased raw peak counts (Figure S12A,B, Supporting Information). *ZFAS1* binding sites were enriched in genic regions, particularly promoters and introns (Figure 5C). Intriguingly, *ZFAS1* prominently localizes at the TSSs (almost exclusively increased at the region of promoter ≤1 kb) in the UV‐irradiated cells (Figure 5C), strongly indicating a role for *ZFAS1* in the regulation of transcription initiation/preinitiation during the post‐UV period. *ZFAS1* peaks were typically focal (<500 bp; Figure S12C,D, Supporting Information), resembling transcription factor binding patterns. Genes bound by *ZFAS1* after UV‐C treatment showed significant enrichment for dephosphorylation‐related GO terms (Figure 5D; Table S10, Supporting Information). When the most stringent thresholds were applied, among 111 common UV‐induced *ZFAS1*‐bound genes identified in both replicates (Figure 5E), only five overlapped with control‐bound genes (Figure 5F). Furthermore, these “*ZFAS1‐gain*” genes exhibited binding sites distributed across promoters, intragenic, and intergenic regions (Figure S12E, Supporting Information). Intriguingly, *ZFAS1* depletion disrupted RNAPIIo spatiotemporal dynamics at these loci post‐UV (Figure 5G,H; Figure S13A–D, Supporting Information). Motif analysis of UV‐induced *ZFAS1* binding sites revealed similarity to KLF5, p53, Pax7, and RarA transcription factor motifs (Figure S13E, Supporting Information), suggesting potential collaborative regulation.

Although only ≈7% of genes with dynamic RNAPIIo changes were *ZFAS1*‐bound (Figure S13F, red bars, Supporting Information), >58% of *ZFAS1*‐targeted genes showed early CTD‐Ser2P increase post‐UV (Figure S13F, blue bars, Supporting Information). Critically, most *ZFAS1*‐bound genes lost RNAPIIo redistribution upon *ZFAS1* knockdown (Figure S13F, white bars, Supporting Information). Finally, we assessed the potential correlation between *ZFAS1* binding events and DNA lesion repair. It has been found recently that the human fibroblast genome contains the hyper‐UV‐sensitive regions, most of which reside within genes.^[^ ^44^ ^]^ The result showed that *ZFAS1*‐bound genes did not preferentially overlap with CPD hyper‐hotspots (Figure S13G, Supporting Information). Taken together, these results indicate that UV‐induced genome‐wide recruitment of *ZFAS1* is not mechanistically linked to DNA damage recognition, but instead serves as a chromatin bookmark to license transcriptional reorganization upon DNA damage.

We next performed ChIRP assays using biotinylated *ZFAS1* followed by mass spectrometry (ChIRP‐MS) in MRC5_VA cells to identify potential *ZFAS1*‐interactors. The results demonstrated that *ZFAS1* interacts with multiple proteins both in the absence and presence of UV‐C exposure (Figure S14A, Supporting Information). Gene ontology analysis revealed significant enrichment of nucleosome‐related categories in the dataset (Figure S14B, Supporting Information). To refine candidate interactors, we conducted RNA pull‐down assays with biotinylated *ZFAS1* combined with mass spectrometry. Only six proteins, including the histone H3.3 variant, were identified by both methods (Figure S14C, Supporting Information). Immunoprecipitation of endogenous H3.3 showed substantial enrichment of *ZFAS1* upon UV damage (Figure S14D, Supporting Information). Notably, the robust H3.3 expression observed as early as 3 h after UV exposure was interfered in *ZFAS1*‐depleted cells (Figure S14E, Supporting Information). To investigate whether H3.3 enrichment correlates with enhanced *ZFAS1* binding during DDR, we mapped H3.3 distribution 3 h post‐irradiation. Elevated H3.3 levels were detected around TSSs following UV‐C exposure (Figure S14F,G, Supporting Information). Strikingly, among the 2509 genes showing UV‐induced H3.3 accumulation, 186 were *ZFAS1* targets exhibiting statistically significantly enrichment in response to DNA damage (Figure S14H, Table S11, Supporting Information). Importantly, H3.3 overexpression rescued DDR defects in *ZFAS1*‐depleted cells (Figure S14I,J, Supporting Information). It has been intensely studied that transcriptional control after DNA damage requires chromatin bookmarking by newly synthesized H3.3 histones and the HIRA chaperone complex to enable transcription resumption after genotoxic stress.^[^ ^45^ ^]^ Our findings illustrated that inhibition of H3.3 expression can impair DDR functionally in a manner similar to *ZFAS1* knockdown, suggesting that *ZFAS1* may facilitate H3.3 involving seemingly minor changes in chromatin resetting to confer on otherwise profound impact on cell fate in DNA damage response.

### 2.7 Germline Disruption of Mouse *Zfas1* Impairs DDR

Mouse *Zfas1* transcripts were detected in seven major mouse tissues examined (except the pancreas; see Figure S15A, Supporting Information). *Zfas1* showed high embryonic‐stage expression in tissues such as the liver and kidney, whereas postnatal expression was prominent in the brain and spleen (Figure S15A, Supporting Information). RACE confirmed the full‐length identity of mouse *Zfas1* (Figure S15B, Supporting Information). Overexpression of *Zfas1* in mouse fibroblasts promoted DNA lesion repair and enhanced cell survival after UV‐C irradiation (Figure S15C,D, Supporting Information).

**Figure 6:**
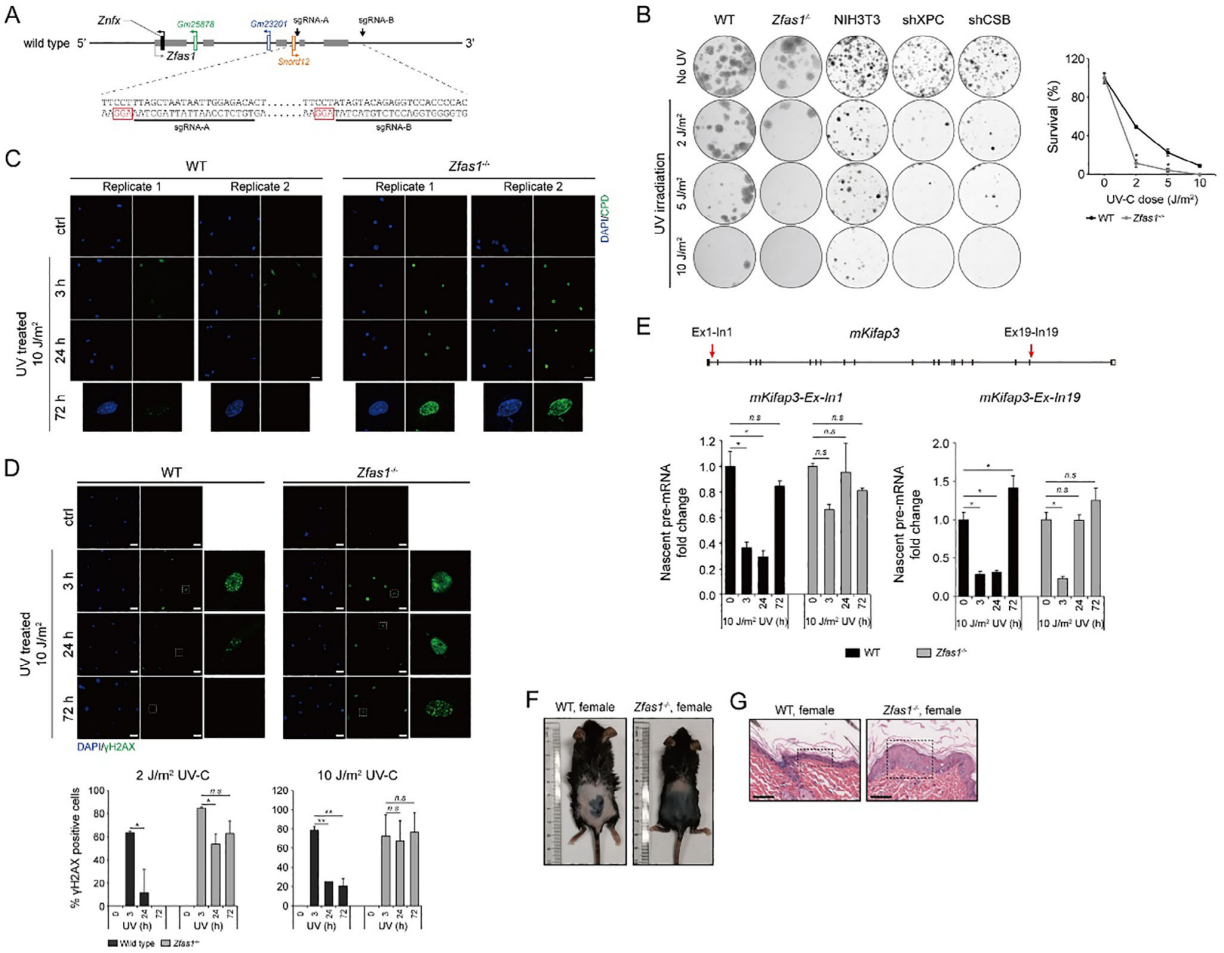
*Zfas1*‐deficient mice are defective in NER. A) Schematic representation of the mouse *Zfas1* gene locus and sgRNAs sequences. *Zfas1* exons are indicated in grey solid box. The sgRNA‐targeting sequences are underlined, with the *PAM* sequences highlighted in red. B) *left*: representative images of clonogenic survival assays of *Zfas1* ^‐/‐^ mouse fibroblasts and WT controls under the indicated UV dose. *right*: percentage of surviving cells (logarithmic scale) plotted against UV‐C dose. Error bars indicate the standard error of the mean from three independent experiments. *p < 0.05 compared to the WT mice (Student's *t*‐Test). NIH3T3 cells lacking either CSB or XPC were treated as positive controls. C) Representative images of primary cells showing repair of CPDs using a specific antibody to CPDs (green signal) for the WT and *Zfas1^‐/‐^* mice. DAPI‐stained nuclei in blue. Scale bar = 50 µm. D) *top*: representative images of primary fibroblast cells showing nuclear γH2A.X foci using a specific antibody to H2A.X phosphorylation (green signal) for the WT and *Zfas1^‐/‐^* mice. Nuclear DNA was counterstained with DAPI in blue. Scale bar = 50 µm. *bottom*: quantification of percent (%) γH2A.X‐positive (≥ 5 foci/nucleus) nuclei for each genotype, treatment and time point. A minimum of 500 cells per cell line per condition were analyzed. Each data point is presented as the means ± SD, *n* = 3. *p < 0.05; **p < 0.01 (Student's *t*‐Test). E) Nascent mRNA production in two distinct regions of the mouse *Kifap3* gene following UV‐C damage in the WT and *Zfas1^‐/‐^* fibroblasts. Means ± SD are shown from three independent experiments. *p < 0.05 (Student's *t*‐Test). F) Representative images of UV‐induced skin burn of *Zfas1^‐/‐^* mice. Shaven animals were exposed to UV‐B (500 J m^−2^/day) for 4 consecutive days, photographs were taken on the 7th day after the first exposure. Sample size for each genotype: *n* = 3 females. G) Hematoxylin and eosin staining (HE) of the skin sections of shaven *Zfas1^‐/‐^* and WT mice after exposure to UV‐B (500 J m^−2^/day) for 4 consecutive days. The wild‐type skin appears normal, the *Zfas1^‐/‐^* skin shows hyperplasia of epidermis (indicated by dashed boxes). Scale bar = 50 µm.

To characterize *Zfas1*’s role in DDR, we generated *Zfas1* knockout (KO) C57BL/6 mice using CRISPR/Cas9 (**Figure** **6**A). The targeting construct was designed to i) disrupt *Zfas1* expression and ii) avoid interference with the endogenous antisense transcript *Znfx1* (Figure S16A, Supporting Information). Mice were born at expected Mendelian inheritance ratios, indicating no embryonic or neonatal lethality. *Zfas1* KO mice develop normally, with body weights indistinguishable from heterozygous and wild‐type littermates (Figure S16B, Supporting Information). However, young (2‐month‐old) *Zfas1* KO mice displayed reduced kidney size (Figure S16C, Supporting Information). Histological analysis revealed glomerular lesions, including lobulation and abnormal mesangial cell proliferation (Figure S16D, Supporting Information), though these defects did not progress to renal failure.

Next, to confirm that targeted disruption of the mouse *Zfas1*, like human *ZFAS1*, resulted in defective NER, primary lung fibroblasts from *Zfas1* KO mice were examined. *Zfas1^‐/‐^* fibroblasts exhibited UV‐sensitivity in survival assays (Figure 6B) and impaired repair of UV‐induced DNA lesions (Figure 6C; Figure S16F, Supporting Information). Reduced UDS confirmed compromised GG‐NER in KO cells (Figure S16E, Supporting Information). Consistent with human *ZFAS1* depletion, *Zfas1^‐/‐^* fibroblasts displayed prolonged γH2A.X signaling, indicative of unresolved DSBs 24 h post‐UV irradiation (Figure 6D). Furthermore, DNA damage‐triggered reduced RNA synthesis was sustained for at least 24 h in the WT cells prior to restoration. In striking contrast, normal transcription levels were almost fully recovered within 24 h after UV‐C exposure in *Zfas1^‐/‐^* fibroblasts (Figure 6E). The photosensitivity of *Zfas1^‐/‐^* mice was also tested by exposing the shaven dorsal skin of wild‐type and homozygous mutant littermates to UV‐B light at the dose of 500 J m^−2^/day for 4 consecutive days. Compared to wild‐type mice, *Zfas1^‐/‐^* mice developed severe UV burns within a week (Figure 6F). Histological analysis of skin sections of *Zfas1^‐/‐^* mice revealed epidermal hyperplasia, which was not observed in the UV‐exposed skin of WT mice (Figure 6G). Taken together, these data demonstrate that *Zfas1* deficiency phenocopies human *ZFAS1* loss, impairing NER by disrupting transcription shutdown and recovery dynamics during DDR.

## 3 Discussion

Although the genome‐wide shutdown of transcription after UV‐C irradiation in human cells has been known for a considerable time,^[^ ^37^ ^]^ the precise mechanisms and functions underlying this phenomenon remain poorly understood. In this study, we identified the lncRNA *ZFAS1* as a critical component of the UV‐induced DNA damage response, which regulates the widespread UV‐triggered processing and distribution of RNAPIIo on chromatin. This regulatory mechanism is essential for both NER and cell survival following genotoxic stress.

### 3.1 Transcription Regulation after DNA Damage: Difference in lncRNAs Versus mRNAs

A cell's response to DNA damage depends on its precise cell‐cycle position, utilizing either an all‐or‐none checkpoint mechanism during G1 and G2 phases or a graded slowdown model in S phase, ultimately leading to heterogeneous cell fate outcomes.^[^ ^4c^ ^]^ In this study, we employed synchronously proliferating human fibroblast cells exposed to DNA damage at the same cell‐cycle stage to capture dynamic transcriptional reprogramming in response to genomic insults, with a specific focus on lncRNAs. Although the molecular mechanisms underlying major transcriptional events, including RNAP II stalling and degradation and transcriptional silencing and recovery, have recently been intensely implicated in DNA repair,^[^ ^12^, ^14^ ^]^ the functional consequences of this transcriptional coordination on repair efficiency and cellular survival remain largely unexplored. Through comprehensive time‐course RNA‐seq spanning the entire DNA damage response, we scrutinized potential differences between protein‐coding and lncRNA genes. Notably, protein‐coding genes show a stronger capacity to work in concert with cell‐cycle phase‐specific checkpoint dynamics (Figure 1). The early activation of DNA damage response genes reflects damage recognition and repair initiation, whereas induction of cell cycle genes at later time points marks precise resumption of proliferation (Figure S2 and S3, Supporting Information). In contrast, the tightly regulated stage‐specific transcriptional dynamics observed in protein‐coding genes are generally absent in lncRNAs (Figure 1).

Our prior work demonstrated that, unlike protein‐coding genes where shorter transcripts are preferentially recovered first, UV‐induced transcriptional repression of lncRNAs is alleviated earlier for longer transcripts.^[^ ^28^ ^]^ Consistent with reports that DNA damage‐inducible genes are predominantly short,^[^ ^46^ ^]^ we observed that UV‐induced lncRNAs during both early and late stages also tend to be shorter (Figure S4, Supporting Information). These findings unravel that lncRNAs may employ distinct regulatory mechanisms or specialized factors to initiate or restore transcription following DNA damage.

### 3.2 The Importance of Proper Transcription Shutdown and Recovery on TC‐NER

The cellular TC‐NER‐compromised phenotype of *ZFAS1*‐deficient cells was demonstrated by their sensitivity to UV‐C irradiation, delayed or inefficient removal of UV‐induced DNA lesions, and sustained expression of UV‐triggered H2AX phosphorylation (Figure 3). However, the impaired recovery of RNA synthesis – a general hallmark of TC‐NER disorders^[^ ^37^, ^47^ ^]^ – is absent in the *ZFAS1*‐deficient cells (Figure 4A). While it may appear counterintuitive that *ZFAS1* depletion results in both reduced cellular survival and faster transcription recovery in response to DNA damage, our data provide mechanistic insights. Strikingly, in *ZFAS1*‐depleted cells, we observed genome‐wide preservation (slight but nonsignificant increase) of RNAPII S5P levels at promoter‐proximal regions 3–6 h post‐UV irradiation. Reciprocally, there was no gain of RNAPII S2P immediately after UV exposure (Figure 4C–J). We, therefore, proposed that the defective global transcription during the early steps of DDR in *ZFAS1*‐depleted cells arises not from de novo recruitment of hypophosphorylated RNAPII molecules, but rather from the arrest of the already promoter‐bound elongating RNAPII. Consequently, ineffective transcription shutdown and aberrant cell cycle arrest fail to provide sufficient time for DNA repair, which may ultimately allow cells with damaged DNA to progress through the cycle. Whether *ZFAS1* directly regulates transcription shutdown by manipulating checkpoint factors requires further investigation.

Ultimate transcriptional recovery is crucial for cells to survive UV irradiation, a process defective in individuals with Cockayne syndrome.^[^ ^37^, ^38^ ^]^ Although several factors have been implicated in establishing the active transcriptional state upon DNA damage,^[^ ^20^, ^45^, ^48^ ^]^ the importance of transcription shutdown in mammalian cells remains incompletely understood. This is partly due to the precise events involved in RNAPII metabolism occurring within a few hours upon DNA damage being multitude and controversial. Earlier studies suggested that new transcription initiation is transiently inhibited after UV irradiation, coinciding with rapid release of promoter‐proximal RNAPII and global loss of its hypophosphorylated form.^[^ ^5^, ^40^, ^49^ ^]^ In contrast, recent work revealed that new RNAPII molecules are constantly recruited to PICs post‐UV, alongside increased chromatin accessibility and retention of active histone marks at TSSs. This phenomenon is underscored by stress‐dependent synthesis of initiation‐associated short RNAs.^[^ ^12^, ^28^ ^]^ Our findings demonstrate a severe reduction in RNAPII S5P at TSSs and a concomitant increase in RNAPII S2P downstream of TSSs in WT cells (Figure 4). We hypothesize that the loss of RNAPII S5P reflects the disengagement of a readily phosphorylated RNAPII molecules from DNA template after damage recognition, whereas increased RNAPII S2P may signify active transcription initiation to facilitate lesion scanning and repair. Notably, we do not exclude the possibility that elevated RNAPII S2P arises from stress‐induced modification of preloaded RNAPII S5P at active genes rather than de novo recruitment. In *ZFAS1*‐deficient cells, the rapid exchange of RNAPII phosphorylation states upon UV exposure is interrupted, leading to failed DNA repair and cell death. These data highlight the critical role of precise transcription shutdown and support a model in which the dynamic regulation of RNAPII pools governs the global transcriptional response to UV irradiation.^[^ ^14^ ^]^

### 3.3 *ZFAS1*‐Chromatin Interactions Guide DNA Damage‐Induced RNAPII Transcription

ChIRP‐seq revealed that *ZFAS1* occupancy sites in the genome are focal and numerous with an increasing tendency toward promoter‐proximal regions, peaking at TSSs in response to UV‐C irradiation (Figure 5A–C). Notably, a reciprocal gain in binding sites was observed following UV‐C exposure (Figure 5A,E). GO term analysis confirmed that genes with enhanced *ZFAS1* binding affinity are enriched in dephosphorylation process (Figure 5D). Among the individual genes targeted by *ZFAS1* upon DNA damage, two key candidates were identified: the tyrosine phosphatase EYA4, which specifically dephosphorylates Tyr‐142 of histone H2AX,^[^ ^50^ ^]^ and the adaptor protein NCK1, which mediates the catalytic activity of protein phosphatase I (PP1).^[^ ^51^ ^]^ While most DNA damage checkpoint pathways rely on active phosphorylation of their components, recent studies emphasize the critical roles of protein phosphatases as essential enzymes for re‐establishing the dephosphorylation status of multiple DDR targets during checkpoint arrest and cell cycle re‐entry.^[^ ^52^ ^]^ This regulatory involvement in fine‐tuning phosphorylation events may explain the aberrant cell cycle arrest observed in *ZFAS1*‐deficient cells upon UV exposure.

Compared to histone modifications, which broadly occupy genomic elements,^[^ ^53^ ^]^ the interspersed and gene‐selective *ZFAS1* occupancy pattern more closely resembles transcription factors. Like transcription factors, *ZFAS1* binds across gene bodies with focal peaks at TSS sites upon DNA damage, suggesting genome access through a highly discriminating mechanism. However, the specific regulatory elements defining *ZFAS1* target sites remain unclear. We identified a *ZFAS1*‐associated motif resembling transcription factor binding motifs but with a more degenerate pattern (Figure S13E, Supporting Information). This motif may either serve as a binding site for a protein that recruits *ZFAS1*, or indirectly configure a chromatin state (DNA structure) conducive to *ZFAS1* binding. Further investigation is now feasible due to the identification of the histone variant H3.3 as a candidate DNA‐binding partner. Through ChIRP‐MS and RNA pull‐down assays, we demonstrated UV‐dependent assembly of H3.3 with *ZFAS1* (Figure S14C,D, Supporting Information). The role of H3.3 incorporation in the activation and long‐term maintenance of gene expression patterns has been well documented due to its chromatin marking.^[^ ^54^ ^]^ Indeed, recent studies reveal its deposition at UV‐induced DNA damage sites prior to repair in transcribed regions, aligning with its proposed function in transcriptional recovery.^[^ ^45^, ^55^ ^]^ Here, we propose a novel nuclear role for H3.3 as part of a *ZFAS1* complex mediating transcriptional reprogramming in response to UV exposure. Genome‐wide occupancy mapping of H3.3 post‐DNA damage suggests that target gene selectivity is determined by *ZFAS1*, which may recruit H3.3 to chromatin or vice versa (Figure S14F–H, Supporting Information). Although the precise molecular mechanism of the *ZFAS1*‐H3.3 complex remains undefined, we uncovered a strong correlation between *ZFAS1* DNA binding events and RNAPIIo reorganization during DNA damage responses.

### 3.4 *ZFAS1* KO Mice Exhibit Impaired DDR: Implications for Developmental Disorders

We found that knockout of *Zfas1* in mice results in pronounced NER deficiency and kidney abnormalities. Strikingly, unlike patients with DDR deficiency, who typically develop devastating neurological dysfunction,^[^ ^56^ ^]^ *Zfas1*‐mediated DDR does not induce neuronal deficits. Interestingly, previous studies have reported that *ZFAS1* depletion causes kidney dysfunction.^[^ ^57^ ^]^ In support of this notion, accumulating evidence reveals that animal models with mutations in key DNA repair genes often exhibit tissue‐specific phenotypes. For example, in contrast to humans with Cockayne syndrome, TC‐NER‐deficient *Csa^‐/‐^* or *Csb^‐/‐^* mice fail to develop overt neurological phenotypes.^[^ ^47^, ^58^ ^]^ A similar discrepancy occurs in Fanconi anemia repair pathway‐deficient mice, which do not recapitulate FA pathology.^[^ ^59^ ^]^ Until now, we know surprisingly little about how the distinct DDR in different tissues and cell types is regulated, partly due to the lack of animal models. We propose that *Zfas1*‐deficient mice represent a valuable model for studying tissue‐specific DDR mechanisms.

Actually, renal disease is highly prevalent in Cockayne syndrome patients’ clinical presentations.^[^ ^60^ ^]^ Interestingly, recent work demonstrates that injured kidney cells can exert systemic effects beyond their local microenvironments, including on the central nervous system, highlighting the kidney's role in inter‐organ signaling.^[^ ^61^ ^]^ In this study, we show that *Zfas1* deficiency impedes DNA damage repair in mice, with marked physiological consequences in the kidney, suggesting that proximal tubule cells may be a key target of DNA damage‐induced transcriptional stress. However, whether *ZFAS1* deficiency directly or indirectly provokes kidney abnormalities requires further investigation.

Taken together, we mechanistically propose that *ZFAS1* exerts dual functions in DNA damage response: (1) facilitating the exchange and retention of distinct RNAPIIo forms to regulate transcription shutdown and recovery; (2) acting complementarily to (but not exclusively with) H3.3 to regulate initial DDR events, such as chromatin dynamics reorganization, thereby promoting DNA repair and transcriptional recovery.

## 4 Experimental Section

### Human Cell Lines and Culture Conditions

The following cell lines were used in this study: A549, human lung carcinoma cell line (ATCC, CCL‐185); IMR‐90, normal primary human lung fibroblast cells (ATCC, CCL‐186); MRC5_VA, SV40‐immortalized normal human fetal lung fibroblast cells.^[^ ^38b^ ^]^ All cells were maintained in high glucose DMEM medium (Thermo Fisher Scientific, 11 965 118) supplemented with 10% v/v fetal bovine serum (PAN‐Biotech, P30‐3302) and 5% penicillin/streptomycin (Thermo Fisher Scientific, 15 140 122) at 37 °C with 5% CO_2_ and routinely passaged 2–3 times a week. Cell lines were confirmed to be mycoplasma‐free.

### Animals

Animal experiments undertaken in this study were with approval of the Guangzhou Medical University Animal Care and Use Committee (Approval No. GY2023‐193). All mice were maintained under specific pathogen‐free conditions in individually ventilated cages at 19–23 °C with light from 07:00 to 19:00.

CRISPR/Cas9‐mediated genome engineering was conducted to generate a *Zfas1* ^‐/‐^ knockout C57BL/6 mouse. The targeting construct was designed to achieve two purposes: i) effectively disrupt the expression of *Zfas1* and ii) avoid interference with endogenous anti‐sense *Znfx1* and other neighbor genes. Cas9 vector and guide‐RNAs (target sequence: gRNA‐A, 5′‐GTGTCTCCAATTATTAGCTAAAGG‐3′; gRNA‐B, 5′‐GGGGTGGACCTCTGTACTATAGG‐3′) for mouse *Zfas1* were generated by in vitro transcription, followed by injection into fertilized eggs for knockout mouse production. The exon 4 and exon 5 were predicted to be deleted. The positive founders were genotyped by PCR and DNA sequencing analysis. The sequences of the primers used for genotyping were depicted in Table S12 (Supporting Information).

Acute effects in the skin of shaven *Zfas1^‐/‐^* mice following exposure to UV‐B at dose of 500 J m^−2^/day during 4 consecutive days were conducted by killing three mice per genotype 3 days after the last treatment. Skin samples were routinely processed (hematoxylin‐eosin staining) for histopathologic examination as described before.^[^ ^62^ ^]^

### Western Blotting

For whole cell extracts, cells pellets were lysed in NP‐40 lysis buffer [50 mM Tris‐HCl pH 7.5, 500 mM NaCl, 2 mM EDTA, 0.5% (v/v) NP‐40, 0.5 mM DTT, and Protease Inhibitor Cocktail (made in house)]. Protein samples were resolved by 10% or 15% SDS‐PAGE gels and transferred to PVDF membrane (GE healthcare Life Sciences, 10 600 023), followed by blocking in 5% (w/v) skimmed milk in TBST (50 mM Tris HCl pH 7.6, 150 mM NaCl, 0.1% (v/v) Tween 20) for 1 h at room temperature and incubated with primary antibody (in 5% (w/v) skimmed milk in TBST) overnight at 4 °C. Antibody against tubulin or GAPDH served as loading controls. Membranes were washed three times in TBST, followed by incubation with 1:1000 diluted HRP‐conjugated secondary antibodies in 5% (w/v) skimmed milk in TBST. After extensive washing with TBST, the proteins were visualized using Chemiluminescent Substrate ECL reagent (Thermo Fisher Scientific, 34 075). Antibodies used in the study are listed in Table S12 (Supporting Information).

Extraction of insoluble and soluble proteins from cells was performed by using kit (Sangon Biotech, C500071) according to the manufacturer's instructions.

### CPD, 6‐4PP and γH2A.X Immunofluorescence

Cells were maintained on coverslip and treated with UV‐C (10 J m^−2^) and recovered for the indicated period. After fixing in 4% paraformaldehyde for 15 min, cells were permeabilized in PBS‐T (PBS plus 0.3% (v/v) Triton X‐100), and the DNA was denatured in 2 M HCl for 5 min (for CPD immunofluorescence). Nonspecific binding was blocked in PBS‐T (PBS plus 0.3% (v/v) Triton X‐100) with 10% skimmed milk. The anti‐CPD antibody (Cosmo Bio, CAC‐NM‐DND‐001), anti‐64PP antibody (Cosmo Bio, CAC‐NM‐DND‐002), or anti‐γH2A.X antibody (Abcam, ab22551) was then applied in blocking solution (1:1000) and incubated at 4 °C overnight. Secondary anti‐mouse antibody conjugated to Alexa‐fluor‐488 was added to the coverslip for 1 h at room temperature. The coverslips were mounted onto slides using DAPI (Vector Laboratories, Inc. Peterborough, UK) and imaged as previously described.^[^ ^28^ ^]^

### Reverse Transcriptase Quantitative PCR

Total RNA was extracted using the RNeasy kit (QIAGEN, 74104) for nascent and mature RNA, following the instructions of the manufacturer including an on‐column DNase digestion (QIAGEN, 79254). Reverse transcription was performed using PrimeScript RT Reagent Kit with gDNA Eraser (Takara, RR047A). cDNA was amplified using iQ SYBR green Supermix (Bio‐Rad, 1708880) with 30 cycles of 15 s denaturation at 94 °C, 15 s annealing at 60 °C, and 20 s extensions at 72 °C. Primer sequences are listed in Table S12 (Supporting Information). Unless otherwise noted, reference gene (GAPDH) normalized RNA expression was compared between variable (ex. UV‐treated) and control samples using the Livak equation described before.^[^ ^63^ ^]^

### Cell Synchronization and Cell Cycle Profile Analysis

For all G1/S synchronization with thymidine, a double thymidine block was used as follows: cells were incubated for 14 h with 2 mM thymidine (Sigma‐Aldrich, T1895), released for 9 h in fresh growth medium supplemented with 24 µM deoxycytidine (Sigma‐Aldrich, D3897) after washing out the thymidine, and then blocked again with 2 mM thymidine for 14 h to arrest all cells at the beginning of S‐phase. Cell cycle arrest was subsequently released with two washes of thymidine‐free medium. After release, cells were treated with UV‐C irradiation (10 J m^−2^) and harvested at the indicated time intervals.

Cell cycle analysis was carried out by flow cytometry. Briefly, cells were seeded onto 10‐cm culture dishes, treated as indicated, collected, fixed, and stained with 25 µg/mL propidium iodide (Sigma‐Aldrich, 81 845) and 10 µg/mL RNAse A (Sigma‐Aldrich, RNASEA‐RO) in PBS for 30 mins at 37 °C, acquired on a FACSCalibur flow cytometer instrument and analyzed using FACStation and FlowJo software (BD Biosciences). A minimum of ten thousand events were analyzed for each sample.

### Cellular Fractionation

Cell nucleus and cytoplasm fraction isolation was performed as described before.^[^ ^28^ ^]^ Briefly, human fibroblast MRC5_VA cells grown to 80% confluence were scraped and collected by centrifugation (200 × *g*, 5 min, 4 °C). The cells pellets were resuspended in 0.1% NP40 ice‐cold PBS with protease inhibitor cocktail and Ribonucleoside Vanadyl Complex (10 mM) (NEB, S1402), after short centrifugation, the supernatant was collected as cytoplasmic fraction and the remainder with additional washing were considered as nuclear pellets.

### RNA‐seq Library Construction and Sequencing

G1/S‐phase‐synchronized MRC5_VA cells were either left untreated or treated with 10 J m^−2^ of UV‐C irradiation prior to release, followed by recovery for the indicated time periods from 3 to 96 hr. Total RNA was extracted and purified using RNeasy Mini Kit (QIAGEN, 74104) and analyzed on a 2100 Bioanalyzer (Agilent Technologies). All samples had an RIN (RNA Integrity Number) value of greater than 9.6. Subsequently, ribosome RNA was removed from the purified total RNA using Epicentre Ribo‐Zero rRNA Removal Kit (Epicentre, MRZH11124), and the remaining RNA was used for library preparation using TruSeq Stranded Total RNA Sample Prep Kit (Illumina, 20020597). Libraries were quantified fluorometrically using Qubit dsDNA HS Assay Kits (Thermo Fisher Scientific, Q32851) on a Qubit 2.0 Fluorometer (Thermo Fisher Scientific). All libraries (>2 nM µL^−1^) were sequenced on an Illumina NovaSeq 6000 (PE150 run) according to the manufacturer's instruction to a depth of 220–340 million reads on average.

The sequence data were deposited in the NCBI Gene Expression Omnibus (GEO), with BioProject accession number, GSE239617.

### Processing, Analysis, and Graphic Display of RNA‐seq Data

Raw reads were pre‐processed with sequence grooming tool FASTQC followed by sequence alignment using HISAT2.^[^ ^64^ ^]^ Transcript levels were quantified as fragments per kilobase of transcript per million mapped reads (FPKM) generated by TopHat/Cufflinks.^[^ ^65^ ^]^ SAMtools^[^ ^66^ ^]^ were used to convert sam files to bam files in order to make raw data visible on IGV (Integrative Genomics Viewer) or UCSC Genome Browser. Transcript levels were converted to the log‐space by taking the logarithm to the base 2. R studio (ggplot2 and gplots packages) was used to run custom R scripts to perform box plots, principal component analysis (PCA), dendrograms, and heatmaps. Samples were analyzed through DESeq2^[^ ^67^ ^]^ to obtain log2 fold change and its respective *p* value. Differentially expressed transcripts have been identified on these transformed values by using the criteria of log2 (Fold Change) ≥ 1 and padj < 0.05. Gene ontology analysis of DEGs (differentially expressed genes) was performed using cluster Profiler.^[^ ^68^ ^]^

In order to identify time‐dependent differentially expressed transcripts, the clustering of temporal profiles of gene expression for either mRNAs or lncRNAs was done using the R package, which performs soft clustering of genes based on their expression values using the k‐means algorithm. The differentially expressed transcripts that having a substantial change in either one of the time points (abs(logFC) > 0.75) were identified. Prior to generating the temporal profiles and heatmaps, scaling the expression values for each single gene (by Z‐score transformation of FPKM) was performed. Thus, in the standardized temporal profiles, the lowest expression value for each transcript in the different time points is 0 and the standard deviation is 1. Only those patterns with up‐ or down‐regulation at one specific time point were displayed in Figure 1 and Figure S2 (Supporting Information).

To predict the target genes of the differentially expressed lncRNAs, both co‐localization and co‐expression analyses were applied. Briefly, in the co‐localization analysis, the nearby protein‐coding genes within 100 kb upstream and downstream of the differentially expressed lncRNAs were identified. In the co‐expression analysis, the correlation between the expression of lncRNAs and mRNAs was calculated. Gene Ontology (GO) enrichment analyses of target genes of differentially expressed lncRNAs were implemented by the Goseq. The specific principle is to map the selected differentially expressed lncRNA‐targeted genes to each term of the GO database to calculate the number of genes contained in the entry. The hypergeometric test was then used to identify significantly enriched GO terms (with a corrected *p*‐value < 0.05) enriched by differentially expressed lncRNAs‐targeted genes.

### Identification of Novel lncRNAs from RNA‐seq

The known noncoding RNAs expressed in at least one sample were identified by blasting the transcripts against the NONCODE v6.0 database^[^ ^27^ ^]^ using the following selection criteria: identity > 0.9, coverage > 0.8, and E‐value < 10^5^. These transcripts were named as the ID number in the NONCODE v6.0 database.

Based on the features of lncRNA, a series of stringent screening conditions were established to identify novel lncRNAs from the RNA‐seq data: 1) Cuffmerge was used to merge the transcripts which were obtained by splicing and remove transcripts with uncertain directions and < 200 bp in length; 2) Cuffcompare was used to filter out transcripts that overlap with the database annotation exon region; 3) Cuffquant was applied to calculate the expression level of each transcript and transcripts with FPKM ≥ 0.2 were selected; 4) Four analysis tools, including CNCI (Coding‐Non‐Coding‐Index, v2), CPC (encoding potential calculator, 0.9‐r2), Pfam Scan (v1.3) and phyloCSF were used to predict the coding potential of the transcripts.

### *ZFAS1* Cloning and Sequencing Analysis (RACE) and Northern Blot Assay

3′ and 5′ RACE was performed using the SMARTer RACE 5′/3′ Kit (Takara, 634 858). RNA was extracted from either untreated or 10 J m^−2^ UV‐C exposed MRC5_VA cells or mouse NIH3T3 cells, and RACE was performed according to the standard manufacturer's protocol. Biotin Northern Blot assay was performed as described previously.^[^ ^69^ ^]^ Biotin‐labeled blots were detected with the Chemiluminescent Nuclei Acid Detection Module (ThermoFisher Scientific, 89880). Primers used for RACE assay and Biotin‐labeled probes used for Northern Blot were depicted in Table S12 (Supporting Information).

### Plasmid Construction

For construction of the *ZFAS1*‐FLAG plasmids, the predicted ORF of *ZFAS1*, full length of *ZFAS1*, and the CDS of *ACTB* gene were cloned into the Sgfl and Mlul sites of the eukaryotic expression vector pCMV6‐Entry with N‐terminal start codon ATG and C‐terminal Flag tag. For construction of the GFP‐*ZFAS1* plasmid, full length of *ZFAS1* was cloned into the EcoR1 and XhoI sites of the eukaryotic expression vector PCS2‐HA with C‐terminal GFP tag. SP1 luciferase reporter plasmid was constructed by inserting the core promoter region of *ZFAS1* with or without SP1 binding motif before the luciferase sequence of pGL3‐basic vector (Promega, E1751). Eukaryotic expression plasmid encoding SP1 was purchased from Sino Biological (HG12024‐CF). Primers used for the cloning were depicted in Table S12 (Supporting Information).

### RNA Fluorescence In Situ Hybridization (FISH)

To detect *ZFAS1* RNA, FISH analysis of MRC5_VA cells maintained on sterile glass coverslips was performed with a fluorescence in situ hybridization kit (RiboBio, C10910). Briefly, cells were rinsed in PBS and then fixed in 3.7% formaldehyde plus 10% acetic acid in PBS (pH 7.4) for 15 min at room temperature. Cells were subsequently permeabilized in PBS containing 0.2–0.5% Triton X‐100 and 5 mM vanadyl ribonucleoside complex (10 mM) (NEB, S1402) on ice for 5 min, washed in PBS 3 × 10 min and rinsed once in 2 × SSC buffer (Thermo Fisher Scientific, AM9770). Hybridization was carried out using oligodeoxynucleotide probes for *ZFAS1*, *U6*, and *18S* (RiboBio) in a moist chamber at 37 °C for 12–16 h while protected from light according to the protocol for adherent mammalian cell lines. The next day, cells were counterstained with DAPI. Images were acquired on a laser scanning confocal microscope (Leica, TCS SP8).

### Chromatin/RNA Immunoprecipitation Combined with Quantitative PCR (ChIP‐qPCR/RIP‐qPCR)

MRC5_VA cells were plated onto 15‐cm dishes at a density of 5 × 10^6^ cells per dish and treated with 10 J m^−2^ UV‐C exposure. After 3 hr of treatment, chromatin immunoprecipitation (ChIP) assays were carried out as described before.^[^ ^38b^ ^]^ For each ChIP assay, the following antibodies (2 µg) were used: RNA polymerase II (Abcam, ab26721), H3K4me3 (Abcam, ab8580), H3K27ac (Abcam, ab4729), SP1 (Santa Cruz, sc‐17824) or immunoglobulin G control (Abcam, ab172730). ChIP‐enriched DNA and input DNA were subjected to qRT‐PCR analysis with SYBR Green Supermix (Bio‐Rad, 1708880). Enrichment by ChIP assay on the specific genomic regions was assessed relative to the immunoglobulin G control.

For RIP assay, SUPERase‐In RNase inhibitor (1000 U/ml, Ambion, AM2694) and protease inhibitor were added into cell lysis buffer, and Ribonucleoside Vanadyl Complex (10 mM, NEB, S1402) was added into washing buffer. Antibodies specific to human H3.3 (Abcam, ab176840) was applied in the assay.

The primer sets are listed in Table S12 (Supporting Information).

### Luciferase Reporter Assay

HEK293T cells were transfected with a mixture of the indicated luciferase reporter plasmid containing SP1 recognition elements, pRL‐TK‐renilla plasmid as control, and the eukaryotic expression vector encoding SP1. 24–48 h after transfection, cells were collected and subjected to Luciferase assay according to the manufacturer's protocol (Yeasen Biotechnology, 11402ES60). Data were normalized for transfection efficiency by the division of firefly luciferase activity with that of renilla luciferase.

### Clonogenic Assay

Cultured MRC5_VA cells depleted with *ZFAS1* or overexpressing *ZFAS1* were treated with Trypsin/EDTA, counted, and seeded onto 6‐well plates at a low density (200–500 cells/well) for colony formation. Cells were incubated overnight followed by UV‐C irradiation under the dose of 2, 5, and 10 J m^−2^. Colonies were allowed to form over 14–21‐day period and were stained with crystal violet and counted.

### *ZFAS1* Knockdown and Overexpression

The target sequences of *ZFAS1*, shared by both short and long transcript variants, were 5′‐ GCAGACATCTACAACCTTC ‐3′ for shZFAS1‐A, 5′‐ ATGGATTTTGGAAGAGGGA ‐3′ for shZFAS1‐B, and 5′‐ GAGTTTAAAAGGCTGTGCC ‐3′ for shZFAS1‐C (shZFAS1‐C was used in the following functional assays). These sequences were cloned into lentiviral shRNA expression hCMV‐SMARTvector.

For overexpression assays in MRC5_VA cells, Lipofectamine 3000 (Invitrogen) was used according to the manufacturer's protocol. The following plasmids were used: pCMV6‐Entry‐human ZFAS1 and pCMV6‐Entry.

### Alkaline Comet Assay

Comet assay was performed according to ENZO comet assay kit (Enzo life sciences, ADI‐900‐166) protocol for alkaline comet assay of adherent cells. Samples were visualized using a confocal microscope (Leica, TCS SP8) with 40 × magnification. Comet parameters were analyzed automatically with image acquisition and analysis software package CaspLab.

### EdU Staining

Cells were cultured on coverslips and washed with PBS once, followed by irradiation with 10 J m^−2^ UV‐C. For local damage induction, cells were irradiated with a UV‐C dose of 100 J m^−2^ through a filter with holes of 5 µm in diameter (Millipore). After UV‐C treatment, cells were immediately incubated with serum‐free fresh medium supplemented with 10 µM EdU for 2 h. Cells were then washed with PBS once, followed by fixation with 3.7% formaldehyde for 15 min and permeabilization with PBS containing 0.5% Triton X‐100 for 20 min. After extensive washing with PBS, cells were blocked with 10% FBS in PBS for 30 min. Incorporated EdU was detected by fluorescent‐azide coupling reaction (Click‐iT EdU cell proliferation kit, Invitrogen, C10337). Photograms of the cells were captured with a confocal microscope (Leica, TCS SP8). Captured images were processed and analyzed with ImageJ Software. At least 200 non‐S‐phase cells were randomly selected per sample. Data points presented in the text are the averages of intensities.

### Ethynyl Uridine Staining

EU staining to detect newly synthesized RNA was performed according to the manufacturer's instructions (Click‐iT RNA imaging Kits, Invitrogen, C10329). The wild‐type or *ZFAS1*‐depleted MRC5_VA cells were exposed to 10 J m^−2^ UV‐C irradiation and incubated for the indicated period of time. Media was replaced with fresh media containing 0.75 mM 5′ Ethynyl Uridine (EU) and cells were incubated for another 2 h. EU‐containing media was then removed and cells were fixed in PBS buffered formaldehyde (3.7%) for 45 mins at room temperature, washed once with PBS, and followed by permeabilization with 0.5% Triton X‐100 diluted in PBS for 30 mins. Cells were washed once with PBS and then Alexa Fluor 488 Azide fluorophores were covalently attached to EU‐containing RNA by click reaction for 1 hr at room temperature. Cells were then counterstained and mounted with a mounting medium containing DAPI. Automated image acquisition of at least 5 fields per well was performed (Leica, TCS SP8).

### Chromatin Immunoprecipitation and NGS Sequencing (ChIP‐Seq)

RNAPII‐CTD ChIP‐Seq was performed under the following conditions: the wild‐type and *ZFAS1*‐depleted MRC5_VA cells were UV irradiated (10 J m^−2^ UV‐C), followed by incubation for designated time intervals (3 hr, 6 hr, and 12 hr). Cells were harvested by trypsin treatment and fixed in suspension with formaldehyde (1% final concentration) for 15 min at room temperature, with rotation. The crosslinking reaction was quenched with glycine (125 mM final concentration) for 5 min. Cells were then washed twice with ice‐cold 1 × PBS, suspended in 1 ml of ChIP cell lysis buffer (5 mM HEPES pH 8.0, 85 mM KCl, 0.5% NP‐40, and protease inhibitors) and incubated on ice for 5 min. Nuclei were pelleted by centrifugation at 3900 × g for 5 min at 4 °C, followed by suspension in ChIP nuclear lysis buffer (50 mM Tris‐HCl pH 8.1, 10 mM EDTA pH 8.0, 1% SDS, and protease inhibitors) and incubated for 5 min on ice. Nuclear lysate was sheared by using an ice‐water bath‐embedded Bioruptor sonication system at high power, 30 s on, 30 s off mode for 5–10 min. The size of the sheared DNA was checked by 2% agarose gel electrophoresis to be between 300–500 base pairs (bp). Sonicated chromatin was cleared by centrifugation at 20000 × g for 15 min at 4 °C. Before the immune‐precipitation, chromatin was diluted 1:5 with ChIP dilution buffer (0.01% SDS, 1.15 Triton X‐100, 1.2 mM EDTA pH 8.0, 16.7 mM Tris‐HCl pH 8.1, 167 mM NaCl, and protease inhibitors). 1 µg of anti‐RPB1 phospho‐Ser5‐CTD (RPB1‐Ser5, clone 3E8, Millipore, 04‐1572‐I), phospho‐Ser2‐CTD (RPB1‐Ser2, clone 3E10, Millipore, 04–1571), CDK9 (Abcam, ab239364), H3.3 (Abcam, ab176840), or control IgG (Invitrogen), was bound to 15 µL of Protein A/G agarose beads (Invitrogen) in 200 µL 5% BSA in 1 × PBS for 1 hr, before being washed twice with 500 µL of the same buffer. The sonicated chromatin was incubated with the antibody‐conjugated beads overnight at 4 °C with gentle rotation. Beads were washed twice with 1 ml of each of the following buffers: ChIP low salt buffer (0.1% SDS, 1% Triton X‐100, 2 mM EDTA, 20 mM Tris‐HCl pH 8.1, 150 mM NaCl); ChIP high salt buffer (0.1% SDS, 1% Triton X‐100, 2 mM EDTA, 20 mM Tris‐HCl pH 8.1, 500 mM NaCl); and ChIP LiCl buffer (10 mM Tris‐HCl pH 8.0, 250 mM LiCl, 1% NP‐40, 1% deoxycholic acid, and 1 mM EDTA). Beads were washed once with 1 ml of TE buffer (pH 8.0) and centrifuged for 5 min at 14000 × g before removing the buffer. Beads were finally suspended in 40 µL Elution buffer (50 mM Tris‐HCl pH 8.0, 10 mM EDTA, 1% SDS) and incubated at 65 °C for 15 min. The eluted ChIP material was incubated at 65 °C overnight to reverse the crosslinking with an additional 90 µL of 1% SDS in 1 × TE buffer and 1 µL of 10 mg/ml RNase A. In parallel, the input sample was also RNase treated and reverse crosslinked overnight at 65 °C. Proteinase K (100 µg) and Glycogen (20 µg) were added to the eluted ChIP material and incubated for 2 hr at 37 °C, and DNA was extracted by column purification (MinElute, QIAGEN, 28 006). DNA samples from three independent replicates were pooled together prior to sequencing. 1 ng of ChIPed DNA samples were submitted for further manipulation by standard ChIP‐Seq library preparation techniques (NEBNext Ultra DNA Library prep kit, NEB, #E7103) and sequenced under Illumina NovaSeq 6000 system, resulting in the production of 150 bp paired‐end reads.

Alignment of the ChIP‐seq reads was performed as follows: low‐quality sequence reads and adapters were filtered out by Trimmomatic (v3.36).^[^ ^70^ ^]^ The trimmed reads were then aligned to the human reference genome (hg38) with the Burrows‐Wheeler Aligner (BWA‐v0.7.12‐r1039).^[^ ^71^ ^]^ Duplicate reads were removed from the aligned reads by Biobambam2 (v2.0.72).^[^ ^72^ ^]^ The mapped reads were visualized with the Integrated Genome Viewer (IGV‐v2.3.90).^[^ ^73^ ^]^ Further analysis was conducted using Bioconductor.^[^ ^74^ ^]^

The sequence data were deposited in the NCBI Gene Expression Omnibus (GEO), with BioProject accession number, GSE239617.

### ChIP‐Seq TSS Profiles

From the aligned ChIP‐Seq reads, heatmaps and average density profiles were generated to draw spatial distributions of RPB1 along gene bodies under different conditions. Read densities at genomic regions (TSSs) of interest were extracted by SeqMINER 1.3.3.^[^ ^75^ ^]^ Heatmaps were generated directly in the software from matrixes of binned read densities for all considered individual items.

For determination of fold changes profiles, the number of reads per bin for a given sample was divided by the number of reads in the indicated control sample and expressed as Log_2_ FC.

### Promoter Escape Indexes

As described before,^[^ ^76^ ^]^ promoter escape indexes were calculated by taking the average coverage in rpm in the gene body (ranged from 101 bp to 2 kb downstream of TSS or 101 bp downstream of TSS to TTS for genes larger or smaller than 2 kb, respectively) divided by the average coverage on the promoter‐proximal region.

### RPB1 Peak Calling

Peaks were called against a rat IgG control using MACS v1.4.2.^[^ ^77^ ^]^ All the peaks are listed in Table S7 (Supporting Information).

### UV/DRB/GRO‐Seq

RNA polymerase II elongation rate experiments were done as described before: ≈6 × 10^6^ MRC5_VA cells were cultured in complete DMEM media containing 100 µM 5,6‐Dichlorobenzimidazole 1‐β‐D‐ribofuranoside (DRB) for 3.5 hr. DRB‐containing media was removed and cells were either left untreated or exposed to 10 J m^−2^ UV‐C irradiation. Cells were washed once with PBS and placed in fresh complete DMEM media without DRB. Cells were then incubated for 10, or 40 min. Transcription‐competent nuclei were prepared using the Nuclei Isolation Kit (Sigma‐Aldrich, NUC101‐1KT) by scraping cells in 10 mL of cold lysis buffer followed by a spin at 500 × *g* for 5 min at 4 °C, then resuspended in 400 µL cold storage buffer supplemented with protease inhibitors and Superase (Invitrogen, AM2694). Nuclear Run‐On reactions were carried out by addition of 400 µL Run‐On Buffer (10 mM Tris‐HCl pH 8.0, 5 mM MgCl2, 1 mM DTT, 300 mM KCl, 20 units of SUPERase, 1% Sarkosyl, 500 µM ATP, GTP, CTP and Br‐UTP (Sigma‐Aldrich, B7166) and incubation at 30 °C for 5 min. Run‐On reactions were stopped by the addition of 10 × DNaseI buffer (93 µL) and RNase Free DNase (40 µL) and incubation for 1.5 hr at 30 °C with shaking. Br‐UTP run‐on labeled RNA was isolated using anti‐Br‐UTP coupled agarose breads (Santa Cruz, sc‐32323 AC) at room temperature for 1 hr. Beads were washed once with low salt buffer (0.2 × SSPE, 1 Mm EDTA, 0.05% Tween), twice with high salt buffer (0.5 × SSPE, 1 Mm EDTA, 0.05% Tween, 150 mM NaCl) and twice with TE pH 8.0 + 0.05% Tween. RNA was eluted from beads in elution buffer (20 mM DTT, 300 mM NaCl, 5 mM Tris‐HCl pH 7.5, 1 mM EDTA, 0.1 mg/mL glycogen, 0.1% SDS) at room temperature. Eluates were acid phenol‐chloroform extracted and precipitated. The purified RNA was used for the preparation of strand‐specific RNA libraries using the TruSeq Stranded Total RNA LT Sample Prep Kit and sequenced on an Illumina Novaseq as single‐ended 51 bp reads.

### ChIRP‐seq/ChIRP‐MS

ChIRP was performed using biotinylated probes against *ZFAS1* according to previously described methods.^[^ ^43^ ^]^ See Table S12 (Supporting Information) for probe sequences. Independent even and odd probe pools were used to ensure *ZFAS1*‐specific retrieval. High‐throughput sequencing libraries were constructed from ChIRPed DNA according to ChIP‐seq protocol as described above and sequenced on Illumina NovaSeq 6000 system with a read length of 150 bp. Raw reads were uniquely mapped to the reference genome (hg38) using Bowtie.^[^ ^78^ ^]^

*ZFAS1* peak calling, motif finding, and GO term analysis were conducted as described previously.^[^ ^43^ ^]^ Briefly, peaks of each sample with even or odd probes were called using MACS against its corresponding input with p‐value cutoff 1 × 10^−5^. For each MACS peak, the peaks that share the same shape in the raw data from the two probe sets were filtered as “raw peaks”. For each MACS predicted peak, a window size of ± 2k bp around peak summit or peak width, whichever is smaller, is selected. Within this window, an average coverage of the combined lane and a Pearson correlation between the normalized per‐base coverage of the even and odd lanes were calculated. “Raw peaks” were further filtered based on the thresholds of average coverage (>1.5), fold enrichment against input lane (>2) and Pearson correlation (>0.3) to generate “True peaks”. The sequence data were deposited in the NCBI Gene Expression Omnibus (GEO), with BioProject accession number, GSE239617.

For ChIRP‐MS, protein elution, extraction protein sample preparation for MS were conducted as described before.^[^ ^79^ ^]^ See Table S12 (Supporting Information) for *ZFAS1* ChIRP probe design.

### RNA Pull‐Down Assay

*ZFAS1* fragments were amplified with primers containing T7 and SP6 promoter sequences and used as templates for the T7 RNA polymerase transcription kit with the Biotin RNA Labeling Mix (Roche, 11 685 597 910). In vitro transcribed RNA was treated with RNase‐free DNase I (Qiagen, 79 254) and purified with RNeasy Mini Kit (Qiagen, 74 104). 3 µg of biotinylated RNA in RNA structure buffer (10 mM Tris‐HCl pH 7.0, 0.1 M KCl, 10 mM MgCl_2_) was heated to 95 °C for 2 min, put on ice for 3 min, and then left at room temperature for 30 min to allow proper secondary structure formation. Folded RNA was then mixed with MRC5_VA chromatin or cytoplasm extract (containing ≈1 mg proteins) in 500 µL RIP buffer and then incubated at RT for 1 hr. 50 µL washed Streptavidin C1 dynabeads (Invitrogen, 65001) were added to each binding reaction and further incubated at RT for another 1 hr. Beads were washed briefly with RIP buffer five times and boiled in SDS buffer. Then the retrieved proteins were detected by Western blot or resolved in gradient gel electrophoresis followed by mass spectrometry (MS) identification.

### Isolation of Primary Mouse Lung Fibroblasts

Newborn mice were anesthetized with aether, the whole lung was removed. Approximately 1 cm^2^ fragment of the tissue was excised and placed immediately in sterile PBS to avoid drying. The tissue was cut into 1 mm pieces, transferred into a sterile 30 ml beaker containing 10 ml of DMEM/F12, 0.14 Wunsch units/ml liberase blendzyme 3, and 1 × antibiotic/antimycotic, incubated at 37 °C for 30–90 min with stirring. After digestion, the tissue fragments were rinsed 3 times with 10 ml of warm DMEM/F12 media containing 15% FBS and 1 × antibiotic/antimycotic, and then centrifuged at 524 × g. The pellet was resuspended in 10 ml of warm DMEM/F12 media with 15% FBS, 1 × antibiotic/antimycotic. The tissue pieces were broken down by pipetting with maximum force. Another 30 ml of DMEM/F12 media with 15% FBS, 1 × antibiotic/antimycotic was added and the solution was centrifuged twice at 524 × g to ensure removal of Liberase. The cell pellets were resuspended in 10 ml of DMEM/F12 media with 15% FBS, 1 × antibiotic/antimycotic transferred to a 10 cm tissue culture dish, and placed in a tissue culture incubator at 37 °C, 5% CO_2_.

### Immunohistochemistry

Mice were anesthetized with aether, and subjected to cardiac perfusion with saline, followed by a 10% formalin flush. Tissues (kidney or brain) were removed and sectioned into 3 mm slices before transfer into formalin. Tissues were fixed in 10% formalin for a minimum of 48 h at room temperature and then subjected to paraffin embedding schedule as follows: 70% Ethanol, two changes,1 h each; 80% Ethanol, one change, 1 h; 95% Ethanol, one change, 1 h; 100% Ethanol, three changes, 1.5 h each; Xylene, three changes, 1.5 h each; Paraffin wax (58 °C‐60 °C), two changes, 2 h each. After the paraffin wax cooled down and solidified, the paraffin blocks were trimmed and cut at 5 mm and placed in water bath at ≈40–45 °C. Sections were mounted into slides and air‐dried for 30 min, then baked in 45 °C oven overnight. Before deparaffinization, slides were baked in 65 °C oven for 2 h. Slides were placed in a rack and subjected to deparaffinization and dehydration in the following washes: Xylene, two changes, 3 min each; Xylene 1:1 with 100% ethanol, one change, 3 min; 100% ethanol, two changes, 3 min each; 95% ethanol, one change, 3 min; 70% ethanol, one change, 3 min; 50% ethanol, one change, 3 min. Slides were kept in the tap water until ready to perform antigen retrieval. Slides were placed to a boil in antigen retrieval buffer (10 mM Sodium Citrate, 0.05% Tween20, pH 6.0), then maintained at a sub‐boiling temperature for 10 min. Slides were cooled down in running tap water for 5 min. For H&E staining, slides were stained in hematoxylin for 3–5 min before antigen retrieval, and then washed in running tap water until sections blue for 5 min or less. Slides were differentiated in 1% acid alcohol (1% HCl in 70% alcohol) for 5 min and then washed in running tap water until the sections were again blue by dipping in an alkaline solution (ammonia water) followed by tap water wash. Slides were stained in 1% Eosin for 10 min and washed in tap water for 1–5 min. Slides were dehydrated through 95% alcohol, 2 changes of absolute alcohol, 5 min each, and cleared in 2 changes of Xylene, 5 min each. Finally, slides were mounted in mounting media. For cell‐specific immunohistochemical staining, slides were blocked in blocking solution (1 × PBS‐T containing 10% FBS, 1% BSA, and 0.3% Triton X‐100) for 1 h at room temperature after antigen retrieval. Slides were incubated in primary antibodies (diluted in 1 × PBS‐T containing 1% BSA) overnight at 4 °C. Slides were washed three times in PBS‐T and incubated in fluorophore‐conjugated secondary antibody diluted in PBS‐T, 1% BSA for 1 h at room temperature. Slides were washed three times in PBS‐T and mounted with mounting medium with DAPI.

### Quantification and Statistical Analysis

Raw data from RNA‐seq were log‐transformed to meet normality. All continuous variables were expressed as mean ± SD unless noted. A two‐tailed Student's *t*‐Test was used for analysis of statistical significance (comparisons between two groups), with a *P* < 0.05 considered significant. One‐way ANOVA with Tukey's post‐hoc correction (multi‐group comparisons, α = 0.05) was used. Each experiment had n ≥ 3 biological independent replicates. For mouse studies, we determined that a sample size of at least 3–6 animals per genotype per sex was required. Analyses were performed using GraphPad Prism 9.0.

## Conflict of Interest

The authors declare no conflict of interest.

## Author Contributions

J.L., Q.L., Z.F., J.L., and N.H. contributed equally to this work. Y.W. conceived the project. J.L., Q.L., Z.F., and J.L. conducted all experiments. N.H. performed most of the data analysis. X.Z., Z.H., and T.Z. conducted overexpression experiments. Z.W., and Y.X. coordinated the study. Y.W. wrote the manuscript, with input from all authors.

## Supporting information



Supporting Information

Supplemental Table S1

Supplemental Table S2

Supplemental Table S3

Supplemental Table S4

Supplemental Table S5

Supplemental Table S6

Supplemental Table S7

Supplemental Table S8

Supplemental Table S9

Supplemental Table S10

Supplemental Table S11

Supplemental Table S12

## Data Availability

The data that support the findings of this study are available from the corresponding author upon reasonable request.
