# Supplementary material for: IncRNA‐ZFAS1, an Emerging Gate‐Keeper in DNA Damage‐Dependent Transcriptional Regulation
Source: Adv Sci (Weinh). 2025 May 24;12(31):e12385. doi: 10.1002/advs.202412385 (PMC12376587; doi:10.1002/advs.202412385)

**Figure S1. Characterization of cellular response upon UV-C irradiation, Related to Figure 1**

**(A)** The changes in Cyclin A and Cyclin B expression through cell cycle progression. *top*: qPCR quantification of *CCNA* and *CCNB* mRNAs, relative to *GAPDH* and normalized to synchronized cells at the G1/S boundary. *bottom*: representative images and quantifications of western blot analysis of Cyclin A and Cyclin B proteins from three independent experiments. Error bars represent ± SD from three independent experiments. Student’s *t*-Test, **p* < 0.05, and ***p* < 0.01.

**(B)** Immunofluorescence staining of CPD dimers in synchronized MRC5_VA cells released for the indicated time intervals from the G1/S block after UV-C treatment (10 J/m^2^); nuclei were visualized by DAPI staining. Scale bar = 100 μm.

**(C)** Nascent mRNA production in two distinct regions of the human *KIFAP3* gene in synchronized MRC5_VA cells released from the G1/S block with or without UV-C exposure. Means ± SD are shown from three independent experiments. Student’s *t*-Test, **p* < 0.05, ***p* < 0.01.

**(D)** Pie charts showing the number of different types of transcripts detected in the study.

**(E)** PCA analysis of temporal transcriptomic data for mRNAs and lncRNAs respectively. pc, principal component.

**(F)** Correlation matrix showing the results of Pearson correlation analysis. Pearson correlation coefficient values are marked with different colors.

**Figure S2. Stage-specific protein-coding gene expression profiles of human fibroblasts with or without genotoxic stress, Related to Figure 1**

**(A)** Heatmap of transformed FPKM of temporal genes showing data from RNA-seq of non-irradiated MRC5_VA cells released from the G1/S block. Transcript-wise hierarchical clustering heatmap of differentially expressed genes showing segregation into six groups. Cluster 2 (*n* = 3,764) includes genes with increased expression at 3 h after release. Cluster 3 (*n* = 2,206) includes genes upregulated at 6 h after release. Plots show a representative expression profile for each indicated cluster. Enriched GO terms are shown to the right.

**(B)** Box plots illustrating relative expression of representative marker genes in the indicated clusters shown in (A).

**(C)** Heatmap of transformed FPKM of temporal genes showing data from RNA-seq of UV-irradiated MRC5_VA cells released from the G1/S block. Transcript-wise hierarchical clustering heatmap of differentially expressed genes showing segregation into seven groups. Cluster 2 (*n* = 725) includes genes with increased expression at 3 h after release. Cluster 6 (*n* = 1,382) includes genes upregulated at 72 h after release. Plots show a representative expression profile for each highlighted cluster. Enriched GO terms are shown to the right.

**Figure S3. Expression changes of cell cycle regulators across DDR, Related to Figure 1**

**(A)** Expression changes of two individual cyclin markers in MRC5_VA cells across the cell cycle with or without treatment of UV-C irradiation (10 J/m^2^). qPCR quantification of *CCNA1* and *CCNB1* mRNAs, relative to *GAPDH* and normalized to synchronized cells at the G1/S boundary. Error bars represent ± SD from three independent experiments.

**(B)** Expression changes of two individual spindle genes in MRC5_VA cells across the cell cycle with or without treatment of UV-C irradiation (10 J/m^2^). qPCR quantification of *AURKA* and *PLK1* mRNAs, relative to *GAPDH* and normalized to synchronized cells at the G1/S boundary. Error bars represent ± SD from three independent experiments.

**(C)** Expression changes of two individual transcription repressors in MRC5_VA cells across the cell cycle with or without treatment of UV-C irradiation (10 J/m^2^). qPCR quantification of *CDKN1A* and *KLF10* mRNAs, relative to *GAPDH* and normalized to synchronized cells at the G1/S boundary. Error bars represent ± SD from three independent experiments.

**(D)** Representative images and quantification of western blot analysis of the AURKA, PLK1, CDKN1A and KLF10 proteins from three independent experiments. Error bars represent ± SD from three independent experiments. Student’s *t*-Test, **p* < 0.05, and ***p* < 0.01.

**Figure S4. Features of DNA damage-induced lncRNAs, Related to Figure 1**

**(A)**-**(B)** Changes of two groups of representative lncRNAs across the cell cycle in the absence or presence of genotoxic stress. qPCR quantification of the indicated lncRNAs, relative to *GAPDH* and normalized to synchronized cells at the G1/S boundary. Error bars represent ± SD from three independent experiments.

**(C)** Exon number for all lncRNAs in the genome and the DNA damage-induced lncRNAs that are grouped into the indicated clusters.

**(D)** Distribution of CDS sizes for the DNA damage-induced lncRNAs grouped into the indicated clusters. Each bar represents the number of CDSs in each size class in increments of 80 bp. Where there are several CDS isoforms in one gene, they are all counted. CDS, coding sequence.

**(E)** Transcript length between all lncRNAs and the DNA damage-induced lncRNAs grouped into the indicated clusters.

**Figure S5. Identification of *ZFAS1* as a DNA damage-induced lncRNA, Related to Figure 2**

**(A)** Real-time PCR validations of *SNHG15*, *SLC25A34-AS1*, and *ZFAS1* in the G1/S-phase-synchronized MRC5_VA cells upon UV-C irradiation. The expression of each gene was normalized to house-keeping gene *GAPDH* and presented relative to non-irradiated samples. Error bars represent ± SD from three independent experiments.

**(B)** RNA-seq expression of three distinct *ZFAS1* isoforms in the G1/S-phase-synchronized MRC5_VA cells during the DNA damage-induced cell cycle progression.

**(C)** RNA-seq expression of the *ZNFX1* gene in the G1/S-phase-synchronized MRC5_VA cells during the DNA damage-induced cell cycle progression.

**(D)** Representative images and quantification of western blot analysis of the ZNFX1 protein from three independent experiments. Error bars represent ± SD from three independent experiments. Student’s *t*-Test, **p* < 0.05, and ***p* < 0.01.

**Figure S6. Molecular features of *ZFAS1*, Related to Figure 2**

**(A)** The evolutionary conservation of *ZFAS1* lncRNA over its entire length is depicted using the UCSC genome browser.

**(B)** Schematic diagram showing sequences of identified 5’ and 3’ ends of *ZFAS1* with RACE.

**(C)** Sequences identified by RACE for the two variants (long and short isoforms) of *ZFAS1*.

**(D)** *top*: biotin northern blot analysis of the two variants of *ZFAS1* following UV-C exposure. *bottom*: probes to identify these two variants were indicated.

**(E)** Expression profiles of *ZFAS1* across various human tissues, adapted from NONCODEV6.

**Figure S7. Coding capacity and expression dynamics of *ZFAS1* in response to DNA damage, Related to Figure 2**

**(A)** Protein-coding potential of *ZFAS1* predicted by PhyloCSF database, *ACTB* was presented as a positive control.

**(B)** Agarose gel electrophoresis of eukaryotic expression recombination plasmids of pCMV6-Entry-ZFAS1-FL, pCMV6-Entry-ZFAS1-pORF, and pCMV6-Entry-ACTB by double digestion of Sgfl and Mlul. The marker was DL5000 at lane 1.

**(C)** Western blot of insoluble and soluble proteins extracted from the MRC5_VA cells expressing either full-length *ZFAS1*-Flag, *ZFAS1*-pORF-Flag or *ACTB-*Flag control. H3K4me2 was used as a loading control for the insoluble protein.

**(D)** Exponentially growing MRC5_VA cells transfected with plasmids expressing either *ZFAS1* or *ACTB* control were treated with the proteasome inhibitor MG132 (10 μM) prior to protein extraction. Western blot analysis of whole cell extracts against FLAG antibody. GAPDH levels were shown as loading controls. RNAPII levels upon UV irradiation with or without MG132 treatment were shown.

**(E)** Cytoplasmic and chromatin extracts from MRC5_VA cells before and after UV-C treatment aligned to the human genome (hg38) and presented as coverage (normalized reads per million) as a function of chromosomal coordinate at the *ZFAS1* loci.

**(F)** qRT-PCR of *ZFAS1* in the unsynchronized MRC5_VA cells treated with the indicated doses of different DNA damaging agents. Error bars represent ± SD from three independent experiments. Student’s *t*-Test, **p* < 0.05.

**(G)** qRT-PCR of *ZFAS1* expression at the indicated time intervals after the DNA damage agent’s treatment in MRC5_VA cells. Error bars represent ± SD from three independent experiments. Student’s *t*-Test, **p* < 0.05.

**(H)** qRT-PCR of *ZFAS1* expression in the IMR-90 primary human fibroblasts and A549 lung cancer cells treated with 10 J/m^2^ UV-C. Error bars represent ± SD from three independent experiments. Student’s *t*-Test, **p* < 0.05.

**(I)** The chromatin accessibility in the regulatory regions of *ZFAS1*. The average ATAC-seq signal for the untreated and UV-irradiated MRC5_VA cells (at 3 h post-irradiation) was shown. A schematic representation of *ZFAS1* locus was diagramed at the top.

**(J)** Representative western Blot images (*left*) and quantification (*right*) of SP1 in the wild-type MRC5_VA cells post-UV irradiation. Values are means ± SD, n = 3 independent experiments.

**Figure S8. *ZFAS1* overexpression enhances cell viability after DNA damage, Related to Figure 3**

**(A)** qRT-PCR analysis of *ZFAS1* expression in MRC5_VA cells treated with three independent shRNA expression lentiviral plasmids targeting distinct regions of *ZFAS1*, showing averaged *GAPDH*-normalized data, relative to control cells (shCtrl). Each data point is presented as the means ± SD, n = 3. Student’s *t*-Test, **p* < 0.05, ***p* < 0.01.

**(B)** *left*: representative images of clonogenic survival assays of MRC5_VA cells overexpressing *ZFAS1* under the indicated UV-C doses. *right*: percentage of surviving cells (logarithmic scale) plotted against UV-C dose. Each data point is presented as the means ± SD, n = 3. **p* < 0.05 compared to the WT (empty vector) cells (Student’s *t*-Test).

**(C)** Short-term growth curve of MRC5_VA cells overexpressing *ZFAS1* exposed to 10 J/m^2^ UV-C irradiation. Untreated cells were used as controls. Each data point is presented as the means ± SD, n = 3. **p* < 0.05 compared to the WT cells (Student’s *t*-Test).

**(D)** Cell viability of WT, CS-B and *ZFAS1*-depleted cells after 3 days treatment with the indicated concentrations of potassium bromate. Error bars indicate standard error of the mean from three independent experiments. ***p* < 0.01 compared to the WT cells (Student’s *t*-Test).

**(E)** Cellular localization of green fluorescence protein (GFP)-tagged *ZFAS1*.

**(F)** Representative images of WT and *ZFAS1*-depleted cells showing repair of 6-4PPs using a specific antibody to 6-4PP (green signal). DAPI-stained nuclei in blue. Scale bar = 25 μm.

**(G)** Representative images of cells showing repair of CPDs at the indicated recovery time post UV irradiation using a specific antibody to CPDs (green signal) for the wild-type and MRC5_VA cells overexpressing *ZFAS1*. DAPI-stained nuclei in blue. Scale bar = 50 μm.

**(H)** *left*: representative images from comet assay of cells treated with 10 J/m^2^ UV-C and untreated controls. Scale bar = 100 μm. *right*: data from comet assay presented as box-whisker plots of the tail moment (A.U arbitrary units) of > 210 individual nuclei. ****p* < 0.0001 (Student’s *t*-Test). The horizontal black bars represent the median tail moment for each dataset.

**(I)** Bar graph representation of the flow cytometry profiles at the indicated time points after release from the G1/S block of control (wild-type) cells and *ZFAS1*-depleted cells without genotoxic stress. Each data point is presented as the means ± SD, n = 3. **p* < 0.05 compared to wild-type cells (Student’s *t*-Test).

**(J)** Representative images of clonogenic survival assays of the wild-type and *ZFAS1*-depleted IMR-90 human primary fibroblasts cells under the indicated UV-C doses.

**(K)** Representative images of clonogenic survival assays of the wild-type and *ZFAS1*-depleted A549 human cancer cells under the indicated UV-C doses.

**(L)** *left*: representative images of the EdU incorporation for the indicated cell lines treated with UV-C irradiation (10 J/m^2^). Scale bar = 50 μm. White arrows indicate non-S-phase cells with unscheduled DNA synthesis (UDS) occurring, red arrows indicate S-phase cells. *right*: Bars represent frequencies of the fluorescence levels in the indicated cell lines, with (orange) or without (blue) UV-C irradiation. Asterisks indicate the mean values of the nuclear fluorescence intensities, which correspond to the UDS levels. Δ represents UDS difference between irradiated and unirradiated samples. n = 50 non-S-phase cells from three independent biological experiments.

**(M)** Quantification of the TCR-UDS assay determined by EdU incorporation after local damage induction with UV-C (100 J/m^2^) in the indicated cell lines. Error bars represent the SEM obtained from at least 50 local damaged areas of the cells. ****p* < 0.001 compared to wild-type cells (Student’s *t*-Test).

**Figure S9. DNA damage-induced exchange of RNAPIIo forms is dependent on *ZFAS1*, Related to Figure 4**

**(A)** Representative images of the wild-type and *ZFAS1*-depleted MRC5_VA cells 3 h and 24 h after UV irradiation. CS1AN cells (with mutations on *CSB* gene) were used as negative control for RRS assay, while *XPC*-depleted MRC5 cells (knocked down by siRNA) were used as TC-NER proficient cells. Nascent EU-labeled RNA transcripts shown in green and DAPI-stained nuclei in blue. Scale bar = 100 μm.

**(B)** Nascent mRNA production in different regions of the human *KIFAP3* gene after release from DRB-inhibition in the wild-type and *ZFAS1*-depleted MRC5_VA cells.

**(C)** Profiles of nascent *PPP1R12A* and *CTNNBL1* RNA reads after DRB-release with or without UV-C irradiation in the WT and *ZFAS1*-depleted MRC5_VA cells from GRO-seq. Arrows indicate the approximal transcription wave-fronts.

**(D)** Computationally determined wave fronts during transcription restart, 10 mins or 40 mins after DRB removal with or without UV-C irradiation. Data are box and whisker (min to max), with median indicated. The median wave front for the 40 min time points in the WT cells is indicated by the black dashed line.

**(E)** Western blot of RNAPII-specific ChIPs using antibodies directly against the factors indicated on the left in WT and *ZFAS1*-depleted cell lines upon UV-C irradiation.

**(F)** Heatmaps illustrating the distribution of RNAPII-Ser5P reads without UV-C irradiation, as aligned at individual (rows) promoter-proximal regions (-250 bp to + 2 kb relative to TSSs) for the WT and *ZFAS1*-depleted cells.

**(G)** *top*: distributions of RNAPII-Ser5P read densities around TSSs and flanking regions (-250 bp to + 2kb relative to TSSs) without UV-C irradiation. *bottom*: the same as in (C) but categorized by gene expression status (active and inactive), as defined in previous report[5a].

**(H)** Heatmaps illustrating the Log_2_ fold change (FC) of RNAPII-Ser5P comparing reads density between UV irradiation (UV+) at the indicated time points and steady-state (UV-) (FC = read density UV+/read density UV-), as aligned at individual regions (-250 bp to +2 kb relative to TSSs).

**(I)** Same as in (H), but showing the Log_2_ FC of RNAPII-Ser2P.

**(J)** Heatmaps from ChIP-seq data showing RNAPII-Ser5P binding events before (No UV) and at 3h, 6h, and 12 h post UV (+ UV) for genomic regions 5 kb around peaks in the MRC5_VA cells overexpressing *ZFAS1*. Data aligned to untreated RNAPII-Ser5P peak position.

**(K)** Same as in (J), but showing RNAPII-Ser2P binding events.

**(L)** Representative western Blot images (*top*) and quantification (*bottom*) of RNAPII CTD-Ser5P and CTD-Ser2P levels from the chromatin fraction of the WT and *ZFAS1*-depleted MRC5_VA cells at 1 h or 3 h post-UV irradiation. Values are means ± SD, n = 3 independent experiments. **p* < 0.05 compared to unperturbed cells (Student’s *t*-Test).

**Figure S10. *ZFAS1* is not required for global transcription of mature mRNAs after UV stress, Related to Figure 5**

**(A)** IGV snapshot showing tracks for *ZFAS1* and its nearby coding gene *ZNFX1* from RNA-seq of the wild-type and *ZFAS1*-depleted MRC5_VA cells treated with 10 J/m^2^ UV-C irradiation. The *ZFAS1* and *ZNFX1* loci were diagramed on the top.

**(B)** qRT-PCR analysis showing the expression levels of *ZFAS1* and *ZNFX1* in response to UV-C exposure in the WT and *ZFAS1*-depleted MRC5_VA cells. Each data bar is presented as the means ± SD from three independent experiments. ***p* < 0.01 compared to the WT cells (Student’s *t*-Test).

**(C)** Principal component analysis (PCA) in the wild-type and two independent *ZFAS1*-depleted cell lines (KD1, KD2) upon UV-C irradiation from RNA-seq data.

**(D)** RNA-seq analysis of differentially expressed genes (DEGs) in response to 10 J/m^2^ UV-C irradiation in the wild-type and two independent *ZFAS1*-depleted cell lines. DEGs were considered at *P* value < 0.05 and Log_2_ Fold change > 1.

**(E)** The top 5 enriched GO terms for up-regulated genes in response to UV-C irradiation (UV_24h/untreated) in the wild-type cells and the two individual *ZFAS1*-depleted cell lines.

**(F)** Relative expression levels of the two selected DNA damage response genes upon UV-C exposure in the WT and *ZFAS1*-depleted cell lines.

**(G)** Venn diagram showing the number of ATF3-dependent genes in the wild-type and *ZFAS1*-depleted MRC5_VA cells that were downregulated 3 hr upon UV-C irradiation.

**(H)** qPCR analysis showing the activation and degradation of ATF3 mRNA in response to UV-C exposure in the WT and *ZFAS1*-depleted cells. Each data bar is presented as the means ± SD from three independent experiments. **p* < 0.05 compared to the undisturbed cells (Student’s *t*-Test).

**(I)** Boxplots summarizing quantifications of CDK9 ChIP-seq reads shown for all TSSs genome-wide in the WT and *ZFAS1*-depleted MRC5_VA cells upon UV-C exposure. Boxplots show the 25th-75th percentiles, and error bars represent data range to the larger/smaller value. *p* values were calculated with Mann-Whitney *U* test and were correlated by the Benjamini-Hochberg method. ***p* < 2.22e-16.

**(J)** Heatmaps from ChIP-seq data showing CDK9 binding events for genomic regions 5 kb around peaks in the WT and *ZFAS1*-depleted MRC5_VA cell lines. Data aligned to non-irradiated CDK9 peak position.

**Figure S11. Genome occupancy of *ZFAS1* analyzed by ChIRP-seq in MRC5_VA cells prior to and after DNA damage induction, Related to Figure 5**

**(A)** RT-PCR detection of *ZFAS1* in the cytoplasmic and nuclear fractions of the WT and *ZFAS1*-knockdown MRC5_VA cells. *GAPDH* and *U6* serve as cytoplasmic and nuclear localization control, respectively. Each data bar is presented as the means ± SD from three independent experiments. **p* < 0.05, ***p* < 0.01 compared to the WT cells (Student’s *t*-Test).

**(B)** *left*: agarose gel electrophoresis showing that chromatin is sheared into the size range of 100-500 bp by sonication. *right*: qRT-PCR analysis of RNA retrieval with the indicated ChIRP probe pools (*LacZ* as a negative control) from the wild-type MRC5_VA cells.

**(C)** Tracks of the *ZFAS1* binding profiles at the *ZFAS1* locus and its nearby gene loci from the ChIRP-seq data. The genomic coordinates of each gene are presented on the top.

**(D)** Genome browser screenshots representing *ZFAS1* ChIRP-seq reads count before (No UV) and after damage induction (UV-3 h) at the indicated genomic loci. TSS sites are illustrated as short green bars below the genomic coordinates.

**(E)** Average *ZFAS1* ChIRP-seq profiles across all genes on the genome (hg38), before and after UV-C irradiation as indicated.

**Figure S12. Increased genomic occupancy of *ZFAS1* after UV stress, Related to Figure 5**

**(A)** Heatmap of *ZFAS1* ChIRP-seq signal in overlapping peak regions (raw peaks). Each row is a 4 kb genomic window centered on a *ZFAS1* ChIRP peak in control and UV-irradiated cells; the peaks are aligned for all the *ZFAS1* bound sites identified. Color bar indicates the number of ChIRP-seq reads.

**(B)** Violin plots showing genome-wide distribution of *ZFAS1* ChIRP-seq raw peak (overlapping peaks) signals in the control and UV-irradiated MRC5_VA cells lines. Two-sided Wilcoxon rank test (*p* < 2.2e-16) shows significant increase in chromatin occupancy for *ZFAS1* in response to UV-C treatment.

**(C)** Distribution of narrow peaks with distinct peak width.

**(D)** Representative images of IGV screenshot of the *ZFAS1* ChIRP-seq read densities and peaks called by MACS in the UV-irradiated cells. The genomic coordinates of each gene are presented on the top.

**(E)** Proportion of *ZFAS1*-gain genes in intergenic, intragenic, and promoter regions of all *ZFAS1*-occupancy sites upon UV irradiation.

**Figure S13. *ZFAS1* mediates the recruitment of RNAPIIo molecules on its target genes, Related to Figure 5**

**(A)** *ZFAS1* occupancy at the *SMARCA2* loci from the *ZFAS1* ChIRP-seq data.

**(B)** RNAPIIo (*top*: CTD-ser5P; *bottom*: CTD-ser2P) distribution changes around the *SMARCA2* promoter region after UV-C irradiation in the wild-type and the *ZFAS1*-depleted cell lines.

**(C)** Same as in (A), but the *XPC* gene loci is presented.

**(D)** Same as in (B), but the *XPC* gene promoter region is shown.

**(E)** *ZFAS1* binding sites upon UV-C irradiation enriched for several sequence motifs that are nearly identical to the four transcription factor motifs (*KLF5*, *p53*, *PAX7*, and *RarA*).

**(F)** Percentage of interaction sites containing both *ZFAS1* and RNAPIIo. Red bars, the proportion of the *ZFAS1*-bound genes with downregulated CTD-ser5P or upregulated CTD-ser2P within 6 hrs after UV-C irradiation in the wild type cell line of all genes with changed RNAPIIo upon UV-C. Blue bars, the proportion of the *ZFAS1*-bound genes containing either downregulated CTD-ser5P or upregulated CTD-ser2P within 6 hrs after UV-C exposure of all the *ZFAS1*-bound genes. White bars, the proportion of *ZFAS1*-bound genes with changed RNAPIIo only detected in the wild type cell line but not in the *ZFAS1*-depleted cell line of all the *ZFAS1*-bound genes with changed RNAPIIo.

**(G)** Venn diagram showing the overlap between the *ZFAS1*-binding sites and the CPD hyper-hotspots after UV-C irradiation.

**Figure S14. Identification of UV-induced *ZFAS1* interactors, Related to Figure 5**

**(A)** Venn diagram showing the number of the *ZFAS1* interactors, identified by ChIRP-MS in the presence or absence of UV-C irradiation in MRC5_VA cells.

**(B)** Top six GO-terms for the *ZFAS1* interactors. BP, biological process; CC, cellular component.

**(C)** UV-induced *ZFAS1* interactome analyzed by RNA pull-down experiments using biotinylated *ZFAS1* in UV-C irradiated MRC5_VA cell extracts. For clarity, the common proteins also identified by ChIRP are indicated in blue, all the other interactors are labeled in grey.

**(D)** qPCR detection of *ZFAS1* retrieved by a H3.3-specific antibody in the RIP assay in MRC5_VA cells with or without UV-C irradiation. Each data bar is presented as the means ± SD from three independent experiments. ***p* < 0.05, ***p* < 0.01 compared to control IgG or undisturbed cells (Student’s *t*-Test).

**(E)** Western Blot images (*left*) and quantification (*right*) of H3.3 in the wild-type (WT) and *ZFAS1*-depleted MRC5_VA cells at 3 h after UV-C irradiation. Values are means ± SD, n = 3 independent experiments. ***p* < 0.01 (Student’s *t*-Test).

**(F)** Average plots of read densities for H3.3 on all genes from 2 kb upstream of TSS to TSS + 2 kb, before (UV-) and after (UV+) UV-C irradiation for the WT cells and *ZFAS1*-depleted cells.

**(G)** Distribution of H3.3 binding sites at the defined genomic regions.

**(H)** Venn diagram showing overlap of genes that harbor significant enrichment of H3.3 after UV damage and *ZFAS1* targets post-irradiation.

**(I)** Cell viability of the WT, CS-B, *ZFAS1*-depleted cells and H3.3 overexpressed *ZFAS1*-knockdown cells exposed to the indicated doses of UV-C irradiation and recovered for 3 days. Untreated cells were used as controls. Each data point is presented as the means ± SD, n = 3. **p* < 0.05, ***p* < 0.01 compared to the *ZFAS1*-depleted cells (Student’s *t*-Test). CS1AN cell line was used as a positive control for TC-NER-deficient cell line.

**(J)** *top*: representative images of cells showing repair of CPDs at the indicated recovery time post UV irradiation using a specific antibody to CPDs (green signal) for the wild-type, *ZFAS1*-depleted cells and *ZFAS1*-knocked down MRC5_VA cells overexpressing H3.3. DAPI-stained nuclei in blue. Scale bar = 50 μm. *bottom*: histogram plots of average CPD signals at the indicated recovery time points following UV-C irradiation.

**Figure S15. Overexpression of mouse *Zfas1* facilitates UV-induced DNA repair, Related to Figure 6**

**(A)** The expression levels of mouse lncRNA *Zfas1* in various mouse tissues during embryonic development or postnatal stages. All expression data are normalized to mouse 18S RNA and presented as means ± SD from three independent experiments.

**(B)** Illustration of the full-length mouse *Zfas1* transcripts validated by RACE.

**(C)** Survival assay of mouse NIH3T3 cells overexpressing *Zfas1* exposed to UV-C irradiation at the indicated doses. Untreated cells were used as controls. Each data point is presented as the means ± SD, n = 3. ***p* < 0.01 compared to the WT cells (Student’s *t*-Test).

**(D)** *top*: representative images of cells showing repair of CPDs at the indicated recovery time post UV irradiation using a specific antibody to CPDs (green signal) for the wild-type and mouse NIH3T3 cells overexpressing *Zfas1*. DAPI-stained nuclei in blue. Scale bar = 50 μm. *bottom*: histogram plots of average CPD signals at the indicated recovery time points following UV-C irradiation.

**Figure S16. In *vivo* characterization of *Zfas1* genotypes, Related to Figure 6**

**(A)** Expression of *Zfas1* and *Znfx1* genes in MEFs of indicated genotype, measured by RT-qPCR. Data are normalized to mouse 18S RNA and shown as the means ± SD, n = 3 for each genotype. ***p* < 0.05 (Student’s *t*-Test).

**(B)** 8-week-old *Zfas1* KO mice exhibit similar body weight compared to the littermate WT controls. Data are presented as means ± SD analyzed with the Student’s *t*-Test. Male and female, n = 6:6.

**(C)** Representative images of kidneys from 8-week-old female mice at experimental end point. Scale bar = 1 cm.

**(D)** *left*: representative haematoxylin and eosin-stained (HE) sections of kidneys of mice. G indicates glomeruli. Typical examples of normal and abnormal glomeruli are shown. Scale bar = 50 μm. *right*: on one kidney section per animal of the indicated genotype the percentage of abnormal glomeruli was determined. Data are presented as means ± SD and were analyzed with the Student’s *t*-Test; **p* < 0.05, ***p* < 0.01. male and female, n = 3:3.

**(E)** Representative images of the EdU incorporation for the WT and *Zfas1^-/-^* mouse fibroblasts treated with UV-C irradiation (10 J/m^2^). Scale bar = 50 μm. White arrows indicate non-S-phase cells with unscheduled DNA synthesis (UDS) occurring.

**(F)** Representative images of primary cells showing repair of 6-4PPs using a specific antibody to 6-4PP (green signal) for the WT and *Zfas1^-/-^* mice. DAPI-stained nuclei in blue. Scale bar = 25 μm.

**Table S1**. FPKM values for all the transcripts (including mRNAs and lncRNAs) detected in the study from RNA-seq Data in the synchronized MRC5_VA cells released from the G1/S phase for the indicated time intervals, Related to **Figure 1**.

**Table S2**. Clustering and GO term Enrichment analysis of mRNAs for RNA-seq Data derived from non-irradiated G1/S-phase-synchronized MRC5_VA cells at the indicated intervals after release, Related to **Figure S2**A.

**Table S3**. Clustering analysis of mRNAs for RNA-seq Data derived from UV-irradiated G1/S-phase-synchronized MRC5_VA cells at the indicated intervals after release, Related to **Figure S2**C.

**Table S4**. GO term enrichment analysis of DEGs at 3 h and 72 h after UV-C irradiation, related to **Figure S2**C.

**Table S5**. Clustering and co-localization Enrichment analysis of lncRNAs for RNA-seq data derived from the non-irradiated synchronized MRC5_VA cells released from the G1/S block, Related to **Figure 2**A.

**Table S6**. Clustering and co-localization and co-expression Enrichment analysis of lncRNAs for RNA-seq data derived from the UV-irradiated synchronized MRC5_VA cells released from the G1/S block, Related to **Figure 2**B.

**Table S7**. The list of peaks called by MACS from RNAPIIo ChIP-seq in the WT and *ZFAS1*-depleted cells treated with 10 J/m^2^ UV-C irradiation, Related to **Figures 5**I-J.

**Table S8**. The list of peaks (raw peaks) called by MACS from *ZFAS1* ChIRP-seq in MRC5_VA cells treated with 10 J/m^2^ UV irradiation, Related to **Figure 6**.

**Table S9**. The list of peaks (true peaks) called by the stringent pipeline from *ZFAS1* ChIRP-seq in MRC5_VA cells treated with 10 J/m^2^ UV irradiation, Related to **Figure 6**.

**Table S10**. The list of Gene Ontology (GO) terms of the genes with the identified filtered *ZFAS1* ChIRP-seq peaks (true peaks) in the wild type and UV-irradiated cells, Related to **Figure 6**.

**Table S11.** The list of differential peaks called by MACS from H3.3 ChIP-seq in the WT cells treated with 10 J/m^2^ UV-C irradiation, Related to **Figure S15**.

**Table S12**. List of oligos and antibodies used in the study.

**Figure S1**


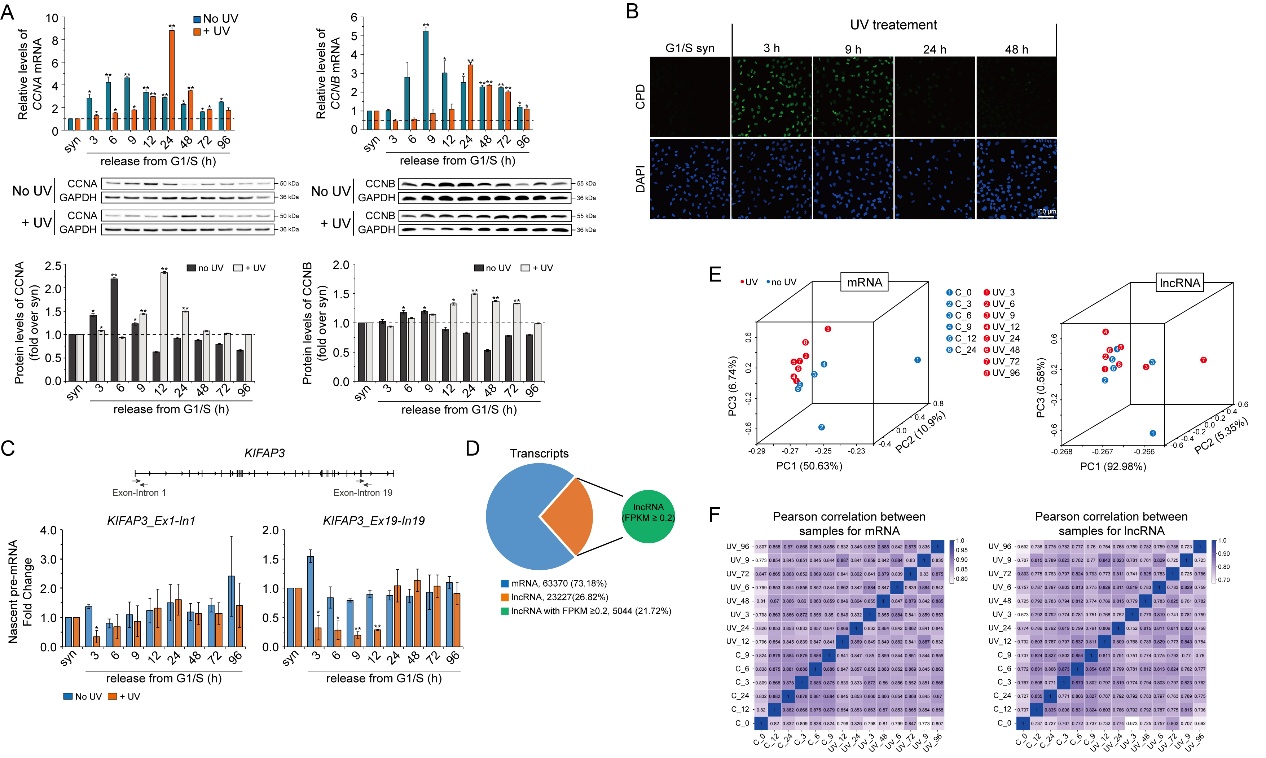


**Figure S2**


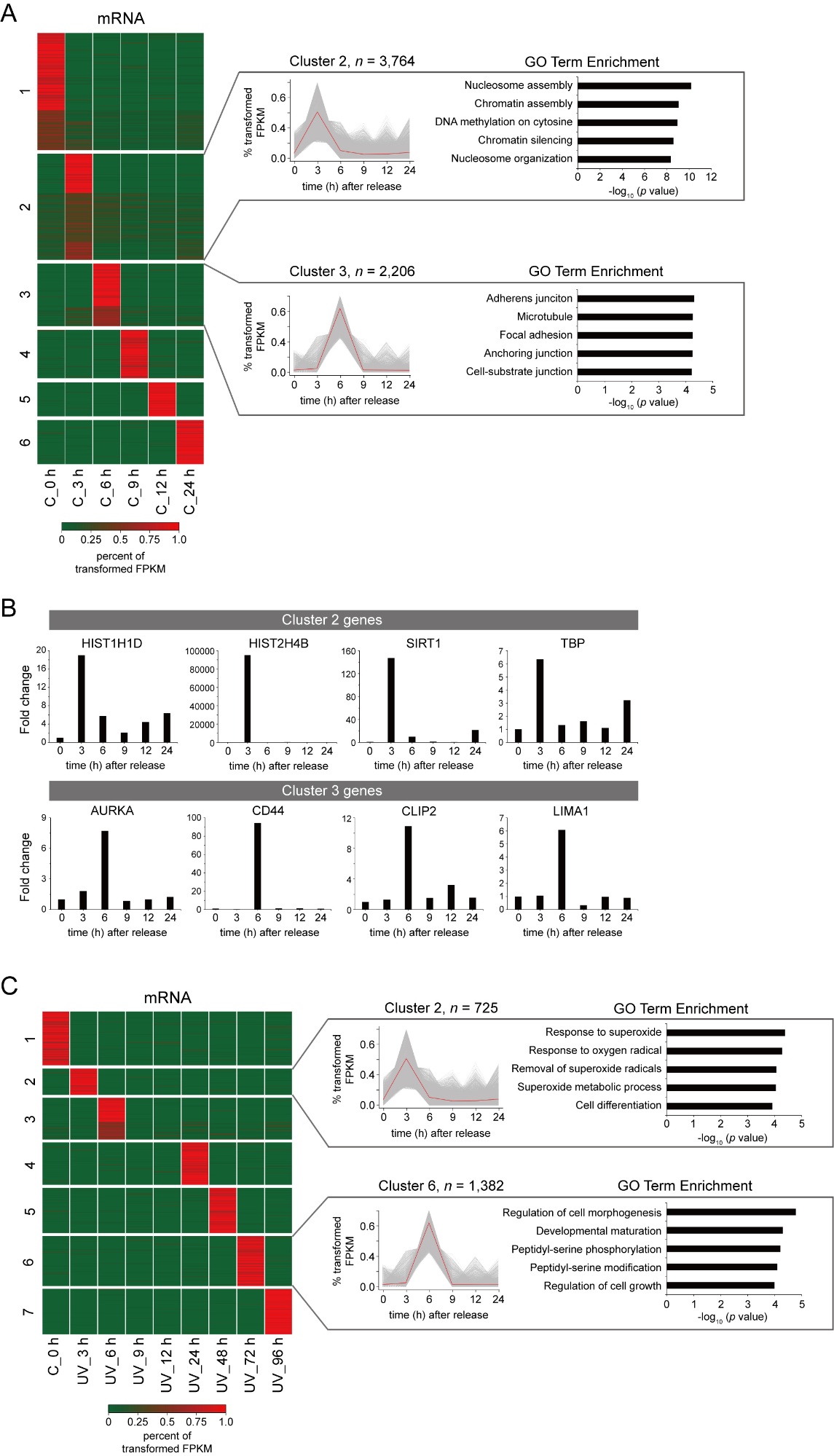


**Figure S3**


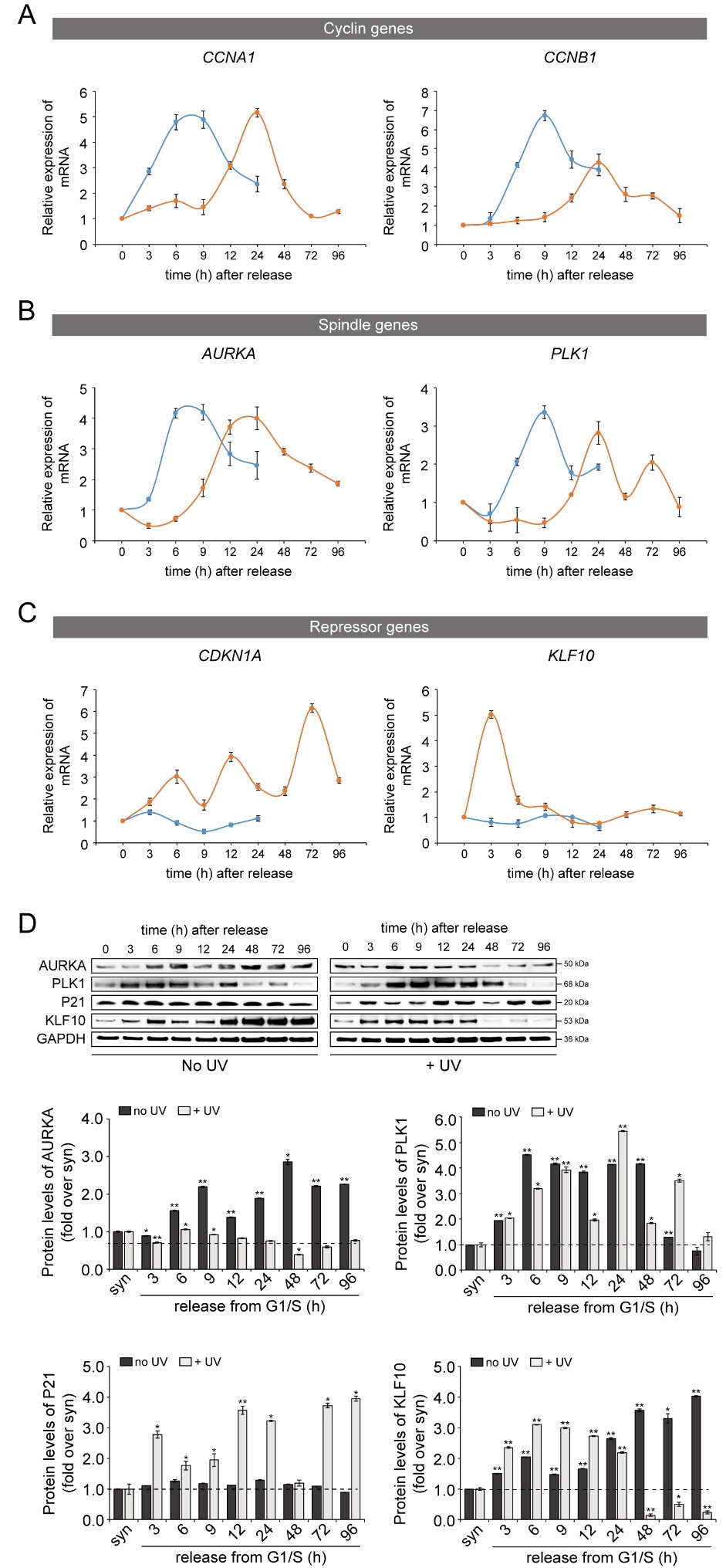


**Figure S4**


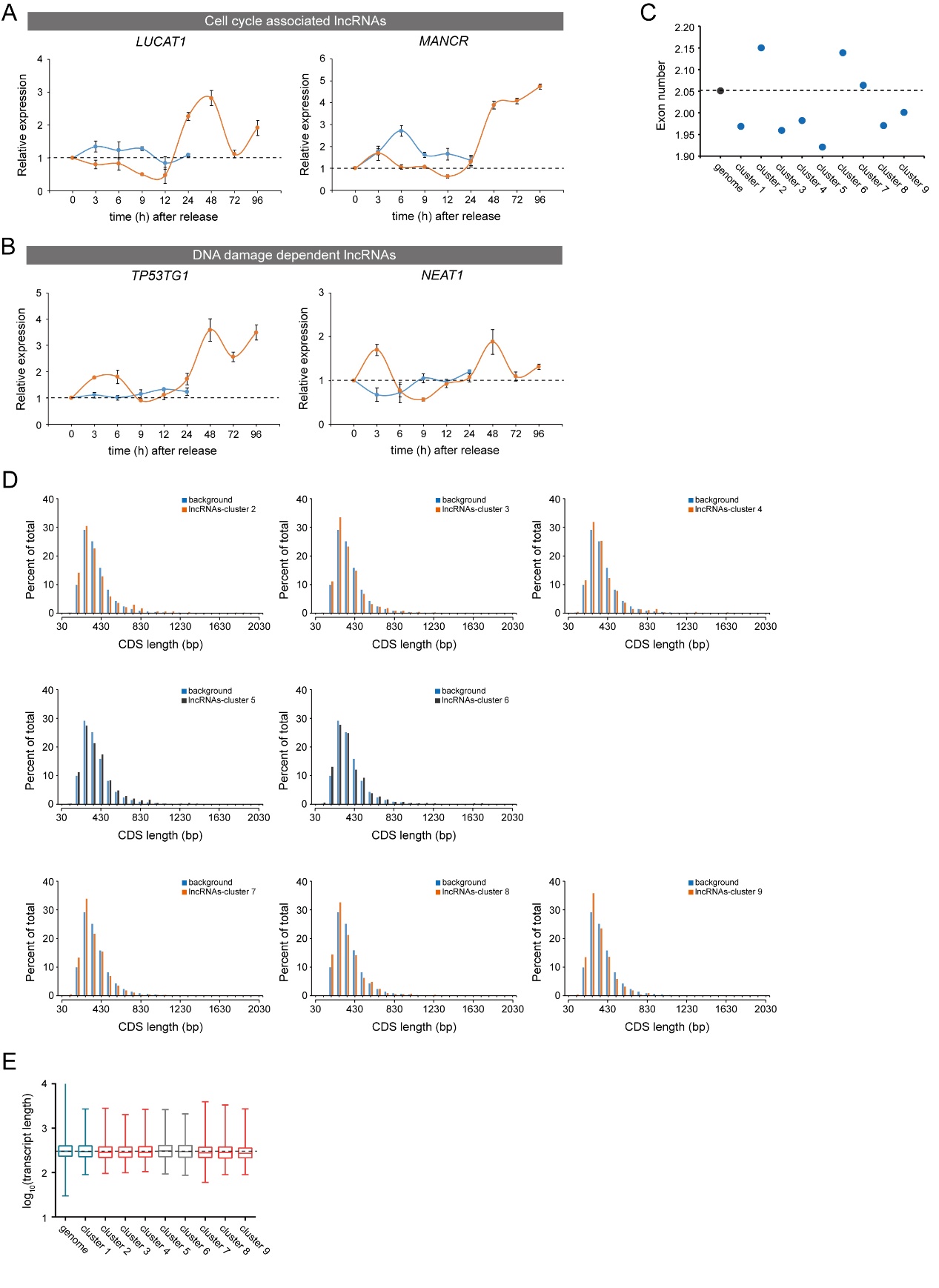


**Figure S5**


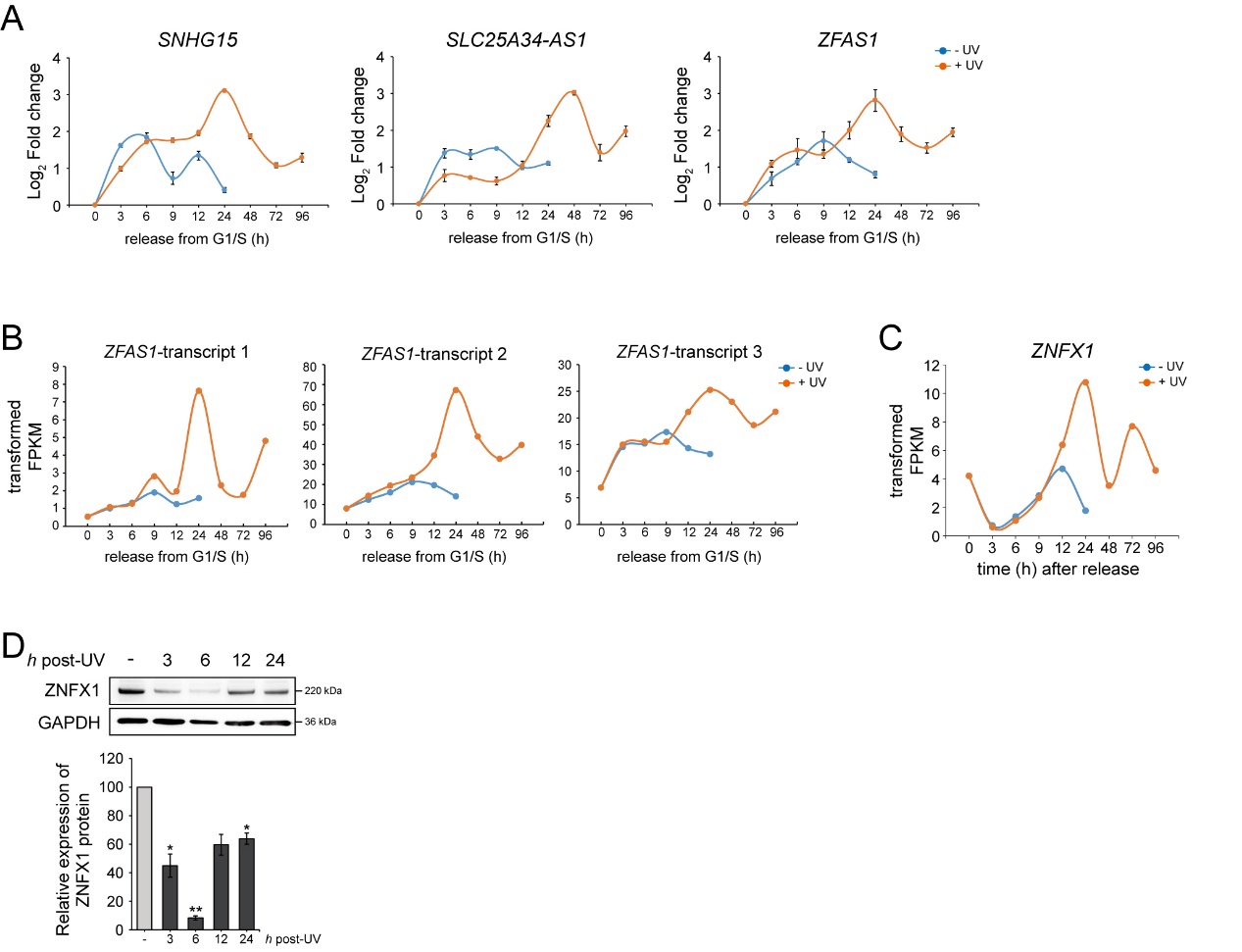


**Figure S6**


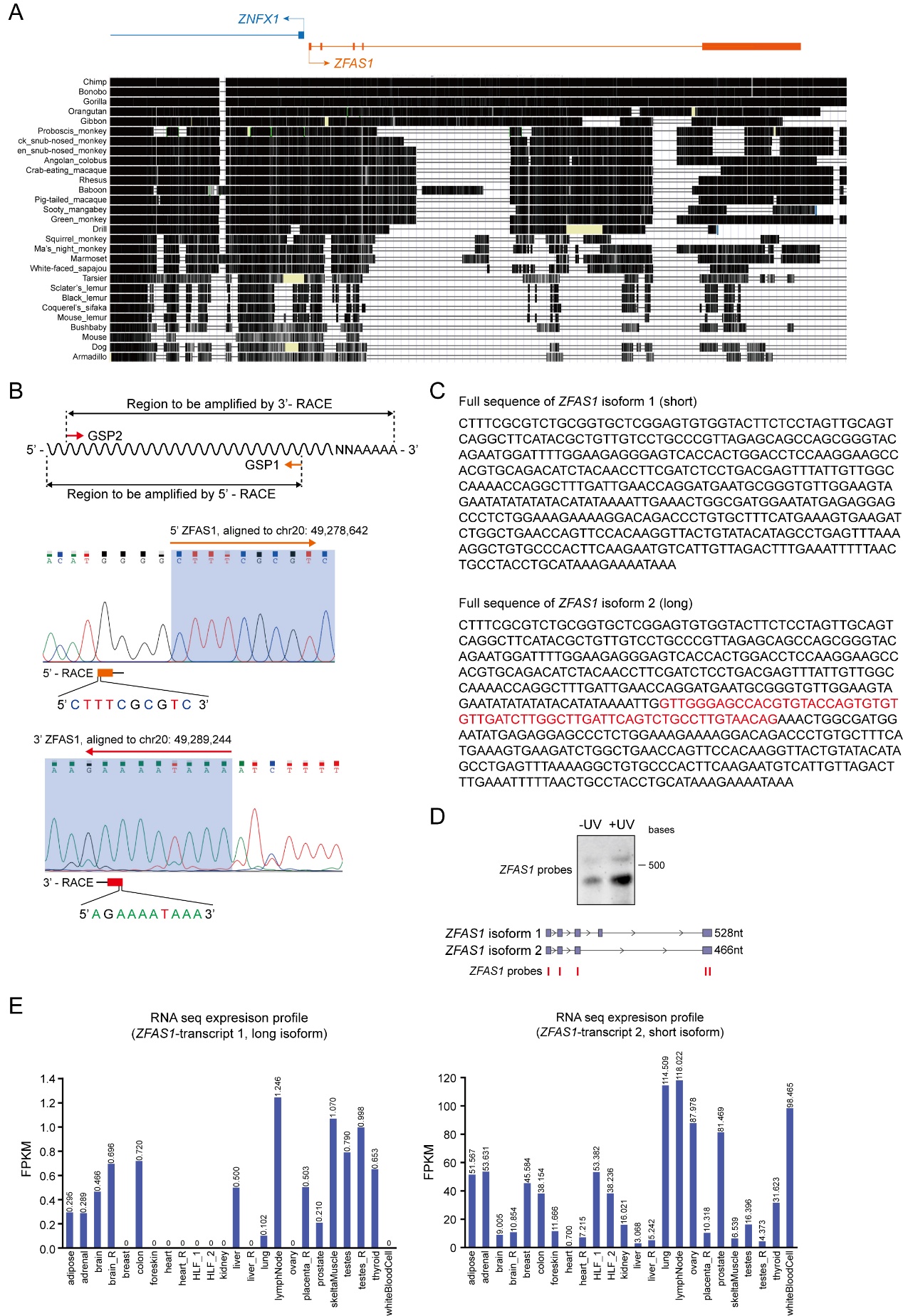


**Figure S7**


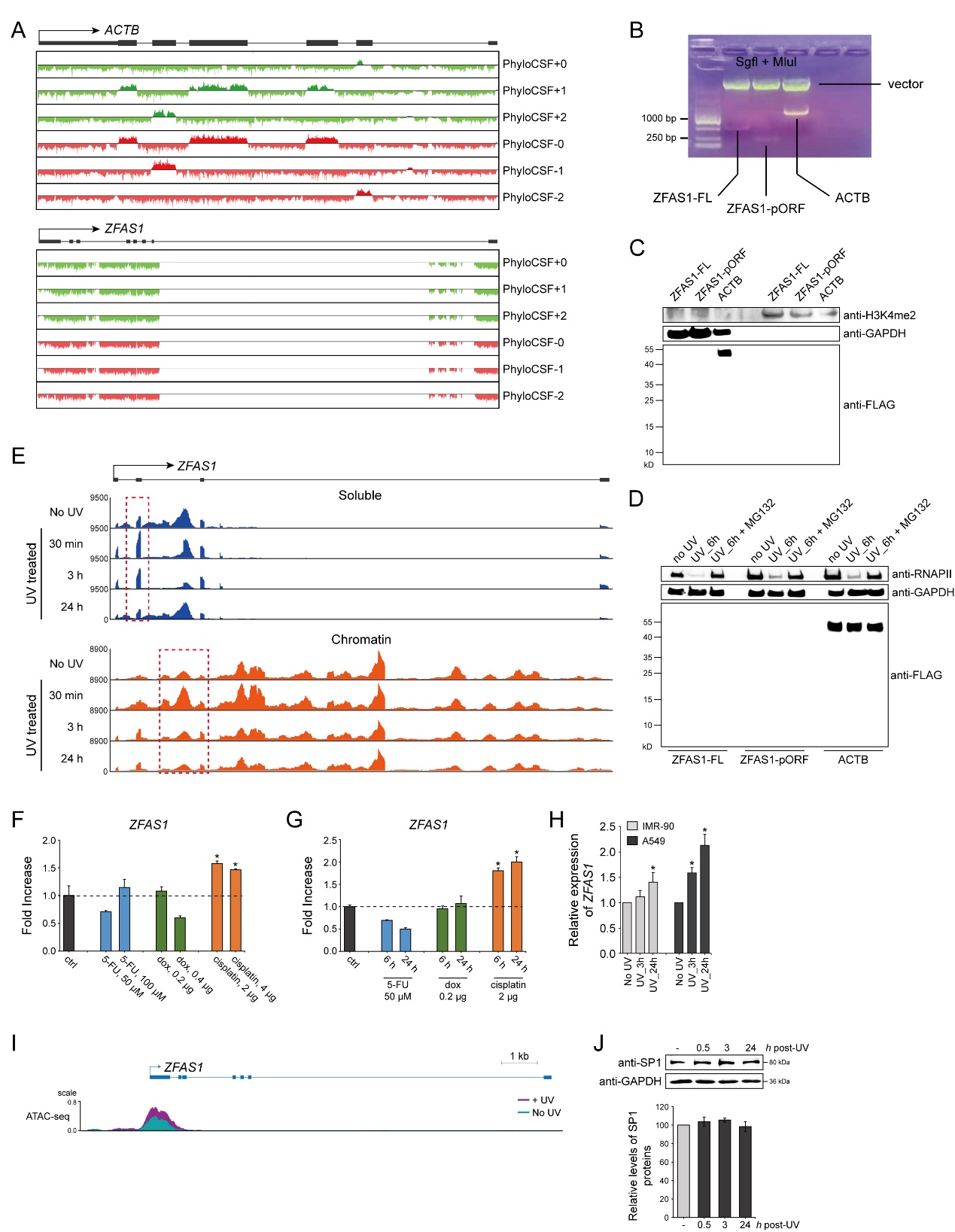


**Figure S8**


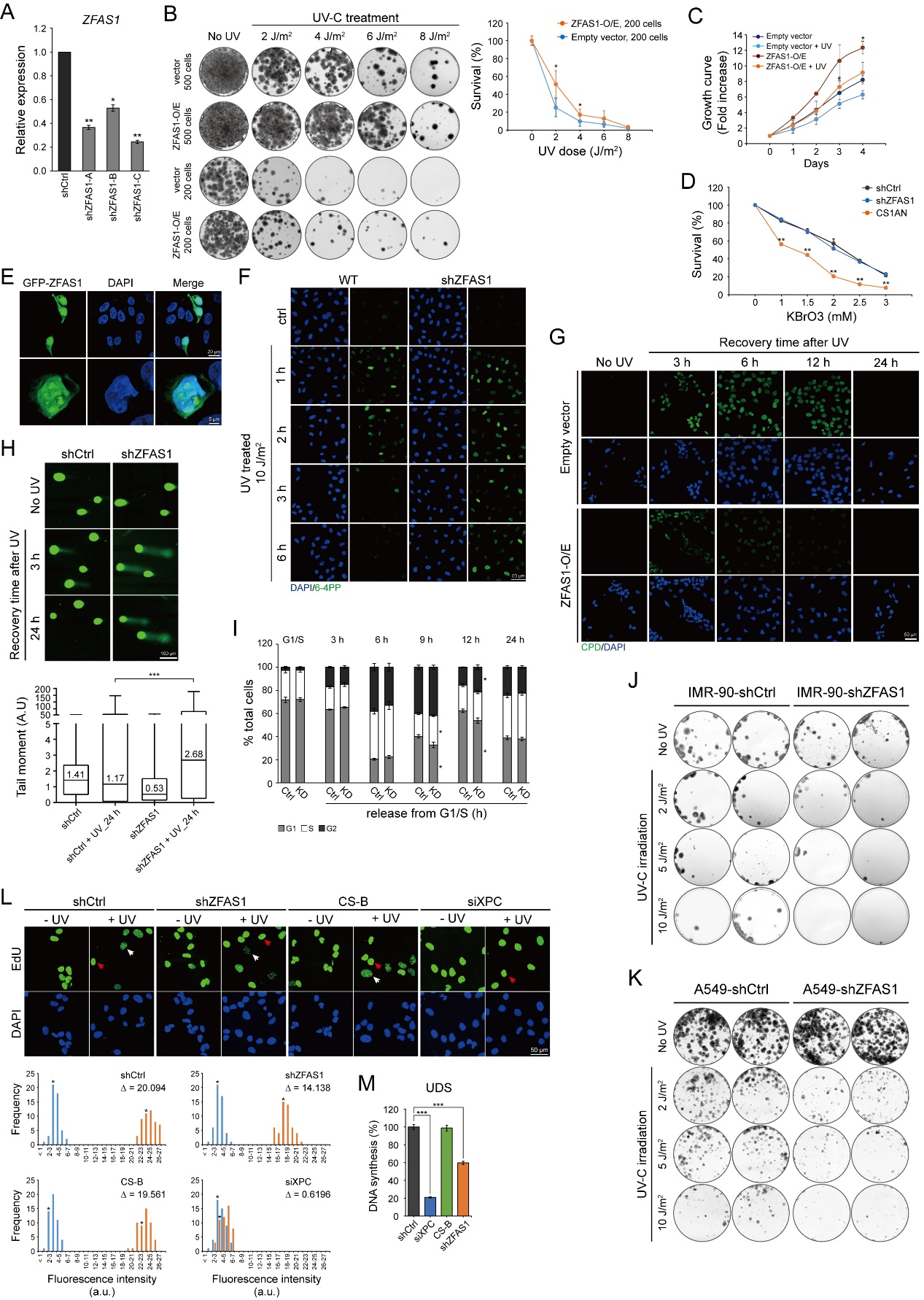


**Figure S9**


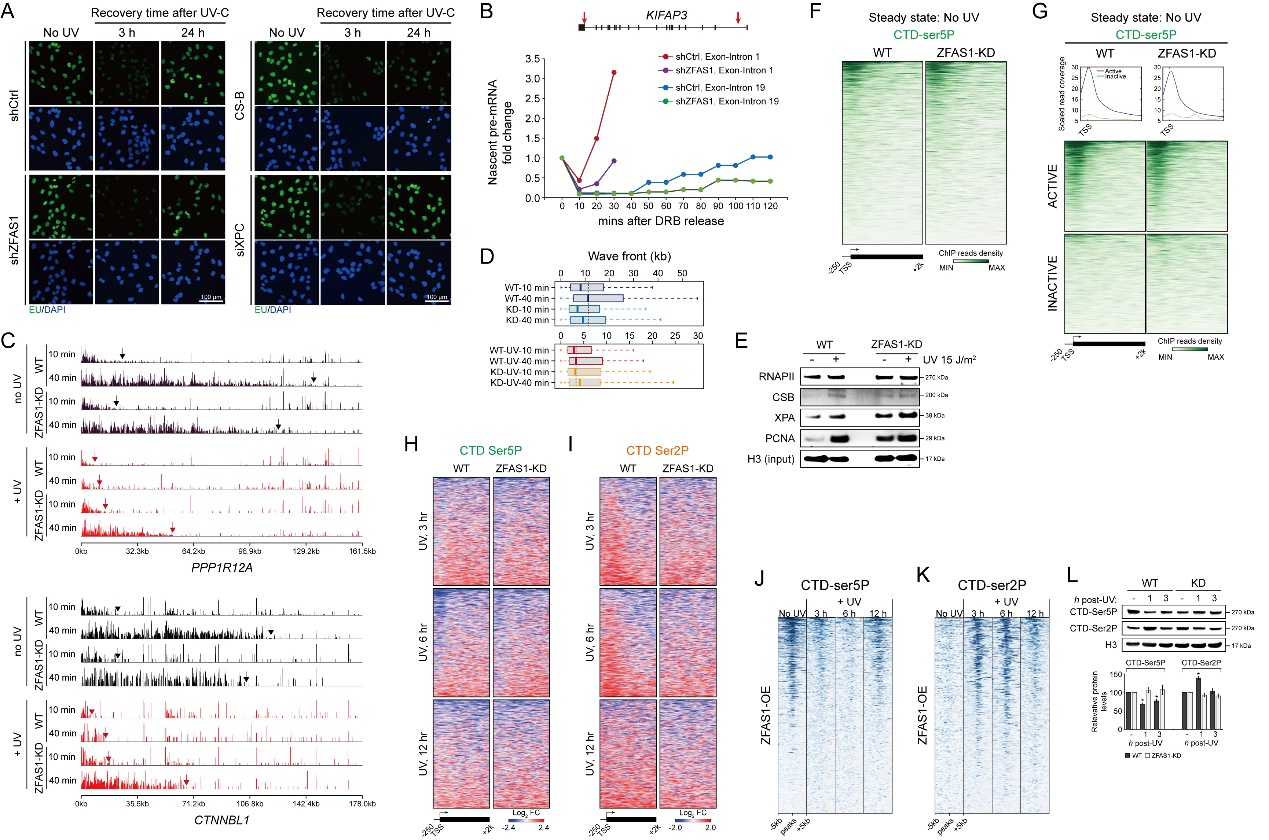


**Figure S10**


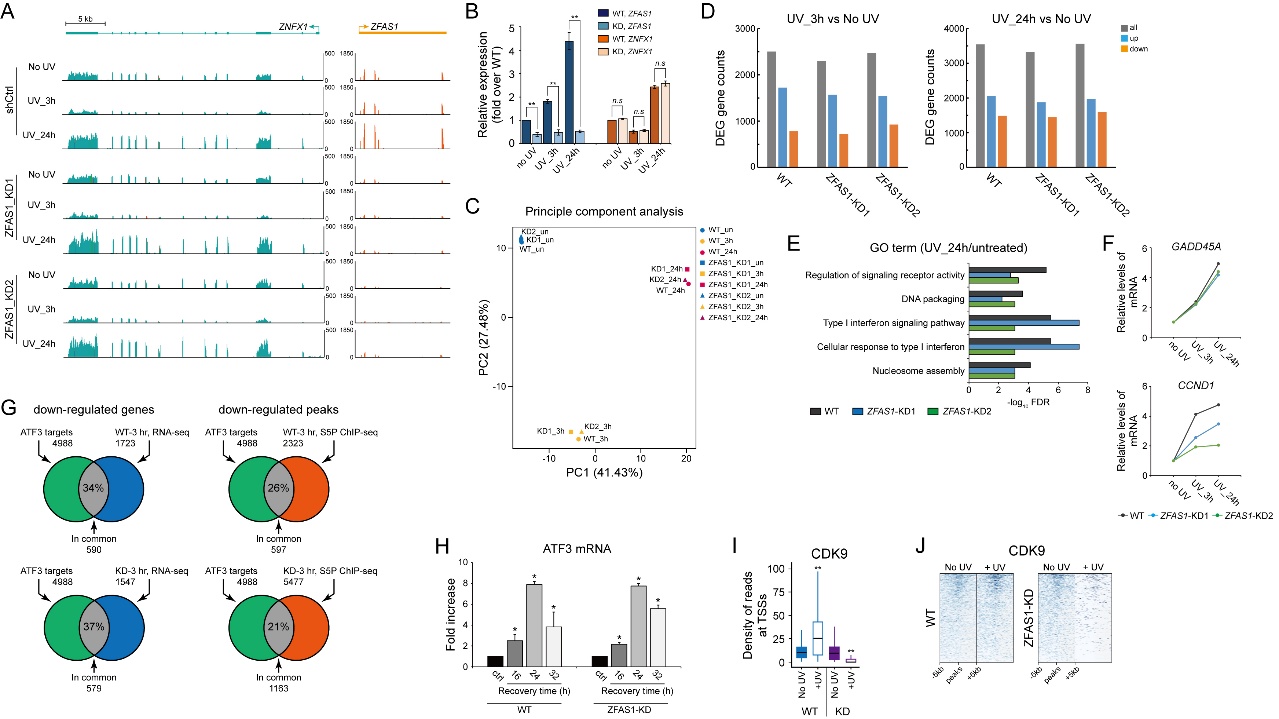


**Figure S11**


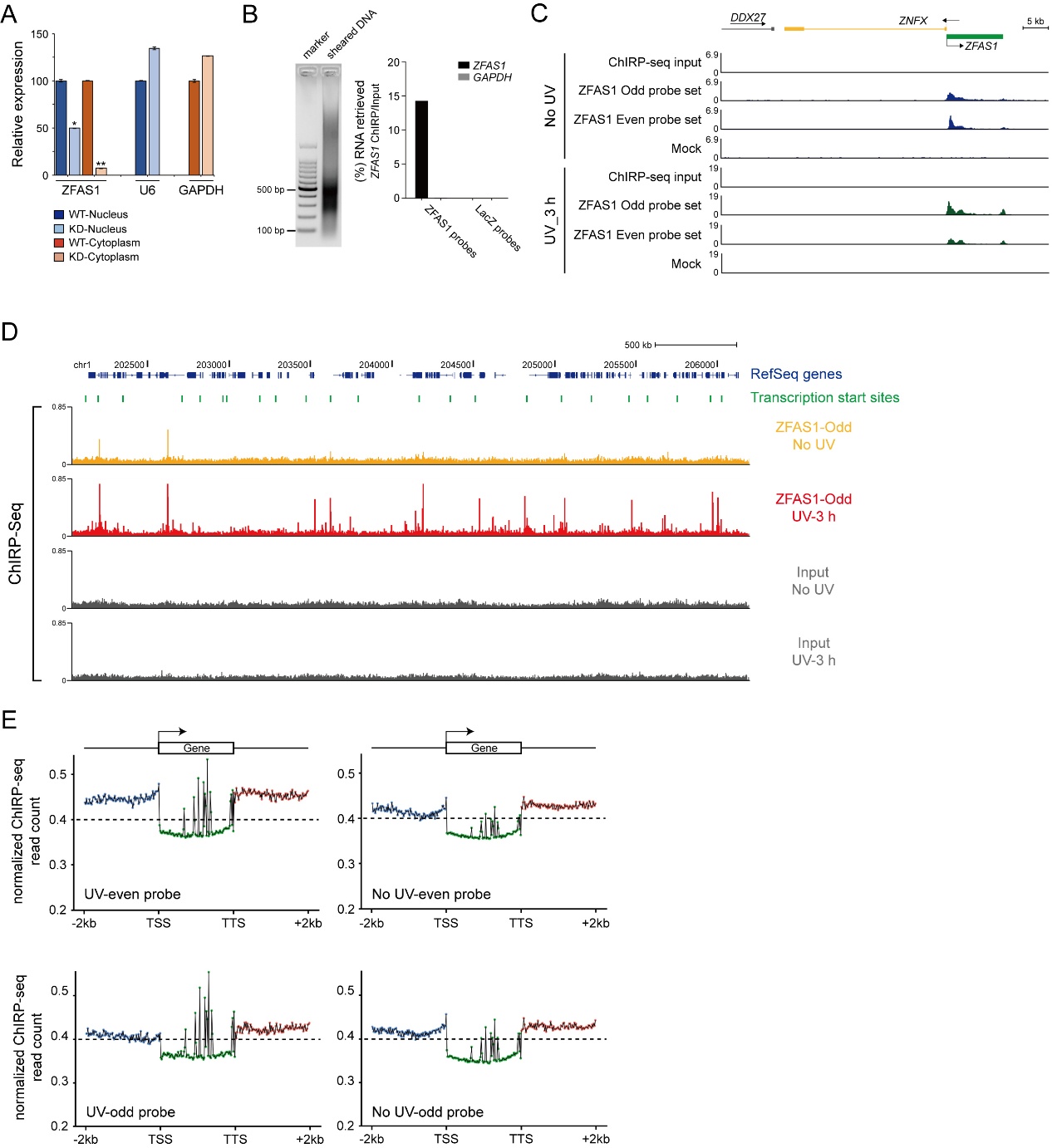


**Figure S12**


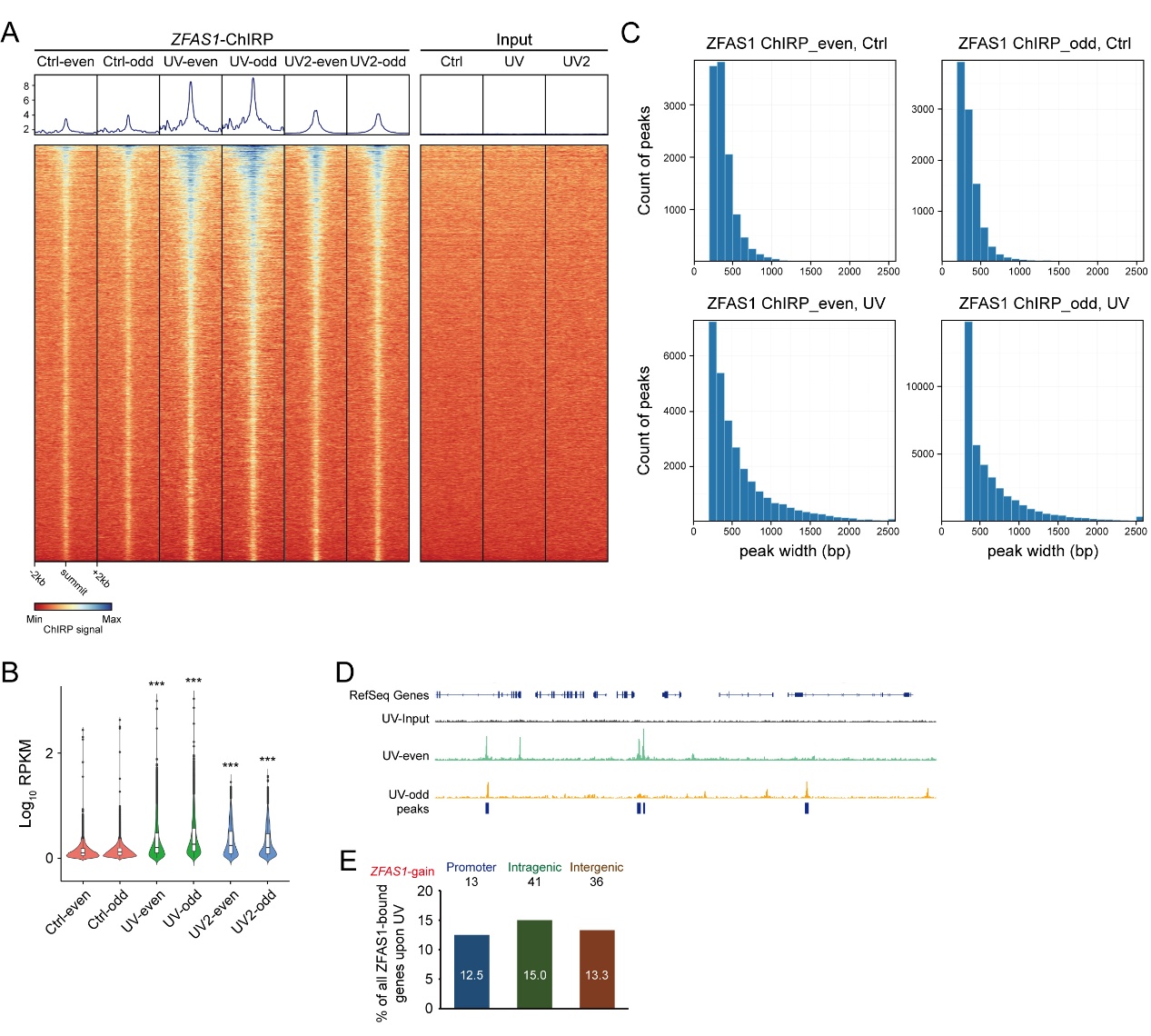


**Figure S13**


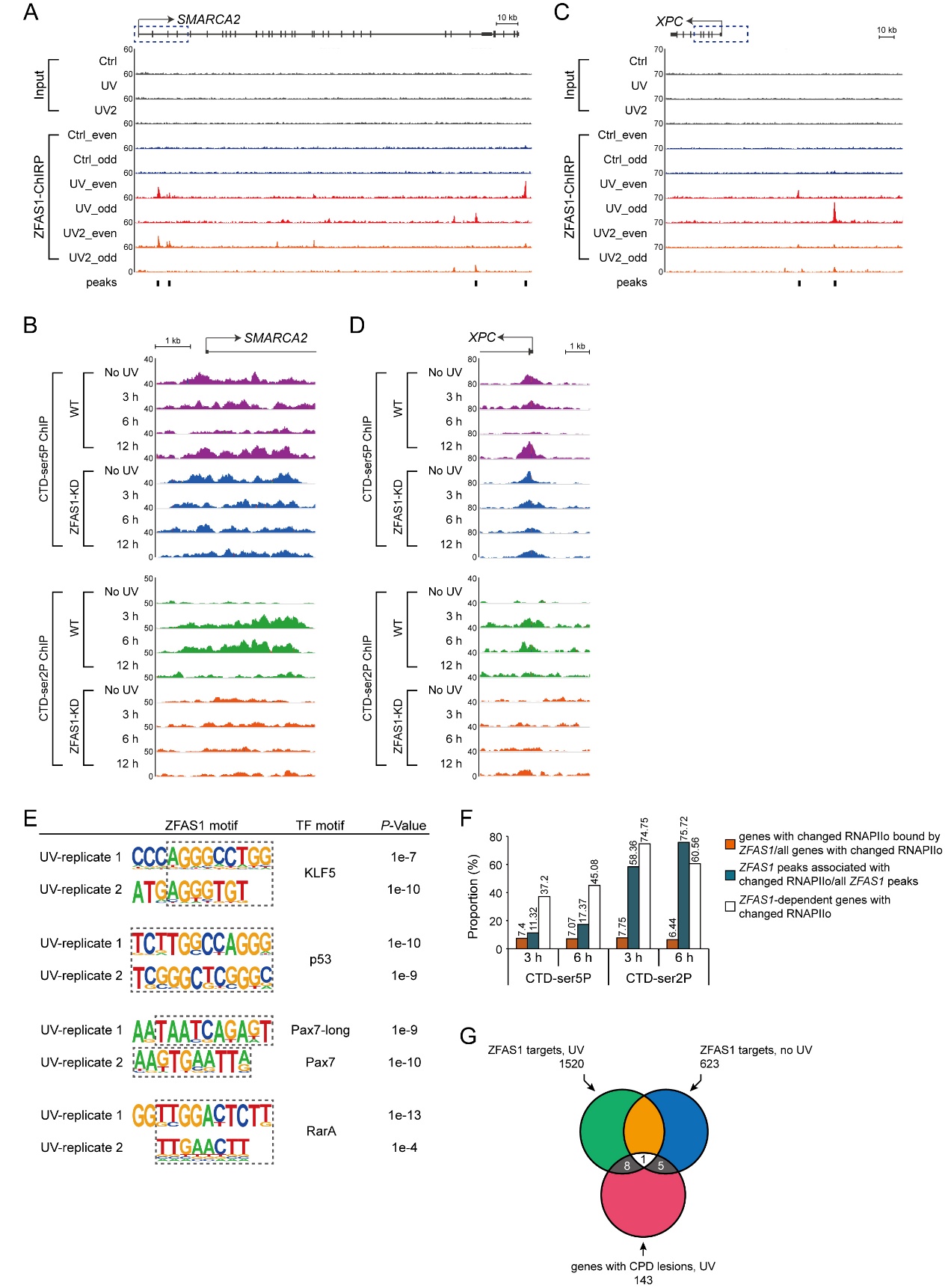


**Figure S14**


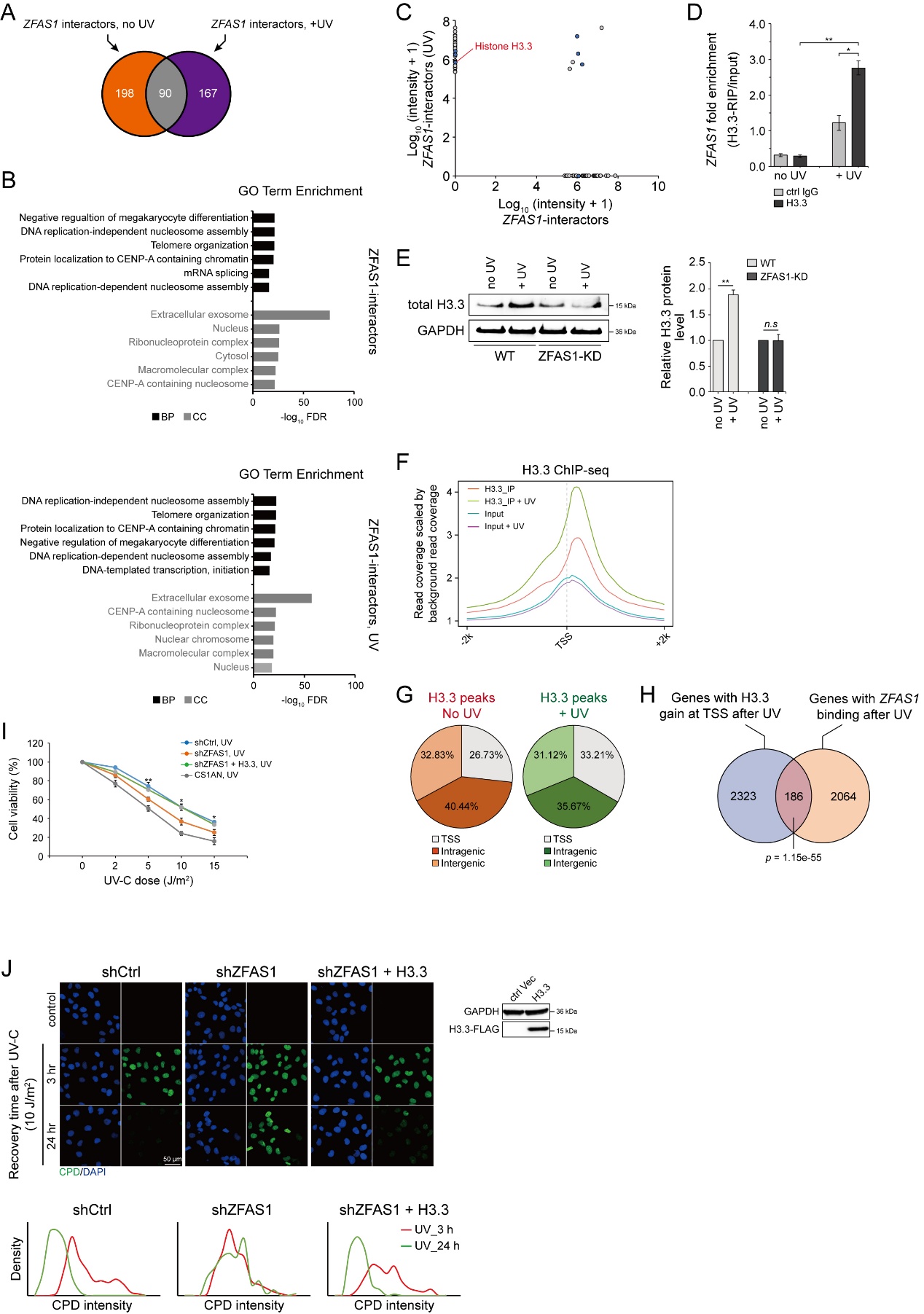


**Figure S15**


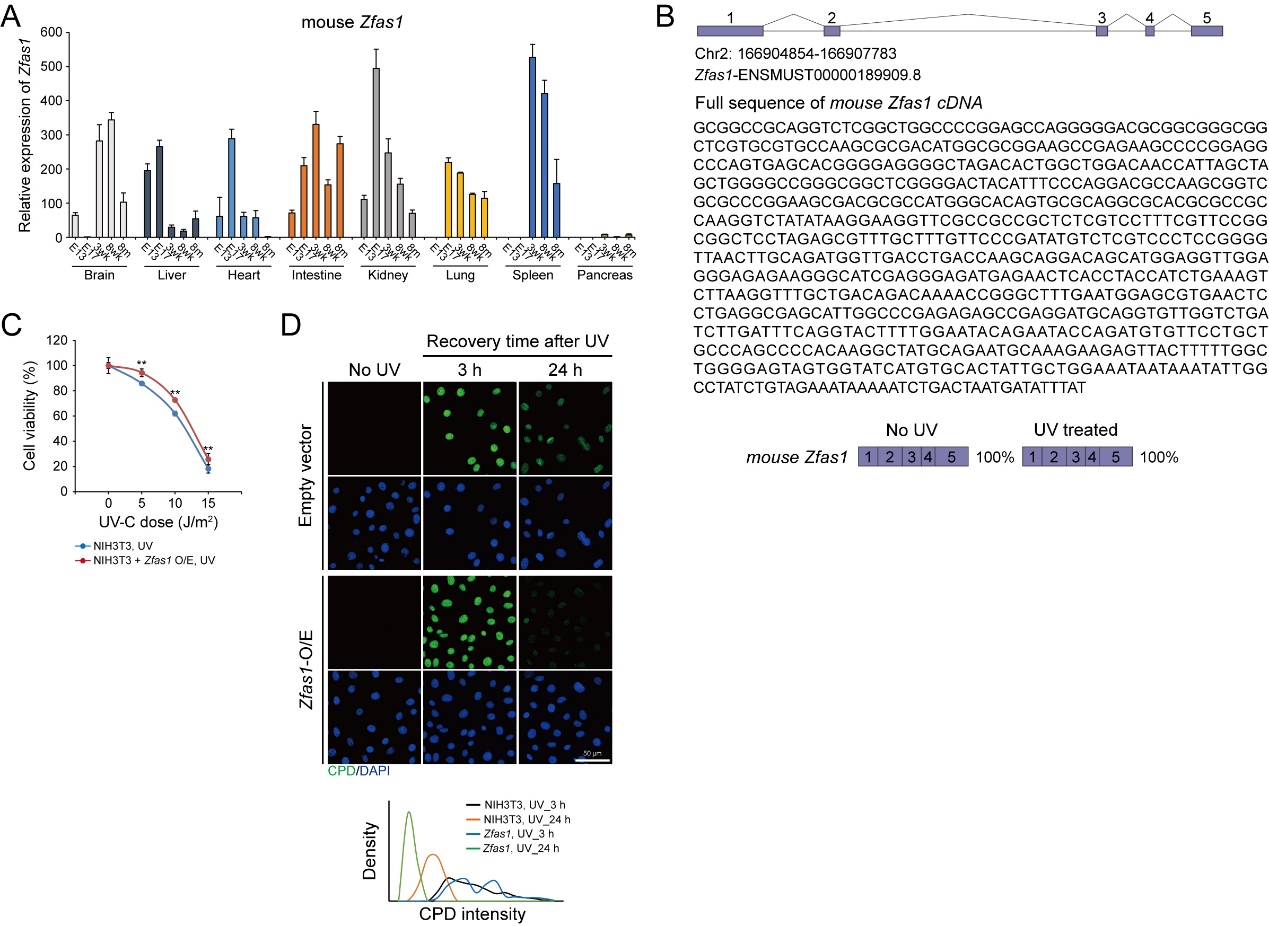


**Figure S16**


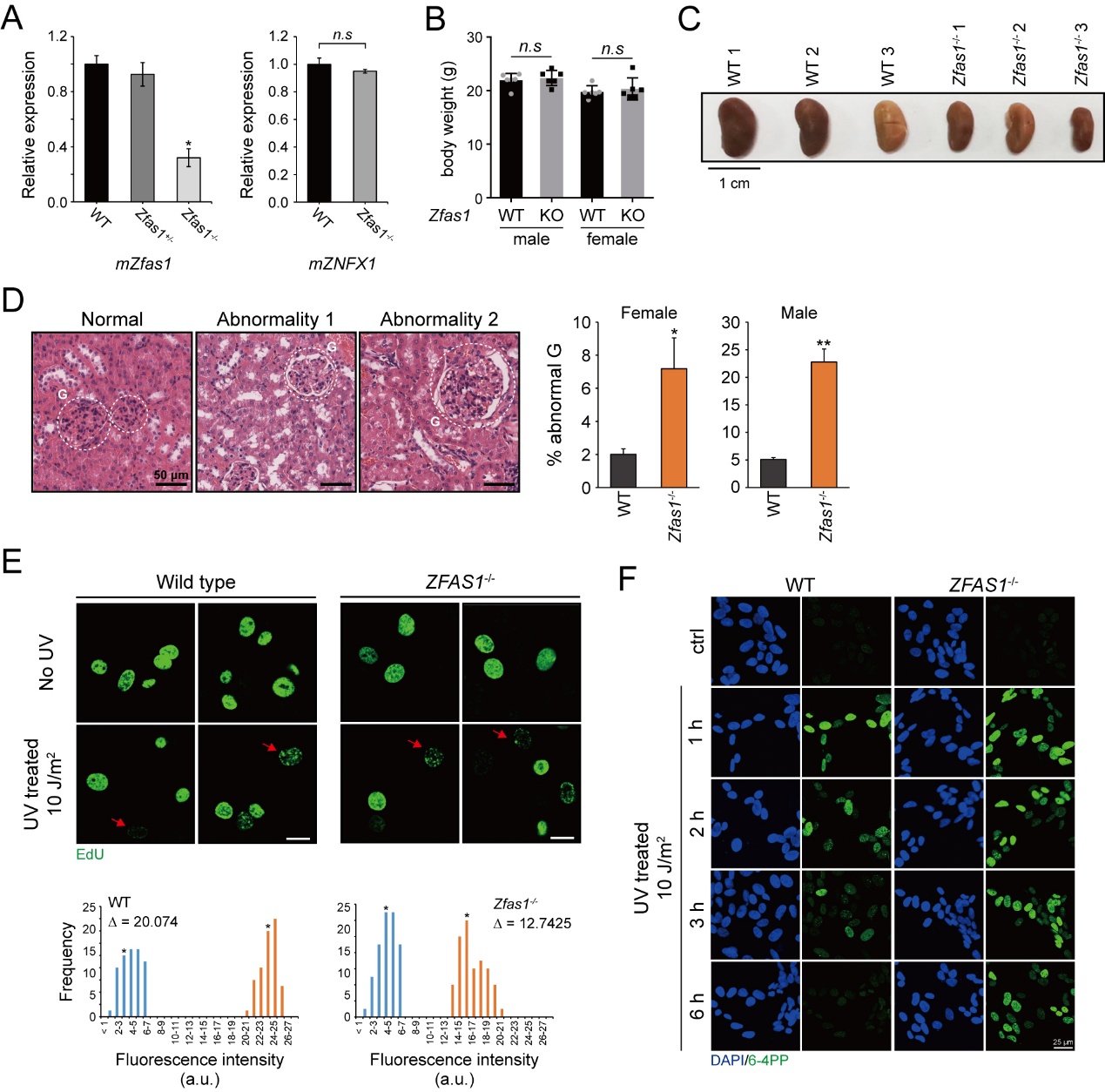

Supplement: Supplementary file 1 — Supporting Information [file ADVS-12-e12385-s004.docx]
